# Insertion of CS_2_ into Bi–N Bonds: Coordination Chemistry and Reactivity of Bismuth Dithiocarbamates

**DOI:** 10.1021/acs.inorgchem.6c03443

**Published:** 2026-09-30

**Authors:** Gargi Kundu, Frerik Wurm, Kai Oberdorf, Ahmed Fetoh, Pulkit Dhankhar, Celina Becker, Johanna Heine, Kerstin Volz, Crispin Lichtenberg

**Affiliations:** † Department of Chemistry, Philipps-University Marburg, Hans-Meerwein-Str. 4, D-35032 Marburg, Germany; ‡ mar.quest | Marburg Center for Quantum Materials and Sustainable Technologies, D-35032 Marburg, Germany; § Department of Chemistry, Faculty of Science, Mansoura University, Dakahlia Governorate, 11432 Mansoura, Egypt; ∥ Institute of Chemistry, Carl von Ossietzky Universität Oldenburg, Ammerländer Heerstraße 114-118, D-26129 Oldenburg, Germany; ⊥ Materials Science Center and Department of Physics, Philipps-University Marburg, Hans-Meerwein-Str. 6, D-35032 Marburg, Germany

## Abstract

The straightforward access to simple halo­(bismuth)­amides bearing the core structural motif [Bi­(NMe_2_)*_n_* X_3–*n*_] (X = Cl, Br, I; *n* = 1, 2) has been investigated, along with their coordination chemistry and their reactivity toward CS_2_. The latter revealed selective insertion reactions to give literature-known and new bismuth compounds with the dithiocarbamate (S_2_CNMe_2_)^−^ ligand scaffold. Their behavior toward a cyclic­(alkyl)­(amino) carbene (CAAC) has been studied, demonstrating that redox processes are less relevant than in simple bismuth halides and halo­(bismuth)­amides and that the coordination of the CAAC ligand dominates instead. Exposing bismuth dithiocarbamates to reducing conditions revealed the complex anion [Bi­(S_2_CNMe_2_)_4_]^−^, an unprecedented structural motif in pnictogen chemistry. The rational synthesis of [Li­(py)_4_]­[Bi­(S_2_CNMe_2_)_4_] provided synthetic access to this motif. Finally, the potential of the neutral species [Bi­(S_2_CNMe_2_)_3_] as a single-source precursor for the temperature-induced formation of Bi_2_S_3_ has been investigated.

## Graphical abstract

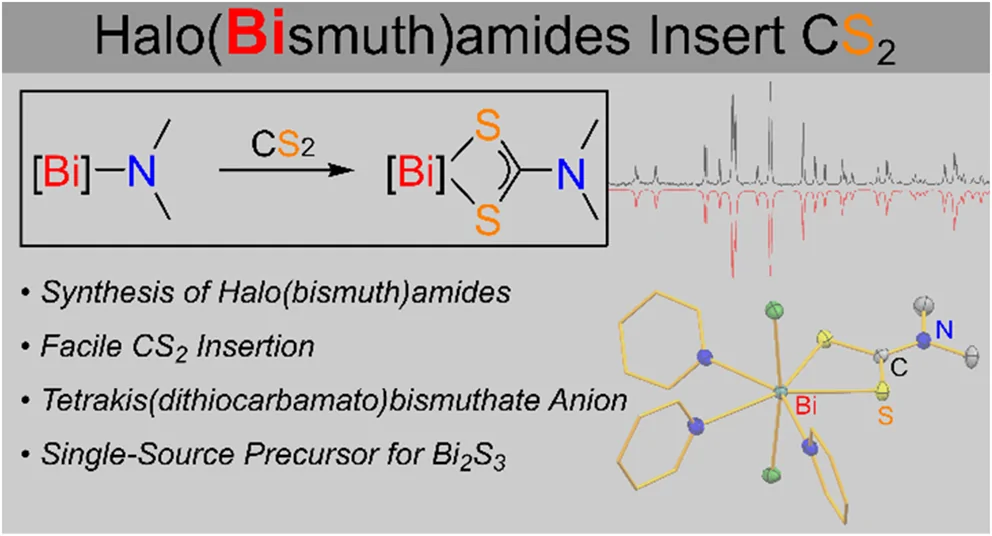

## Introduction

Carbon disulfide (CS_2_) is a versatile building block for the synthesis of sulfur-containing compounds in organic chemistry, coordination chemistry, and materials science. In industrial settings, it finds application in the manufacturing of viscose fibers from cellulose and for the synthesis of xanthates that are utilized as flotation agents in mineral processing.^1,2^ In academic research, the insertion of CS_2_ into metal–ligand bonds is an important, atom economic transformation that facilitates its exploitation as a chemical building block. Consequently the insertion of carbon disulfide into M–X bonds across large parts of the periodic table of the elements has been studied (M = metal, X = H, C, N, O, Si).^3–17^ Among this wide range of reactions, the insertion chemistry of the heavy main group element bismuth with CS_2_ has received surprisingly little attention. While reactions of bismuth compounds with various small molecules such as H_2_, C_2_H_4_, SF_6_, CO, CO_2_ and other heterocumulenes have recently been investigated,^7,9,18–36^ the understanding of CS_2_ insertion into Bi–X bonds is lagging behind. An early study reported the insertion of CS_2_ into the Bi–N bonds of Bi­(NMe_2_)_3_, but the product was only analyzed by ^1^H NMR spectroscopy, elemental analysis, IR and UV/vis spectroscopy at the time.^33^ Furthermore, a remarkable case of reversible CS_2_ insertion into a Bi–S bond has been reported (Scheme 1a).^6^ An excess of CS_2_ is necessary to facilitate the selective insertion reaction (**A** + CS_2_ → **B**). At elevated temperatures (starting at *T* = 147 °C), CS_2_ could be extruded from **B** and the starting material **A** is regenerated. In a related case, the rapid insertion of CS_2_ into the Bi–N bond of a cationic species such as **C** with a ring-strained structural motif^37–39^ was observed to give compound **D** (Scheme 1b).^7,35^ Exposure of compound **D** to ambient light or irradiation (λ = 254 nm) eliminated the Bi­(I) species Bi­(OTf) (**E**) to give benzothiazolethione **F**, containing CS_2_ as a building block in the photochemical construction of organic heterocycles.

**Scheme 1:**
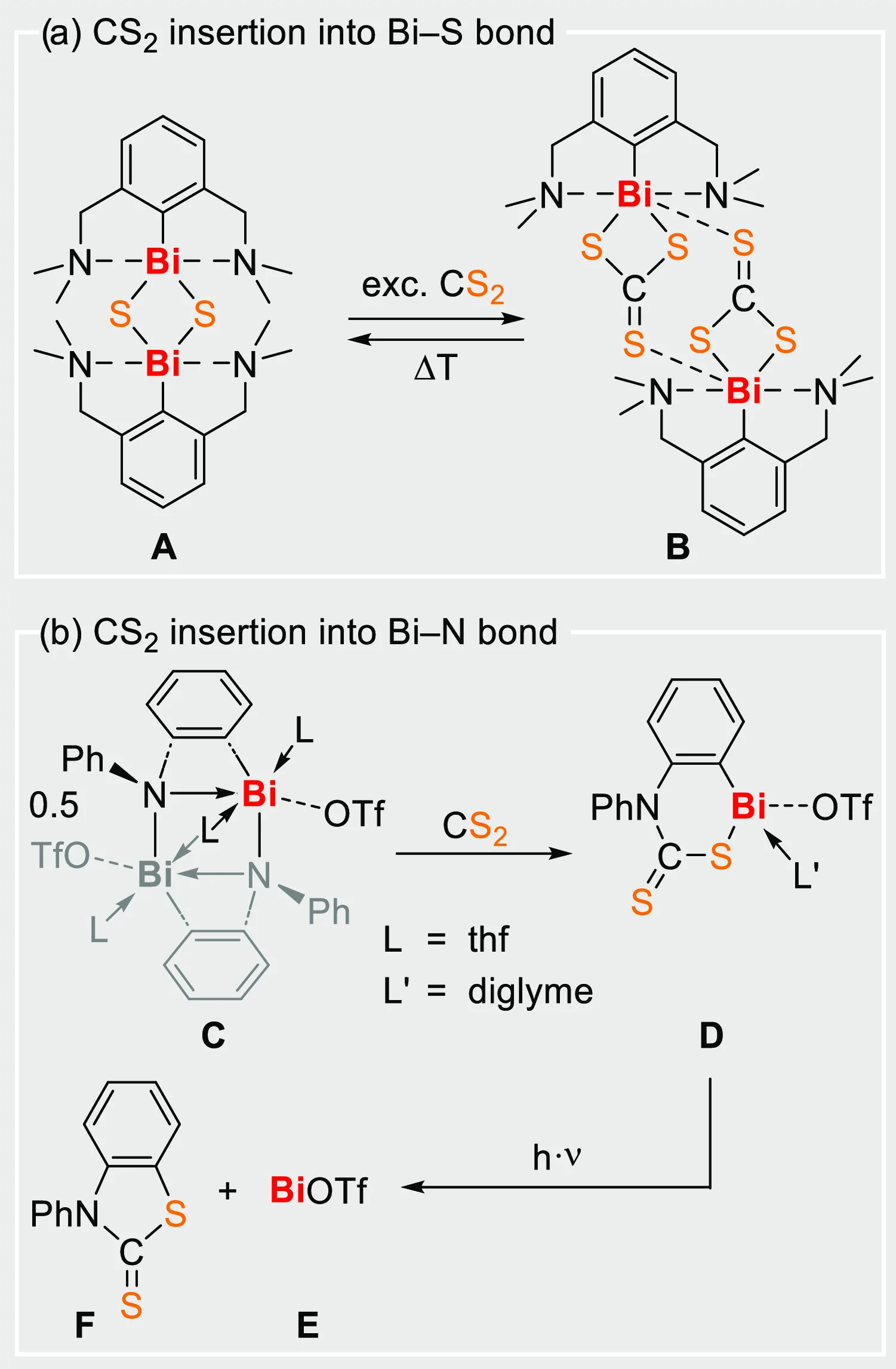
Rare Examples of Thoroughly Investigated CS_2_ Insertion Reactions with Molecular Bismuth Compounds with Insertions into a Bi–S (a) and a Bi–N Bond (b)

In the context of using CS_2_ as a building block in bismuth chemistry, bismuth dithiocarbamates are attractive targets. Such compounds (accessed via alternative synthetic routes) have been applied as single-source precursors (SSPs) for the generation of Bi_2_S_3_, a nontoxic semiconductor with a band gap that shows a good fit with the solar spectrum.^40,41^ Compared to alternative approaches (e.g., solid state synthesis, solvothermal synthesis, chemical bath deposition) SSPs generally bring about potential advantages such as a predefined stoichiometry, the requirement to control less parameters, and the avoidance of autoclave conditions. However, SSPs for the synthesis of Bi_2_S_3_ currently suffer from two-step thermal degradation,^42,43^ product formation in a very broad temperature window,^43^ and nonquantitative conversion.^44^

We became interested in the insertion of CS_2_ into the Bi–N bonds of simple bismuth complexes without the introduction of charged species or specific ligand design and aimed to test one of the simplest bismuth dithiocarbamates for its ability to act as a SSP for Bi_2_S_3_. Here we report the synthesis of a small series of simple halo­(bismuth)­amides, insertion reactions of Bi­(NMe_2_)*_n_* X_3–*n*_ (*n* = 1–3; X = halide) with CS_2_, and the investigations of the resulting products in coordination chemistry and Bi_2_S_3_ generation.

## Results and Discussion

While a range of homoleptic bismuth amides Bi­(NR_2_)_3_ has been reported,^33,39,45–48^ only an extremely small number of simple bismuth compounds Bi­(NR_2_)*_n_* X_3–*n*_ (R = H, alkyl, silyl, aryl; X = halide; *n* = 1, 2) with monodentate amide ligands is known, and their reactivity is poorly explored.^49–57^ The reaction of one equivalent of Bi­(NMe_2_)_3_ (**1**) with two equivalents of BiX_3_ (X = Cl, Br, I) in pyridine at room temperature afforded the dihalo­(bismuth)­amide species [BiCl_2_(NMe_2_)­py]_2_ (**2-Cl**), [BiBr_2_(NMe_2_)­py]_2_ (**2-Br**) and [BiI_2_(NMe_2_)­py]_2_ (**2-I**) as green (**2-Cl**, **2-Br**) and red (**2-I**), crystalline solids (Scheme 2, left). In analogy with the recently reported bromo analog,^50^ the remaining chloro- and iodo­(bismuth)­diamides [BiCl­(NMe_2_)_2_]_∞_ (**3-Cl**) and [BiI­(NMe_2_)_2_]_∞_ (**3-I**) could be isolated as yellow (**3-Cl**) and orange (**3-I**) solids from a comproportionation reaction between two equivalents of Bi­(NMe_2_)_3_ (**1**) and one equivalent of BiX_3_ (X = Cl, I) in pyridine (Scheme 2, right). Compounds **2-X** and **3-X** (X = Cl, Br, I) are air- and moisture-sensitive. Compounds **2-X** did not show a pronounced sensitivity toward ambient light. However, the compounds **3-X** were found to be light-sensitive (similar to Bi­(NMe_2_)_3_), as evidenced by the formation of a dark solid upon exposure to ambient light for more than 3 h as a solid or in solution.

**Scheme 2:**
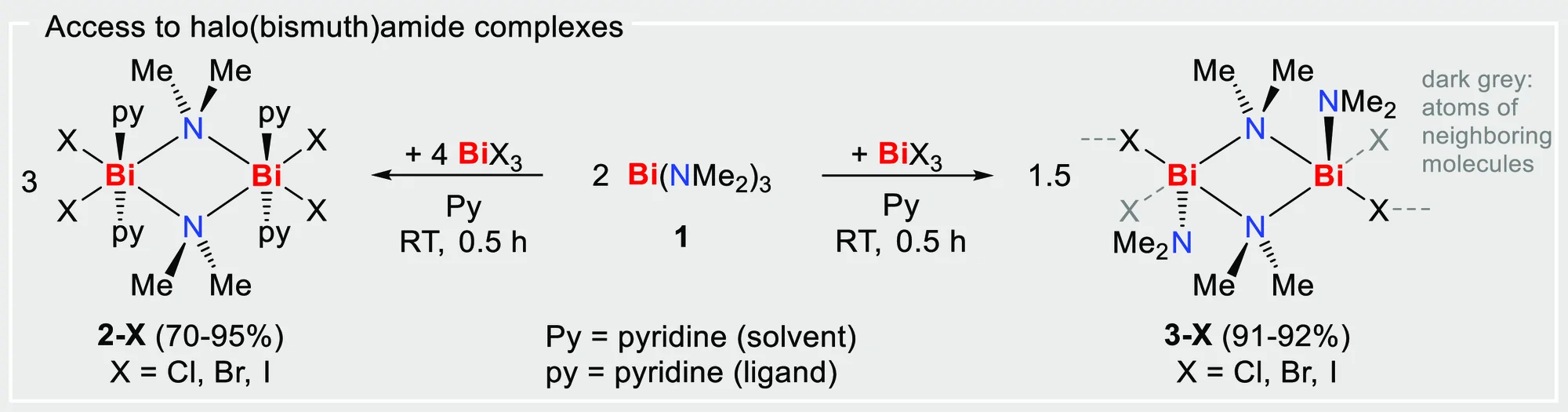
Synthesis of the Halo­(bismuth)­amides **2-X** (X = Cl, Br, I) and **3-X** (X = Cl, Br, I)^a^

a Compound **3-I** crystallizes as monopyridine adduct in a dimeric form (*vide infra*) that releases its pyridine ligands *in vacuo* to give the pyridine-free compound depicted above.

Compounds **2-Cl**, **2-Br**, **3-Cl** and **3-I** were structurally characterized by single-crystal X-ray diffraction analysis (Figure 1).

**Figure 1:**
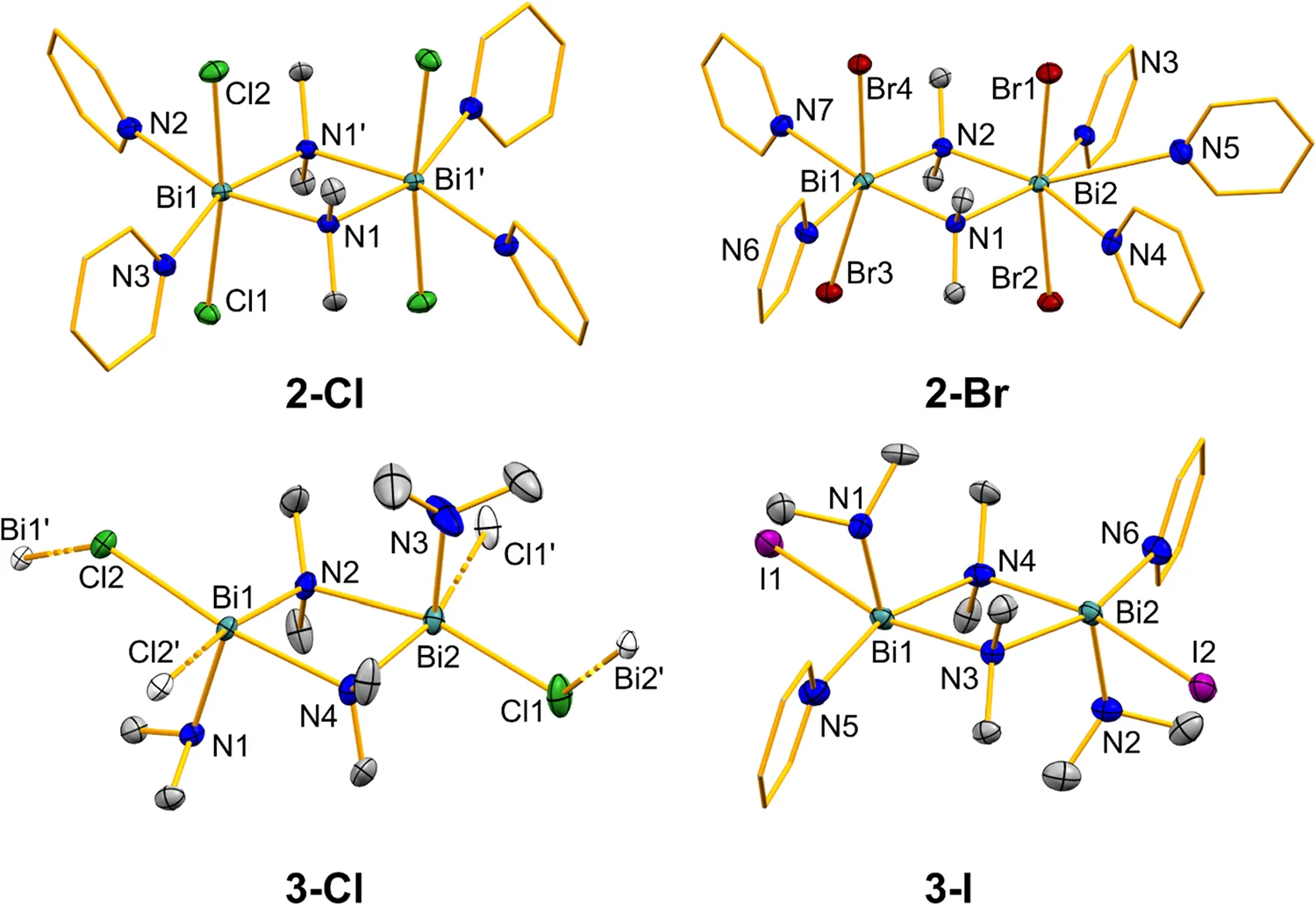
Molecular structure of **2-Cl**, **2-Br**, **3-Cl** and **3-I** in the solid state. Displacement ellipsoids are shown at 50% probability, hydrogen atoms are omitted for clarity, carbon atoms of pyridine ligands are shown as wireframe. Compounds **2-Br** and **3-I** show noninteracting lattice bound pyridine molecules which are omitted for clarity. Selected bond distances [Å], angles [deg] and torsion angles [deg]. Compound **2-Cl**: Bi1–Cl1 2.6668(6), Bi1–Cl2 2.6714(6), Bi1–N1 2.3626(16), Bi1–N1′ 2.3537(18), Bi1–N2 2.7541(17), Bi1–N3 2.7855(19), Cl1–Bi1–Cl2170.703(17), N1–Bi1–N1′ 77.33(6), Bi1–N1′–Bi1′–N1 0.00. Compound **2-Br**: Bi1–Br3 2.8155(4), Bi1–Br4 2.8788(4), Bi2–Br1 2.7583(4), Bi2–Br2 2.9091(4), Bi1–N1 2.343(3), Bi1–N2 2.358(3), Bi1–N6 2.798(3), Bi1–N7 2.838(3), Bi2–N1 2.441(3), Bi2–N2 2.411(3), Bi2–N3 2.744(3), Bi2–N4 2.776(3), Bi2–N5 3.058(3), Br3–Bi1–Br4 163.378(13), Br1–Bi2–Br2 171.277(13), N1–Bi1–N2 77.53(11), N1–Bi2–N2 74.69(10), Bi1–N2–Bi2–N1 3.92. Compound **3-Cl**: Bi1–Cl2 2.7835(7), Bi1–Cl2′ 3.1770(10), Bi2–Cl1 2.8612(9), Bi2–Cl1′ 3.0708(10), Bi1–N1 2.143(3), Bi1–N2 2.294(2), Bi1–N4 2.406(3), Bi2–N3 2.106(3), Bi2–N2 2.396(2), Bi2–N4 2.325(3), N2–Bi1–N4 77.20(9), N2–Bi2–N4 76.80(9), Cl2–Bi1–N2 88.40(6), Cl2–Bi1–N4 165.57(7), N1–Bi1–N2 94.56(10), N1–Bi1–N4 88.54(9), N1–Bi1–Cl2 88.40(6), Bi1–N2–Bi2–N4 14.25. Compound **3-I**: Bi1–I1 3.3222(7), Bi2–I2 3.3151(7), Bi1–N1 2.135(7), Bi1–N3 2.383(6), Bi1–N4 2.347(7), Bi1–N5 2.828(8), Bi2–N2 2.158(7), Bi2–N3 2.347(6), Bi2–N4 2.390(7), Bi2–N6 2.799(8), N3–Bi1–N4 76.3(2), N3–Bi2–N4 76.2(2), I1–Bi1–N4 85.49(18), I1–Bi1–N3 161.66(16), N1–Bi1–N4 104.3(2), N1–Bi1–N3 92.3(2), N1–Bi1–I1 90.02(18), Bi1–N4–Bi2–N3 1.92.

Compound **2-Cl** forms a dimer with two edge-sharing octahedra and the amide groups in bridging coordination modes. This creates a central Bi_2_N_2_ ring with an N–Bi–N angle of 77.33(6)°, which is the main reason for deviations from ideally expected bond angles. The average Bi–Cl distance of 2.669 Å is in good agreement with previously reported Bi–Cl distances.^58–61^ In contrast to **2-Cl** with its apparent *C*_*i*_ symmetry, **2-Br** is a dinuclear species created by a face-sharing arrangement of an octahedron (around Bi1) and a pentagonal bipyramid (around Bi2) due to the varying number of pyridine ligands (Figure 1, top). However, the different coordination numbers of the bismuth atoms in **2-Br** only have a minor impact on Bi–N^amide^ and Bi–Br bond lengths, which remain similar for the two Bi centers or even identical within limits of error (average Bi–Br: 2.840 Å, in good agreement with previously reported Bi–Br distances^58–60^ ). It should be noted, that all three compounds **2-X** release bismuth-bound solvent molecules to form the monopyridine adduct [BiX_2_(NMe_2_)­py]_2_ (X = Cl, Br, I) upon exposure to fine vacuum for one hour.

Compounds **3-Cl** and **3-I** with only one halido ligand per bismuth atom are less Lewis acidic than compounds **2-X** (X = Cl–I), leading to a smaller coordination number of five for each bismuth atom (Figure 1, bottom). Both compounds may be described as *trans*-oriented edge-sharing square pyramids with amido groups in the bridging and in the apical positions. Compound **3-Cl** does not bear any neutral ligands, but each bismuth atom interacts with a chloride ligand of a neighboring formula unit, which creates a two-dimensional coordination polymer that extends along the crystallographic *a*/*b*-plane. Compound **3-I** crystallized as a pyridine-adduct (due to different crystallization conditions) and does not form a coordination polymer in the solid state. In both compounds, **3-Cl** and **3-I**, there is an asymmetry in the central Bi_2_N_2_ ring due to a thermodynamic *trans*-effect and the apical Bi–N bonds are shorter than the equatorial ones.

Compounds **2-Cl**, **2-Br**, **2-I**, **3-Cl** and **3-I** were further characterized by NMR spectroscopy, mass spectrometry, and elemental analysis. Compounds **2-X** (X = Cl, Br, I) are stable under argon atmosphere at room temperature for more than one month. All compounds are moderately soluble in pyridine, but only poorly soluble in solvents with a lower polarity. In pyridine-*d*_5_, the NMe_2_ groups of all compounds give rise to one broad singlet in the ^1^H NMR spectra (δ = 5.36 (**2-Cl**); 5.46 (**2-Br**); 5.74 (**2-I**); 4.20 (**3-Cl**) and 4.37 ppm (**3-I**)) and one singlet in the ^13^C NMR spectra (δ = 44.7 (**2-Cl**); 46.5 (**2-Br**); 52.0 (**2-I**); 44.1 (**3-Cl**) and 46.2 ppm (**3-I**)). In the case of compound **3-Cl**, low-temperature ^1^H NMR spectroscopy led to further signal broadening (300–243 K; fwhm_300 K_: 14.1 Hz, fwhm_243 K_: 36.9 Hz; Supp. Inf., Figure S10), suggesting dynamic exchange of the NMe_2_ groups. However, distinct resonances for the NMe_2_ groups in bridging and terminal positions could not be resolved at 243 K in pyridine-*d*_5_, which is in agreement with previous findings, that showed that these dynamics could only be frozen out at 213 K in THF-*d*_8_ for the related dicationic species [Bi_2_(μ_2_-NMe_2_)_2_(NMe_2_)­(thf)_6_]^2+^.^30^ The observed ^1^H NMR spectroscopic resonances of the NMe_2_ groups in **2-Cl**, **2-Br**, **2-I**, **3-Cl** and **3-I** are considerably downfield-shifted compared to Bi­(NMe_2_)_3_ (δ = 3.72 ppm (^1^H) in toluene-*d*_8_ at 295 K), which is ascribed to the electron-withdrawing character of the halide atoms and the bridging coordination mode of the amide groups (*vide supra*).^50^ In all cases the downfield-shift increases with the atomic number of the respective halogen atom. In contrast, the ^13^C NMR spectroscopic resonances of all compounds and Bi­(NMe_2_)_3_ (48.8 ppm, toluene-*d*_8_) appear in a relatively narrow range of 7.3 ppm.

With a series of well-defined mono-, di- and triamido bismuth compounds in hand (**2-X**, **3-X**, **1**), we set out to test their reactivity toward CS_2_. In all cases, reactions of these compounds with one equivalent of CS_2_ per amide ligand proceeded with the highly selective insertion of CS_2_ into the Bi–N bonds, furnishing compounds **4-X**, **5-X** and **6** in 80–96% isolated yield (Scheme 3). Compounds **4-I**, **5-Cl**, **5-Br**, **5-I** and **6** have previously been prepared by reaction of tetramethylthiuram disulfide with Bi halides (for **4-I**, **5-Cl**, **5-Br**, **5-I**) and reaction of tetramethylthiuram disulfide with bismuth metal or salt metathesis of a dimethyldithiocarbamate with bismuth halides (for **6**).^62–68^ The reaction of **1** with CS_2_ has previously been investigated, and the product was only identified by ^1^H NMR, IR, UV/vis spectroscopy and elemental analysis at the time;^33^ our analytical data obtained for these compounds is in agreement with and in some points extends the literature (*vide infra* and Supp. Inf.).^62–66^

**Scheme 3:**
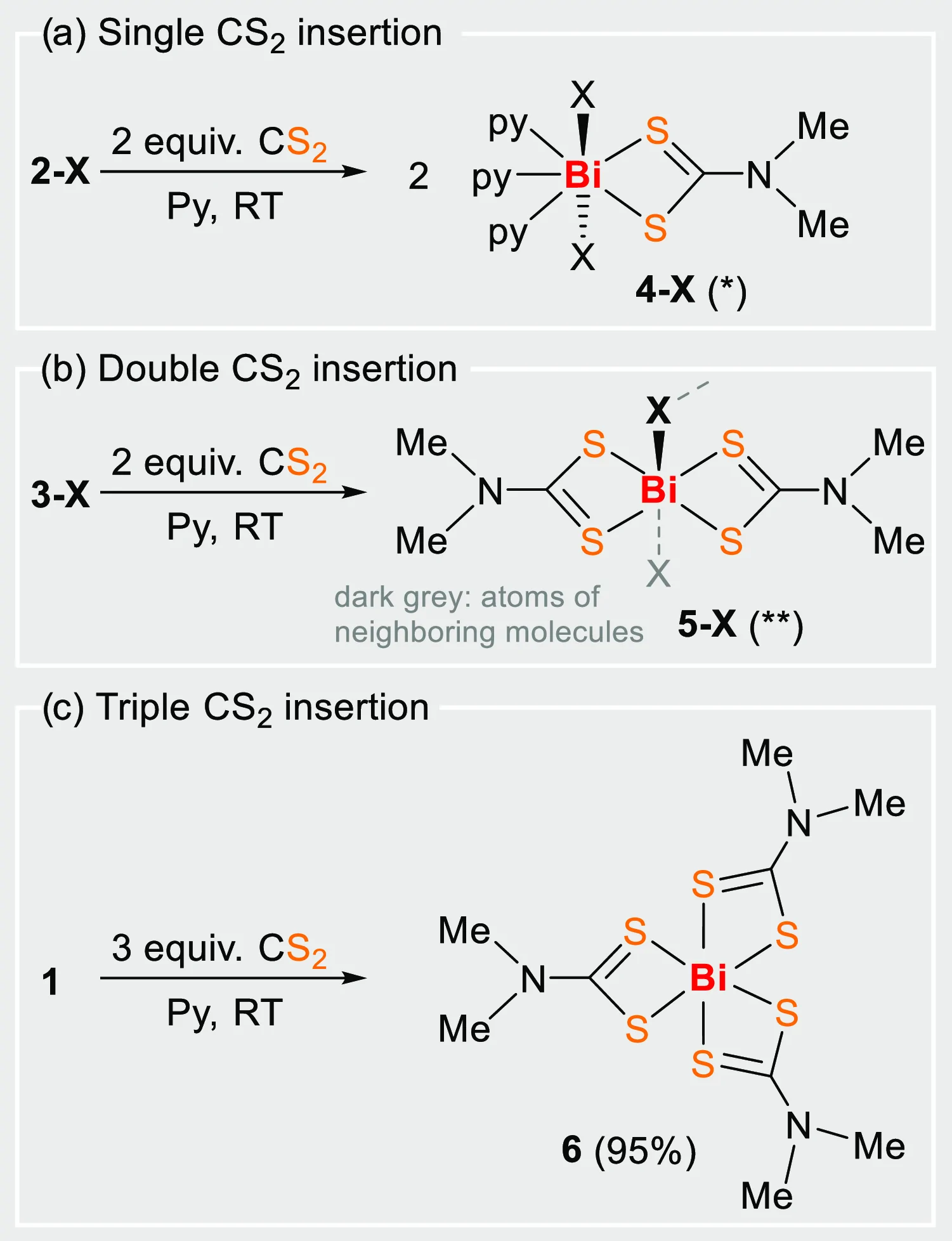
Selective insertion of CS_2_ into Bi–N bonds of (a) **2-X**, (b) **3-X**, and (c) **1**

a X = Cl, Br, I. Py: pyridine as solvent, py: pyridine as ligand. * Yield: 80% (X = Cl), 87% (X = Br), 83% (X = I). ** Yield: 88% (X = Cl), 91% (X = Br), 96% (X = I).

Compound **4-Cl** was obtained as a colorless crystalline solid, which did not show signs of degradation, when exposed to air or irradiated with UV light (LED, 365 nm). The ^1^H NMR spectrum of **4-Cl** shows a singlet for the NMe_2_ group at δ = 3.27 ppm (**4-Cl**, DCM-*d*_2_), similar to the chemical shifts observed for all compounds **4-X**, **5-X** and **6** (Supp. Inf.). In contrast to **5-X** and **6**, the coordination sphere of the compounds **4-X** is saturated by three pyridine ligands due to the presence of two electron-withdrawing halogenido ligands. As a result of their coordination to the Lewis-acidic central atom, their resonances are significantly downfield-shifted, when compared to noncoordinated pyridine (Δδ = 0.06–0.18 ppm).^69^ Qualitatively identical results were obtained from ^13^C NMR spectroscopic data (Supp. Inf.). While the pyridine ligands could not be abstracted by exposure of **4-X** to fine vacuum for several hours, they readily dissociate under mass-spectrometric conditions (*m*/*z* 398.91248, equivalent to [**4-Cl**–3Py]^+^). Compounds **4-Cl** and **4-I** were further analyzed by single-crystal X-ray diffraction studies, which revealed that in both molecular structures in the solid state, the central bismuth atom (Bi1) shows a coordination number of seven and adopts a distorted pentagonal bipyramidal geometry (Figure 2). The equatorial positions are occupied by three pyridine molecules and the bidentate dithiocarbamate ligand, while both halogenide ions occupy the axial positions. The Bi1–S1/S2 bond lengths show little variance (2.7451(16)–2.7622(17) Å for **4-Cl**; 2.7297(10)–2.7459(9) Å for **4-I**), indicating effective delocalization of electron density across the CS_2_ unit. This phenomenon is much more pronounced in **4-Cl** and **4-I** than in compound **5-Br** ^66^ and **6**, because a thermodynamic *trans*-effect cannot effectively be realized in **4-Cl** and **4-I**. The Bi–N (2.630(5)–2.782(5) Å) and Bi–Cl bond lengths (2.6703(16)–2.7078(17) Å) in **4-Cl** are slightly larger than in the structurally related complex cation [BiCl_2_(py)_5_]^+^ (Bi–Cl, 2.626(2)–2.666(2) Å; Bi–N, 2.539(7)–2.621(7) Å),^58^ mirroring the effective reduction of Lewis acidity at the bismuth center by coordination of the dithiocarbamate ligand.

**Figure 2:**
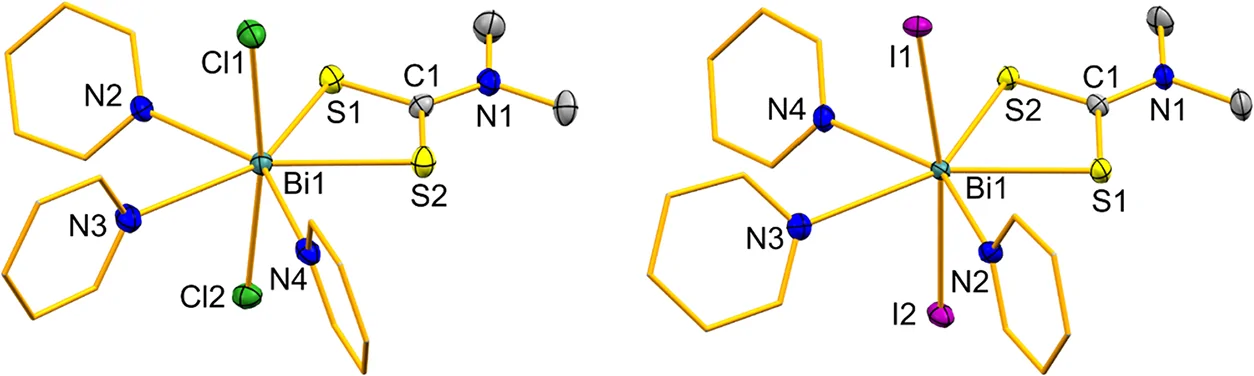
Molecular structure of **4-Cl** (left) and **4-I** (right) in the solid state. Displacement ellipsoids are shown at 50% probability, hydrogen atoms are omitted for clarity, carbon atoms of pyridine ligands are shown as wireframe. Selected bond distances [Å] and angles [deg]. Compound **4-Cl**: Bi1–Cl1 2.7078(17), Bi1–Cl2 2.6703(16), Bi1–S1 2.7622(17), Bi1–S2 2.7451(16), Bi1–N2 2.630(5), Bi1–N3 2.782(5), Bi1–N4 2.645(5), S1–C1 1.728(6), S2–C1 1.720(6), Cl2–Bi1–Cl1 170.53(5), S1–Bi1–Cl1 92.35(5), S1–Bi1–Cl2 94.53(5), S2–Bi1–Cl1 92.33(5), S2–Bi1–Cl2 96.45(5), S2–Bi1–S1 65.45(5). Compound **4-I**: Bi1–I1 3.0471(3), Bi1–I2 3.0401(3), Bi1–S1 2.7297(10), Bi1–S2 2.7459(9), Bi1–N2 2.671(3), Bi1–N3 2.803(3), Bi1–N4 2.664(3), S1–C1 1.717(4), S2–C1 1.717(4), I2–Bi1–I1 172.553(8), S1–Bi1–I1 90.12(2), S1–Bi1–I2 89.81(2), S2–Bi1–I1 90.12(2), S2–Bi1–I2 93.22(2), S2–Bi1–S1 65.28(3).

While cyclic alkyl­(amino)­carbenes (CAACs) have been very successful at forming adducts with a broad range of Lewis acids, this has been surprisingly challenging in the field of bismuth­(III) chemistry. Only a small number of such compounds is literature-known^70–73^ and unsuccessful attempts to obtain simple adduct have been reported.^73^ Recently, we have uncovered the net Bito-CAAC-transfer of halonium ions (“Cl^+^“, “Br^+^”) and nitrenium ions (“NMe_2_^+^”) via radical pathways as unexpected reactivity pathways after initial adduct formation between CAACs and BiX_3–n_(NMe_2_)_n_ (X = Cl, Br, n = 0, 2).^50^ We thus set out to investigate the influence of the dithiocarbamate ligand on this type of reaction. Addition of one equivalent of ^Me2^CAAC to **4-Cl** (as a representative bearing Cl^–^ and (Me_2_NCS_2_)^−^ ligands), **5-Br** (as a representative bearing Br^–^ and (Me_2_NCS_2_)^−^ ligands), and **6** (as a representative bearing only (Me_2_NCS_2_)^−^ ligands), was probed and in none of the cases, products of the above-mentioned redox reactions could be isolated. Instead, compound **4-Cl** underwent a ligand exchange reaction, with three pyridine ligands being exchanged for the sterically more demanding ^Me2^CAAC ligand to give compound **7** (Scheme 4a). ^1^H NMR spectroscopic analysis reveals no change in chemical shift of the singlet for the (Me_2_NCS_2_)^−^ moiety, when compared to the starting material **4-Cl**, suggesting that the ^Me2^CAAC ligand in **7** can fully compensate the donor capacity of the three pyridine ligands in **4-Cl**. Reliable ^13^C NMR spectroscopic data for **7** could not be obtained due to degradation in solution (Supp. Inf.). The connectivity of compound **7** could unambiguously be confirmed by single-crystal X-ray analysis (Supp. Inf.).

**Scheme 4:**
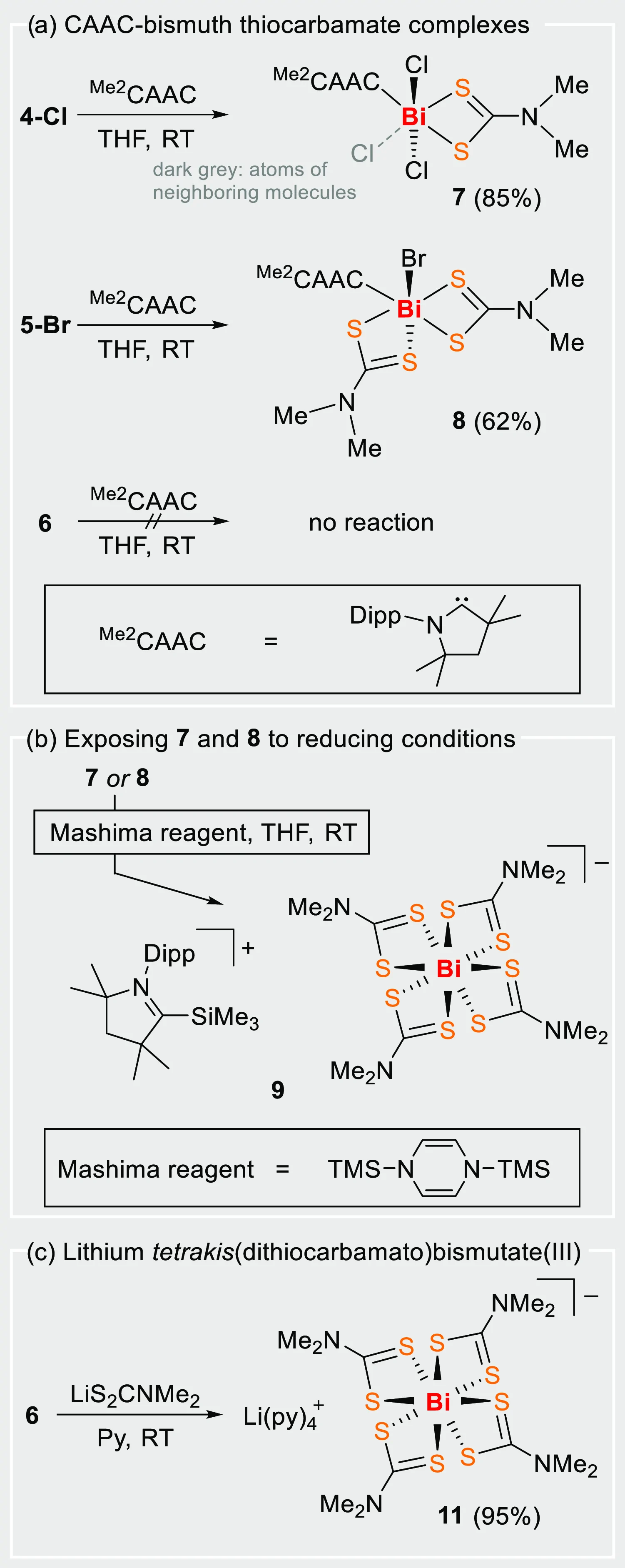
(a, b) Formation and Reactivity of Bismuth Dithiocarbamate CAAC Adducts; (c) Independent Synthesis of Dithiocarbamate-supported Bismuthate­(III) Complex

Reaction of [BiBr­(S_2_CNMe_2_)_2_]_∞_ (**5-Br**) with ^Me2^CAAC gave the adduct **8** (Scheme 4a). The efficacy of the synthesis was indicated by ^1^H NMR spectroscopy and HRMS analysis. The ^13^C NMR spectroscopic analysis was hampered by a combination of a rather poor solubility in moderately polar solvents and degradation in more polar solvents. Single-crystal X-ray analysis of compound **8** revealed its mononuclear nature in the solid state (Figure 3). This is the first example of a CAAC–[Bi­(III)] adduct yielding a well-defined mononuclear complex and might be responsible for its accelerated degradation in solution. The Bi–C^CAAC^ bond in **8** (2.593(4) Å) is the longest interaction of this type that has been reported to date, which is ascribed to the absence of sufficiently electronegative ligands in *trans* position to the ^Me2^CAAC ligand and a relatively high coordination number of six around the bismuth atom.

**Figure 3:**
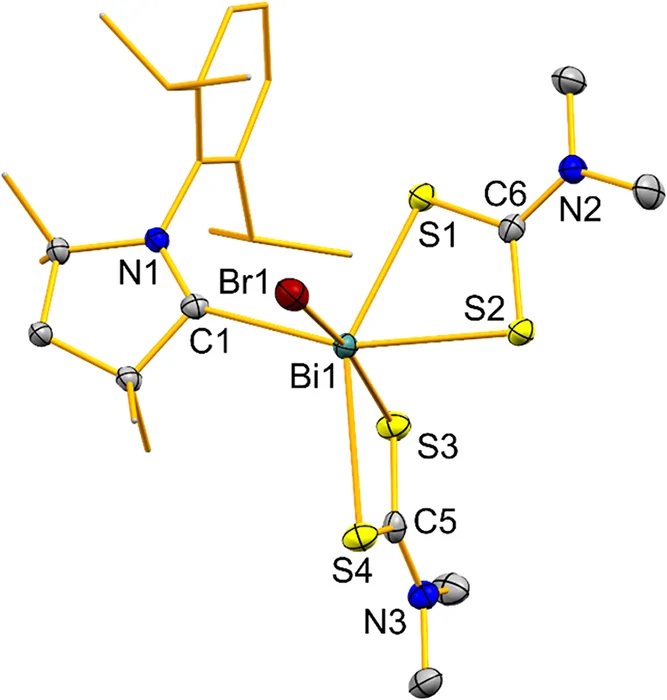
Molecular structure of **8** in the solid state. Ellipsoids are shown at 50% probability, hydrogen atoms are omitted for clarity, the carbene bound 2,6-di*iso*propylphenyl substituent is shown as wireframe. Selected bond distances [Å] and angles [deg]: Bi1–C1 2.593(4), Bi1–Br1 2.9845(5), Bi1–S1 2.6751(10), Bi1–S2 2.8269(10), Bi1–S3 2.7166(11), Bi1–S4 2.9458(10), C1–Bi1–Br1 92.25(8), C1–Bi1–S1 95.12(8), C1–Bi1–S2 161.04(8), C1–Bi1–S3, C1–Bi1–S4 108.31(8).

Specifically, the bismuth atom is found in a distorted octahedral coordination geometry with the ^Me2^CAAC and the Br ligand in a *cis* arrangement. As previously observed for **7**, also compound **8** experiences a considerable *trans*-influence. The Bi1–S2 bond *trans* to ^Me2^CAAC is rather long (2.8269(10) Å), leading to a short Bi1–S1 bond (2.6751(10) Å), as S1 and S2 are part of the same dithiocarbamate ligand. This induces a long Bi1–S4 bond (2.9458(10) Å) *trans* to S1 and a somewhat shorter Bi1–S3 bond (2.7166(11) Å) *trans* to Br1.

Both Bi–C^CAAC^ bonds in **7** (2.566(4) Å) and **8** (2.593(4) Å) are significantly longer than the interatomic distance predicted by the sum of the Pyykkö covalent radii of Bi and C (2.26 Å),^74^ but well below the sum of the van der Waals radii of Bi and C (3.77 Å),^75^ which is in agreement with the dative character of the C^CAAC^→Bi bonds in **7** and **8**.

The homoleptic bismuth compound **6** forms weak dinuclear aggregates in the solid state^63^ (also see Supp. Inf.), suggesting the potential to form adducts with sufficiently strong Lewis bases. However, from reactions of **6** with ^Me2^CAAC, no indications for effective interactions between the bismuth center and the ^Me^CAAC could be obtained, neither in solution nor in the solid state (Scheme 4a).

In view of the stabilizing effect of the dithiocarbamate ligand, the reduction of compounds **7**, **8**, and *in situ* generated [Bi­(CAAC)­(S_2_CNMe_2_)_2_I] with KC_8_, cobaltocene, and the Mashima reagent (1,4-dihydro-1,4-bis­(trimethylsilyl)-pyrazine) was attempted at temperatures down to −40 °C, aiming at the isolation of low-valent bismuth compounds (Scheme 4b). However, the reactions proceeded unselectively, and the unambiguous identification of specific products was challenging. Upon reduction of [Bi­(CAAC)­(S_2_CNMe_2_)_2_I] with CoCp_2_, [CoCp_2_]­[S_2_CNMe_2_] was identified by sc-XRD as a product, resulting from the oxidation of CoCp_2_ and subsequent ligand scrambling (Supp. Inf.). Only with the Mashima as a reducing agent could a bismuth-containing compound be identified. Specifically, small amounts of compound **9** were obtained, irrespective of the choice of bismuth compound (**7** or **8**) as the starting material. Compound **9** could not be obtained in amounts that are necessary for reliable spectroscopic and elemental analyses, but it was unambiguously identified by single-crystal X-ray analysis (Figure 4). This is the first example of a homoleptic ate complex with dithiocarbamate ligands in the chemistry of group 15 elements, complementing a previously reported related *tetrakis*(dithiocarbonato)­bismuthate complex.^76–78^ The bismuth atom in **9** is eight-coordinate with an irregular coordination polyhedron, which is (at least in part) due to the small bite angle of the dithiocarbamate ligand. The Bi–S bond lengths are found in the range of 2.8305(9)–2.9799(9) Å. This is on average significantly larger than the Bi–S bond lengths found in the dithiocarbamate complexes reported in this work, including homoleptic compound **6** (Bi–S: 2.601(2)–2.918(2) Å, see Supp. Inf.), and is due to the high coordination number and the overall negative charge of the bismuth complex ion in **9**. The cationic part in compound **9** is best described as a *C*-silylated iminium ion, which is unknown on the basis of the CAAC architecture.^79^ Suggested key steps for the formation of **9** are (i) the initial reduction of Bi­(III) to Bi­(II) along with the formation of Me_3_SiBr, (ii) disproportionation of Bi­(II) to Bi­(III) and Bi(0), (iii) reaction of the carbene with Me_3_SiBr to give [^Me2^CAAC–SiMe_3_]­Br, and (iv) ligand scrambling to give **9** and bromido bismuth compounds.

**Figure 4:**
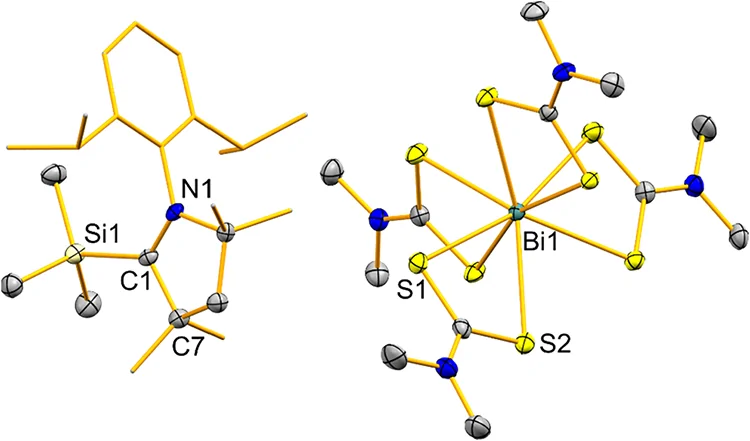
Molecular structure of **9** in the solid state. Ellipsoids are shown at 50% probability, hydrogen atoms are omitted for clarity, the 2,6-di*iso*propylphenyl substituent and part of the methyl substituents of the iminium cation are shown as wireframe. Selected bond distances [Å] and angles [deg]: Bi1–S1 2.8348(8), Bi1–S2 2.8965(9), Bi1–S3 2.8800(8), Bi1–S4 2.8305(9), Bi1–S5 2.8511(8), Bi1–S6 2.9242(7), Bi1–S7 2.8346(8), Bi1–S8 2.8963(9), C1–Si1 1.954(3), N1–C1 1.298(4), S1–Bi1–S2 62.51(2), S3–Bi1–S4 62.82(2), S5–Bi1–S6 62.01(2), S7–Bi1–S8 62.52(2).

In order to gain rational synthetic access to this unprecedented *tetrakis*(dithiocarbamato)­bismuthate­(III) motif, the lithiated carbamate LiS_2_CNMe_2_ (**10**) was synthesized from LiNMe_2_ and CS_2_ (see Supp. Inf.) and added to compound **6** (Scheme 4c). Indeed, the target compound [Li­(py)_4_]­[Bi­(S_2_CNMe_2_)_4_] (**11**) could be isolated in a near-quantitative yield of 95%. Compound **11** crystallized in the monoclinic space group *C*2/*c* (Figure 5). The molecular structure of the complex anion [Bi­(S_2_CNMe_2_)_4_]^−^ is highly similar to the one described within compound **9** (*vide supra*) with Bi–S distances ranging from 2.8361(7) to 2.9643(7) Å. Due to the additional saturation of the lithium cation with four equivalents of pyridine, no Li–S contacts are observed in the solid state. The expected tetrahedral coordination geometry of the lithium cation deviates with N–Li–N angles of 96.91(10) to 99.05(10)° most likely due to packing effects in the solid state, including weak CH^pyridin^ ^e^···S interactions (2.75–2.79 Å).

**Figure 5:**
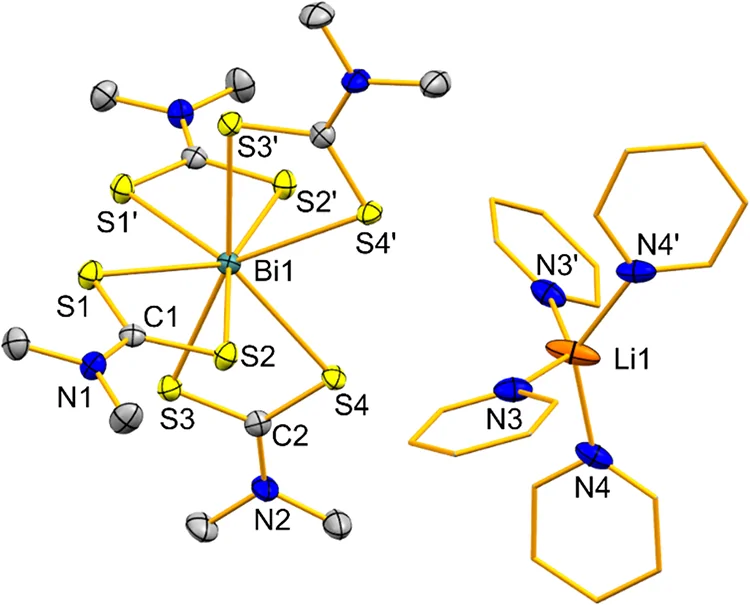
Molecular structure of **11** in the solid state. Ellipsoids are shown at 50% probability, hydrogen atoms are omitted for clarity, carbon atoms of pyridine ligands are shown as wireframe. Selected bond distances [Å] and angles [deg]: Bi1–S1 2.8701(7), Bi1–S2 2.8798(7), Bi1–S3 2.8361(7), Bi1–S4 2.9643(7), Bi1···Li1 6.387, Li1–N3 2.116(5), Li1–N4 2.089(5), S1–Bi1–S2 62.26(2), S3–Bi1–S4 61.61(2), N3–Li1–N4 99.05(10), N3–Li1–N4′ 96.91(10), N3–Li1–N3′ 136.5(6).

The ^1^H NMR spectrum of **11** shows one distinct singlet at 3.25 ppm (pyridine-*d*_5_), that is slightly downfield-shifted, when compared to that in the neutral species **6** (δ = 3.21 ppm, pyridine-*d*_5_). ^13^C NMR spectroscopic analyses additionally revealed a downfield-shift of the resonance of the quaternary carbon atom of the dithiocarbamate ligand from 204.1 ppm for the neutral species **6** to δ = 208.6 ppm for the complex anion [Bi­(S_2_CNMe_2_)_4_]^−^ that is part of **11**. The ^7^Li NMR spectrum of compound **11** shows one singlet at δ = 3.09 ppm (pyridine-*d*_5_).

Bismuth dithiocarbamates have been shown to bear potential for the synthesis of Bi_2_S_3_ particles.^80,43,44^ While more traditional approaches are based on solid-state synthesis, hydrothermal/solvothermal methods, and chemical bath deposition,^81^ bismuth dithiocarbamates can act as single-source precursors. This potentially avoids challenges such as high temperature/autoclave conditions, long reaction times, limited scalability, poor size/morphology control, and the need to control multiple parameters.^81^ Compound **6** represents the simplest homoleptic bismuth­(III) dithiocarbamate so far. It was thus identified as a promising precursor for the deposition of bismuth–sulfur-containing materials and tested in a proof-of-principle approach. The thermogravimetric analysis (TGA) of compound **6** is presented in Figure 6a. The decomposition of **6** occurred in a single step, with a mass loss of ca. 55% observed between ca. 280 and 330 °C, corresponding to a virtually quantitative formation of Bi_2_S_3_. This behavior differs from previously reported bismuth dithiocarbamates such as Bi­(S_2_CNCyEt)_3_ and Bi­(S_2_CN­(CH_2_)_5_)_3_, which show a two-step thermal degradation,^42,43^ Bi­(S_2_C­(THQ))_3_ (THQ = tetrahydroquinoline-*N*-yl), which undergoes decomposition in a large temperature window of ca. 100–300 °C (or even up to ca. 400 °C until mass loss corresponds to the formation of Bi_2_S_3_),^43^ and Bi­(S_2_CNEt_2_)_3_ which does not show a mass loss high enough to account for the quantitative formation of Bi_2_S_3_ under conditions of the thermogravimetric experiment.^44^ These differences can be ascribed to the simple substitution pattern in compound **6**, fostering a rapid, one-step thermal degradation process. After complete decomposition, a dark residue was obtained, characteristic of bismuth sulfide (Bi_2_S_3_) inorganic materials.^80,43^ The residue was further examined by powder X-ray diffraction analysis, confirming the formation and phase purity of Bi_2_S_3_ (Figure 6b).^82^ Identical results were obtained, when samples of **6** were heated to either 280 or 350 °C under an argon atmosphere in a sealed borosilicate ampule (heating rate: 5 °C/min, temperature maintained for 30 min after reaching the desired temperature, cooling by removal from heating source). The surface morphology and shape of the as-prepared Bi_2_S_3_ were examined by scanning electron microscopy (SEM). The samples prepared at 280 and 350 °C exhibit a uniform rod-like morphology (Figure 6c and Supp. Inf.). The elemental composition of the Bi_2_S_3_ samples synthesized at the two different reaction temperatures (280 and 350 °C) was examined using EDX analysis, confirming the stoichiometry of Bi_2_S_3_ (Supp. Inf.).

**Figure 6:**
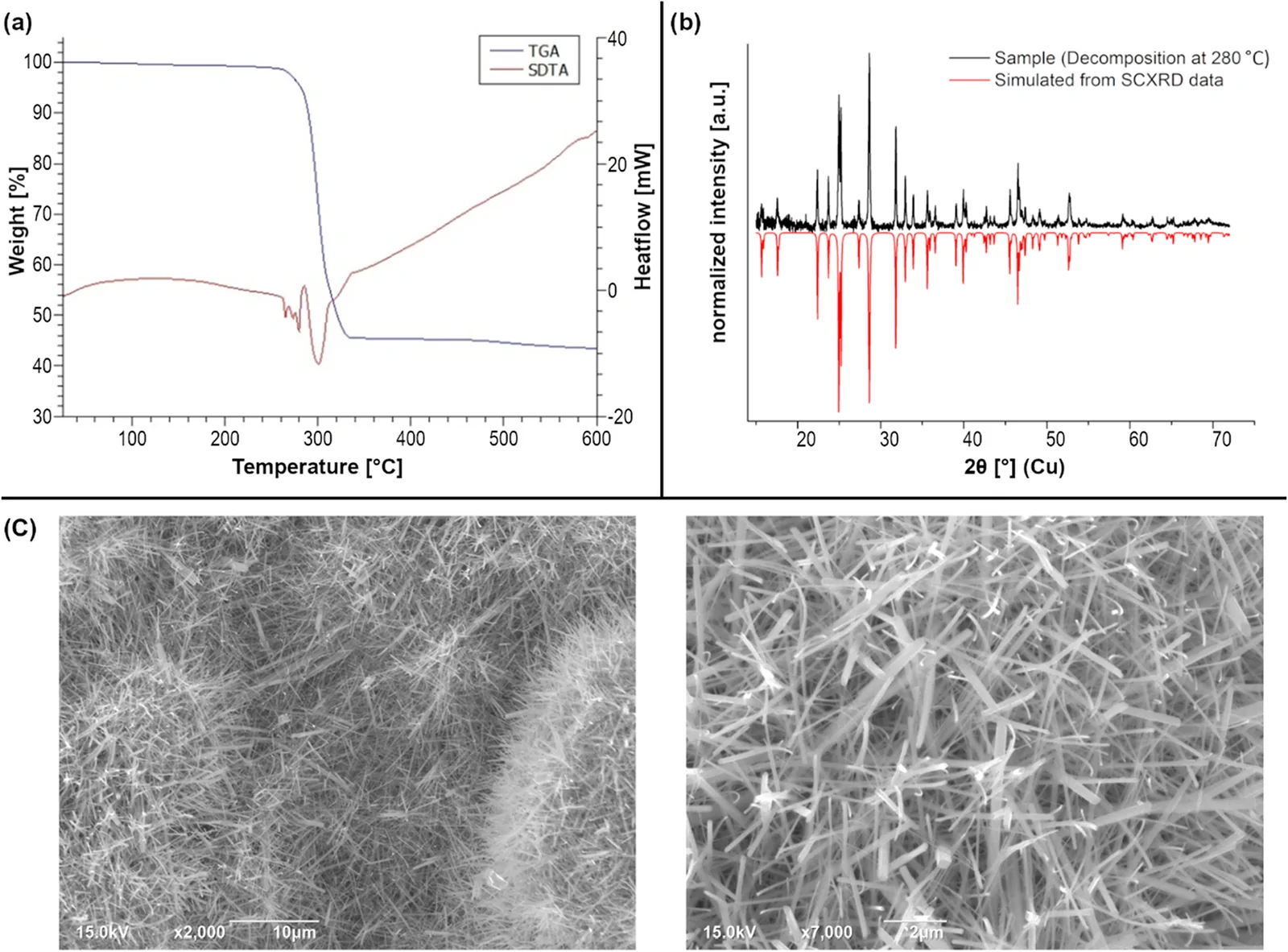
(a) Thermogravimetric and differential calorimetric analysis of **6** (heating rate: 5 °C/min; N_2_-flow rate: 30 mL/min). (b) p-XRD analysis of Bi_2_S_3_ prepared from **6** at 280 °C. (c) Scanning electron microscopy images of Bi_2_S_3_ prepared from **6** at 350 °C.

## Conclusion

In conclusion, we have presented a full series of halo­(bismuth)­amides [Bi­(NMe_2_)*_n_* X_3–*n*_] (X = Cl, Br, I; *n* = 1, 2). These compounds and the homoleptic species Bi­(NMe_2_)_3_ selectively insert one molecule of CS_2_ per Bi–N bond, resulting in straightforward single, double and triple CS_2_ insertion reactions. The resulting bismuth dithiocarbamates do not undergo selective redox reactions with cyclic­(alkyl)­amino carbenes (CAACs), but favor adduct formation or remain unreactive, which contrasts with previously reported bismuth amides. Under reducing conditions, a homoleptic *tetrakis*(dithiocarbamato)­bismuthate­(III) complex anion [Bi­(S_2_CNMe_2_)_4_]^−^ was structurally authenticated – an unprecedented structural motif, which was further made synthetically accessible *via* ligand transfer from LiS_2_CNMe_2_ to the homoleptic species Bi­(S_2_CNMe_2_)_3_. The neutral bismuth-*tris*(dithiocarbamate) Bi­(S_2_CNMe_2_)_3_ is a well-defined single source precursor for the generation of Bi_2_S_3_. These findings establish halo­(bismuth)­amides as valuable precursors for molecular bismuth compounds and underscore the ability of CS_2_ to be exploited as a valuable building block in bismuth-based coordination chemistry and materials science.

## Experimental Section

### General Considerations and Methods

All air- and moisture-sensitive manipulations were carried out under an inert atmosphere of purified argon (5.0) using standard Schlenk techniques or a glovebox (GS Systems; O_2_ ≤ 1 ppm, H_2_O ≤ 1 ppm). Solvents were dried over the appropriate drying agents, distilled, degassed and stored over 3 or 4 Å molecular sieves. BiCl_3_ was sublimed prior to use. Commercially available BiBr_3_ and BiI_3_ were used as received. CS_2_ was degassed by three freeze–pump–thaw cycles and subsequently stored over 3 Å molecular sieves. LiNMe_2_, Bi­(NMe_2_)_3_, Bi­(NMe_2_)_2_Br and ^Me2^CAAC were prepared according to literature protocols.^83,84,50,85^ No uncommon safety hazards are to be noted. NMR spectra were recorded on Bruker NEO300, AV500, or HD500 spectrometers. The chemical shift δ of the recorded ^1^H and ^13^C­{^1^H} NMR spectra is given relative to the residual solvent signal, which was used as a secondary standard and is defined by an external tetramethyl silane (TMS) standard. ^7^Li NMR spectra are referenced against a 1m solution of LiCl in H_2_O. Elemental analyses were performed using a vario MICRO Cube CHN­(S) analyzer (Elementar). LIFDI-MS spectra were recorded on a JEOL Accu TOF GCv time-of-flight mass spectrometer. An internal or external standard was used for drift time correction. The LIFDI ion source and FD-emitters were purchased from Linden ChroMasSpec GmbH. TGA-DSC measurements were performed on a STARe System TGA/DSC 3+ analyzer (Mettler Toledo) under N_2_-flow (30 mL/min). Powder X-ray diffraction data were recorded at 298 K on a StadiMP powder diffractometer (Stoe) in transmission geometry using Cu–Kα-radiation. Single-crystal X-ray diffraction data were recorded on Bruker D8 Quest or D8 Venture diffractometers equipped with Photon II or Photon III C14 detectors, respectively, using Mo–Kα-radiation (for a detailed description see Supp. Info). Energy-dispersive X-ray spectroscopy (EDX) was carried out on a Jeol JIB 4601F focused ion beam/secondary electron microscopy Dual System (FIB/SEM) operating at 30 kV. Elemental maps were acquired and the chemical composition was evaluated using the Bruker ESPRIT Software.

### Synthesis of [Bi­(NMe_2_)­Cl_2_py]_2_ (**2-Cl**)

BiCl_3_ (780 mg, 2.47 mmol, 2.0 equiv) and Bi­(NMe_2_)_3_ (**1**, 418 mg, 1.23 mmol, 1.0 equiv) were combined and suspended in pyridine (4 mL). The suspension was stirred for 30 min at room temperature under the exclusion of ambient light. Afterward all volatiles were removed *in vacuo*. The resulting pale-green solid was washed with ^*n*^pentane (2 × 5 mL) and subsequently dried *in vacuo*. Crystalline material suitable for single crystal X-ray analyses was obtained after storing a saturated solution of **2-Cl** in pyridine (2 mL) at −30 °C for 2 days. The compound crystallizes as bipyridine adduct [Bi­(NMe_2_)­Cl_2_py_2_]_2_ but can be desolvated to form the monopyridine adduct **2-Cl** by applying a vacuum (10^–3^ mbar) for 1 h. **Yield**: 1.40 g, 2.90 mmol, 94%. ^**1**^ **H NMR** (500 MHz, 298 K, Py-*d*_5_): δ = 5.36 (bs, 12H, N­(C*H*_3_)_2_) ppm. ^**13**^ **C­{** ^**1**^ **H} NMR** (126 MHz, 298 K, Py-*d*_5_): δ = 44.7 (bs, N­(*C*H_3_)_2_) ppm. **HR-MS** (LIFDI, pos.): Calc. for (^12^C_5_^1^H_5_^209^Bi_1_^35^Cl_2_^14^N_1_)^+^ (BiCl_2_(py)^+^): *m*/*z* = 357.96029, found *m*/*z* = 357.95996. **Elemental analysis** (%): Calc. for C_2_H_6_BiCl_2_N•(C_5_H_5_N) (403.06 g mol^–1^): C 20.86, H 2.75; N 6.95; found: C 20.85, H 2.70, N 6.56.

### Synthesis of [Bi­(NMe_2_)­Br_2_py]_2_ (**2-Br**)

BiBr_3_ (656 mg, 1.46 mmol, 2.0 equiv) and Bi­(NMe_2_)_3_ (**1**, 251 mg, 0.736 mmol, 1.0 equiv) were combined and suspended in pyridine (4 mL). The suspension was stirred for 30 min at room temperature under the exclusion of ambient light. Afterward all volatiles were removed *in vacuo*. The resulting pale-green solid was washed with ^*n*^pentane (2 × 5 mL) and subsequently dried *in vacuo*. Crystalline material suitable for single crystal X-ray analyses was obtained after storing a saturated solution of **2-Br** in pyridine (2 mL) at – 30 °C for 2 days. The compound crystallizes as bipyridine adduct [Bi­(NMe_2_)­Br_2_py_2_]_2_ but can be desolvated to form the monopyridine adduct **2-Br** by applying a vacuum (10^–3^ mbar) for 1 h. **Yield**: 1.03 g, 2.09 mmol, 95%. ^**1**^ **H NMR** (500 MHz, 298 K, Py-*d*_5_): δ = 5.46 (bs, 12H, N­(C*H*_3_)_2_) ppm. ^**13**^ **C­{** ^**1**^ **H} NMR** (126 MHz, 298 K, Py-*d*_5_): δ = 46.5 (bs, N­(*C*H_3_)_2_) ppm. **HR-MS** (LIFDI, pos.): Calc. for (^209^Bi_1_^79^Br_1_^81^Br_1_)^+^ ([**1** – NMe_2_)­(py)_2_]^+^: *m*/*z* = 368.81501, found *m*/*z* = 368.81827. **Elemental analysis** (%): Calc. for C_2_H_6_BiBr_2_N•(C_5_H_5_N)_1.1_ (499.88 g mol^–1^): C 18.02, H 2.32; N 5.88; found: C 17.97, H 2.62, N 5.82.

### Synthesis of [Bi­(NMe_2_)­I_2_py]_2_ (**2-I**)

BiI_3_ (400 mg, 0.668 mmol, 2.0 equiv) and Bi­(NMe_2_)_3_ (**1**, 116 mg, 0.340 mmol, 1.0 equiv) were combined and suspended in pyridine (3 mL). The suspension was stirred for 30 min at room temperature under the exclusion of ambient light. Afterward all volatiles were removed *in vacuo*. The resulting red solid was washed with *n-*pentane (3 × 5 mL) and subsequently dried *in vacuo*. **Yield**: 414 mg, 0.706 mmol, 70%. ^**1**^ **H NMR** (500 MHz, 298 K, Py-*d*_5_): δ = 5.74 (bs, 12H, N­(C*H*_3_)_2_) ppm. ^**13**^ **C­{** ^**1**^ **H} NMR** (126 MHz, 298 K, Py-*d*_5_): δ = 52.0 (bs, N­(*C*H_3_)_2_) ppm. **Elemental analysis** (%): Calc. for C_2_H_6_BiI_2_N·(C_5_H_5_N) (585.97 g mol^–1^): C 14.35, H 1.89, N 4.78; found: C 14.03, H 1.88, N 4.51.

### Synthesis of [Bi­(NMe_2_)_2_Cl]_∞_ (**3-Cl**)

BiCl_3_ (140 mg, 0.444 mmol, 1.0 equiv) and Bi­(NMe_2_)_3_ (**1**, 303 mg, 0.888 mmol, 2.0 equiv) were combined and suspended in pyridine (3 mL). The suspension was stirred for 30 min at room temperature under the exclusion of ambient light. Afterward all volatiles were removed *in vacuo*. The resulting pale yellow solid was washed with *n-*pentane (2 × 5 mL) and subsequently dried *in vacuo*. Crystalline material suitable for single crystal X-ray analyses was obtained after layering a saturated solution (2 mL) of **3-Cl** in pyridine with hexane (0.5 mL) and stroring at – 30 °C for 2 days. **Yield**: 408 mg, 1.23 mmol, 92%. ^**1**^ **H NMR** (500 MHz, 298 K, Py-*d*_5_): δ = 4.20 (bs, 12H, N­(C*H*_3_)_2_) ppm. ^**13**^ **C­{** ^**1**^ **H} NMR** (126 MHz, 298 K, Py-*d*_5_): δ = 44.1 (s, N­(*C*H_3_)_2_) ppm. **HR-MS** (LIFDI, pos.): Calc. for (^12^C_4_^1^H_12_^209^Bi_1_^35^Cl_1_^14^N_2_)^+^ ([**3-Cl**]^•+^): *m*/*z* = 332.04928, found *m*/*z* = 332.04888. **Elemental analysis** (%): Calc. for C_4_H_12_BiClN_2_ (332.58 g mol^–1^): C 14.45, H 3.64, N 8.42; found: C 14.68, H 3.39, N 8.06.

### Synthesis of [Bi­(NMe_2_)_2_I]_∞_ (**3-I**)

BiI_3_ (218 mg, 0.370 mmol, 1.0 equiv) and Bi­(NMe_2_)_3_ (**1**, 253 mg, 0.742 mmol, 2.0 equiv) were combined and suspended in pyridine (3 mL). The suspension was stirred for 30 min at room temperature under the exclusion of ambient light. Afterward all volatiles were removed *in vacuo*. The resulting orange solid was washed with ^*n*^pentane (2 × 5 mL) and subsequently dried *in vacuo*. Crystalline material suitable for single crystal X-ray analyses was obtained after storing a saturated solution of **3-I** in pyridine (1 mL) at −35 °C for 1 day. From pyridine, the compound crystallizes as monopyridine adduct [Bi­(NMe_2_)_2_Ipy]_2_ but can be completely desolvated by applying a vacuum (10^–3^ mbar) for 1 h. **Yield**: 428 mg, 1.01 mmol, 91%. ^**1**^ **H NMR** (500 MHz, 298 K, Py-*d*_5_): δ = 4.37 (bs, 12 H, N­(C*H*_3_)_2_) ppm. ^**13**^ **C­{** ^**1**^ **H} NMR** (126 MHz, 298 K, Py-*d*_5_): δ = 46.2 (s, N­(*C*H_3_)_2_) ppm. **Elemental analysis** (%): Calc. for C_4_H_12_BiIN_2_ (424.04 g mol^–1^): C 11.33, H 2.85, N 6.61; found: C 11.38, H 2.78, N 6.38.

### Synthesis of Bi­(CS_2_NMe_2_)­Cl_2_py_3_ (**4-Cl**)

CS_2_ (79 μL, 99 mg, 1.30 mmol, 2.1 equiv) was added to a stirred solution of [Bi­(NMe_2_)­Cl_2_py]_2_ (**2-Cl**, 500 mg, 0.620 mmol, 1.0 equiv) in pyridine (5 mL). The mixture was stirred for 1 h at room temperature and subsequently all volatiles were removed *in vacuo*. The resulting colorless solid was washed with *n-*pentane (2 × 5 mL) and the product was dried *in vacuo*. Crystalline material suitable for single crystal X-ray analyses was obtained after storing a saturated solution of **4-Cl** in pyridine (2 mL) at – 30 °C for tow days. *Note*: The degree of solvation lies between two and three equivalents of pyridine and has to be determined individually for each batch. **Yield**: 597 mg, 0.986 mmol, 80%. ^**1**^ **H NMR** (500 MHz, 298 K, CD_2_Cl_2_): δ = 3.27 (s, 6H, N­(C*H*_3_)_2_), 7.34 (m, *m*-py), 7.75 (m, *p*-py), 8.77 (m, *o*-py) ppm. ^**13**^ **C­{** ^**1**^ **H} NMR** (126 MHz, 298 K, CD_2_Cl_2_): δ = 44.6 (s, N­(*C*H_3_)_2_), 124.8 (s, *m*-py), 137.3 (s, *p*-py), 150.3 (s, *o*-py), 197.8 (*C*S_2_) ppm. **HR-MS** (LIFDI, pos.): Calc. for (^12^C_3_^1^H_6_^209^Bi_1_^35^Cl_2_^14^N_1_^32^S_2_)^+^ ([**4-Cl** – 3 py]^+^): *m*/*z* = 398.91225, found *m*/*z* = 398.91248. **Elemental analysis** (%): Calc. for C_3_H_6_BiCl_2_NS_2_·(C_5_H_5_N)_2.6_ (605.75 g mol^–1^): C 31.73, H 3.16, N 8.32; found: C 31.56, H 3.25, N 8.69.

### Synthesis of Bi­(CS_2_NMe_2_)­Br_2_py_3_ (**4-Br**)

CS_2_ (36 μL, 46 mg, 0.598 mmol, 2.1 equiv) was added to a stirred solution of [Bi­(NMe_2_)­Br_2_py]_2_ (**2-Br**, 280 mg, 0.285 mmol, 1.0 equiv) in pyridine (5 mL). The mixture was stirred for 1 h at room temperature and subsequently all volatiles were removed *in vacuo*. The resulting colorless solid was washed with *n*-pentane (2 × 5 mL) and the product was dried *in vacuo*. *Note*: The degree of solvation lies between two and three equivalents of pyridine and must be determined individually for each batch. **Yield:** 360 mg, 0.496 mmol, 87%. ^**1**^ **H NMR** (500 MHz, 298 K, Py-*d*_5_): δ = 2.94 (6H, N­(C*H*_3_)_2_) ppm. ^**13**^ **C­{** ^**1**^ **H} NMR** (126 MHz, 298 K, Py-*d*_5_): δ = 43.1 (s, N­(*C*H_3_)_2_), 200.3 (s, *C*S_2_) ppm. **Elemental analysis** (%), calc. for C_3_H_6_BiBr_2_NS_2_·(C_5_H_5_N)_3.1_ (734.21 g mol^–1^): C 30.26, H 2.95, N 7.82; found: C 30.21, H 2.90, N 7.68.

### Synthesis of Bi­(CS_2_NMe_2_)­I_2_py_3_ (**4-I**)

CS_2_ (13 μL, 16 mg, 0.210 mmol, 2.1 equiv) was added to a stirred solution of [Bi­(NMe_2_)­I_2_py]_2_ (**2-I**, 0.117 mg, 0.100 mmol, 1.0 equiv) in pyridine (5 mL). The mixture was stirred for 1 h at room temperature and subsequently all volatiles were removed *in vacuo*. The resulting yellow solid was washed with *n-*pentane (2 × 5 mL) and the product was dried *in vacuo*. Crystalline material suitable for single crystal X-ray analyses was obtained in a gas-diffusion setup from a saturated solution of **4-I** in pyridine (0.5 mL) and ^*n*^hexane (1 mL) at room temperature. *Note*: The degree of solvation lies between two and three equivalents of pyridine and has to be determined individually for each batch. **Yield:** 136 mg, 0.166 mmol, 83%. ^**1**^ **H NMR** (500 MHz, 298 K, Py-*d*_5_): δ = 2.93 (6H, N­(C*H*_3_)_2_) ppm. ^**13**^ **C­{** ^**1**^ **H} NMR** (126 MHz, 298 K, Py-*d*_5_): δ = 42.7 (s, N­(*C*H_3_)_2_), 199.9 (s, *C*S_2_) ppm. **Elemental analysis** (%): Calc. for C_3_H_6_BiI_2_NS_2_·(C_5_H_5_N)_3_ (820.30 g mol^–1^): C 26.36, H 2.58, N 6.83; found: C 26.14, H 2.58, N 6.62.

### Synthesis of [Bi­(CS_2_NMe_2_)_2_Cl]_∞_ (**5-Cl**)

CS_2_ (191 μL, 240 mg, 3.16 mmol, 2.1 equiv) was added to a stirred solution of Bi­(NMe_2_)_2_Cl (**3-Cl**, 500 mg, 1.53 mmol, 1.0 equiv) in pyridine (5 mL). The mixture was stirred for 1 h at room temperature and subsequently all volatiles were removed *in vacuo*. The resulting yellow solid was washed with *n-*pentane (2 × 5 mL) and the product was dried *in vacuo*. *Note*: The amount of residual solvent may vary and must be determined individually for each batch. **Yield**: 650 mg, 1.34 mmol, 88%. ^**1**^ **H NMR** (500 MHz, 298 K, Py-*d*_5_): δ = 3.13 (s, 12H, N­(C*H*_3_)_2_) ppm. ^**13**^ **C­{** ^**1**^ **H} NMR** (126 MHz, 298 K, Py-*d*_5_): δ = 44.0 (s, N­(*C*H_3_)_2_), 203.2 (s, *C*S_2_) ppm. **HR-MS** (LIFDI, pos.): Calc. for (^12^C_6_^1^H_12_^209^Bi_1_^14^N_2_^32^S_4_)^+^ ([**5-Cl** – Cl]^+^): *m*/*z* = 448.9682, found *m*/*z* = 448.9680. **Elemental analysis** (%), calc. for C_6_H_12_BiClN_2_S_4_ (484.85 g mol^–1^): C 14.86, H 2.49, N 5.78; found: C 15.01, H 2.33, N 5.46.

### Synthesis of [Bi­(CS_2_NMe_2_)_2_Br]_∞_ (**5-Br**)

CS_2_ (168 μL, 212 mg, 2.78 mmol, 2.1 equiv) was added to a stirred solution of Bi­(NMe_2_)_2_Br (**3-Br**, 500 mg, 1.33 mmol, 1.0 equiv) in pyridine (5 mL). The mixture was stirred for 1 h at room temperature and subsequently all volatiles were removed *in vacuo*. The resulting yellow solid was washed with *n-*pentane (2 × 5 mL) and the product was dried *in vacuo*. *Note*: The amount of residual solvent may vary and must be determined individually for each batch. **Yield:** 640 mg, 1.21 mmol, 91%. ^**1**^ **H NMR** (500 MHz, 298 K, Py-*d*_5_): δ = 3.11 (s, 12H, N­(C*H*_3_)_2_) ppm. ^**13**^ **C­{** ^**1**^ **H} NMR** (126 MHz, 298 K, Py-*d*_5_): δ = 43.4 (s, N­(*C*H_3_)_2_), 202.4 (s, *C*S_2_) ppm. **Elemental analysis** (%): Calc. for C_6_H_12_BiBrN_2_S_4_·(C_5_H_5_N)_0.4_ (560.94 g mol^–1^): C 17.13, H 2.52, N 5.99; found: C 17.24, H 2.81, N 5.98.

### Synthesis of [Bi­(CS_2_NMe_2_)_2_I]_∞_ (**5-I**)

CS_2_ (59 μL, 74 mg, 0.969 mmol, 3.0 equiv) was added to a stirred solution of Bi­(NMe_2_)_2_I (**3-I**, 137 mg, 0.323 mmol, 1.0 equiv) in pyridine (3 mL). The mixture was stirred for 30 min at room temperature and subsequently all volatiles were removed *in vacuo*. The resulting green solid was washed with *n-*pentane (2 × 5 mL) and the product was dried *in vacuo*. *Note*: The amount of residual solvent may vary and must be determined individually for each batch. **Yield**: 179 mg, 3.11 mmol, 96%. ^**1**^ **H NMR** (500 MHz, 298 K, Py-*d*_5_): δ = 3.13 (s, 12H, N­(C*H*_3_)_2_) ppm. ^**13**^ **C­{** ^**1**^ **H} NMR** (126 MHz, 298 K, Py-*d*_5_): δ = 43.7 (s, N­(*C*H_3_)_2_), 202.8 (s, *C*S_2_) ppm. **Elemental analysis** (%), calc. for C_6_H_12_BiIN_2_S_4_·(C_5_H_5_N)_0.7_ (631.67 g mol^–1^): C 18.06, H 2.47, N 5.99; found: C 18.21, H 2.57, N 5.80.

### Synthesis of [Bi­(CS_2_NMe_2_)_3_]_2_ (**6**)

CS_2_ (266 μL, 336 mg, 4.41 mmol, 3.0 equiv) was added to a stirred solution of Bi­(NMe_2_)_3_ (**1**, 500 mg, 1.47 mmol, 1.0 equiv) in THF (4 mL). The mixture was stirred for 1 h at room temperature and subsequently all volatiles were removed *in vacuo*. The resulting yellow solid was washed with *n-*pentane (2 × 5 mL) and the product was dried *in vacuo*. Crystalline material suitable for single crystal X-ray analyses was obtained after storing a saturated solution of **6** in THF (2 mL) at room temperature for 1 day. **Yield:** 800 mg, 1.40 mmol, 95%. ^**1**^ **H NMR** (500 MHz, 298 K, CD_2_Cl_2_): δ = 3.39 (s, 18H, N­(C*H*_3_)_2_) ppm. ^**1**^ **H NMR** (300 MHz, 298 K, Py-*d*_5_): δ = 3.21 (s, 18H, N­(C*H*_3_)_2_) ppm. ^**13**^ **C­{** ^**1**^ **H} NMR** (126 MHz, 298 K, CD_2_Cl_2_): δ = 44.6 (s, N­(*C*H_3_)_2_), 202.2 (s, *C*S_2_) ppm. ^**13**^ **C­{** ^**1**^ **H} NMR** (75 MHz, 298 K, Py-*d*_5_): δ = 43.9 (s, N­(*C*H_3_)_2_), 204.1 (s, *C*S_2_) ppm. **HR-MS** (LIFDI, pos.): Calc. for (^12^C_6_^1^H_12_^209^Bi_1_^14^N_2_^32^S_4_)^+^ ([**6** – (S_2_CNMe_2_)]^+^): *m*/*z* = 448.96871, found *m*/*z* = 448.96635.

### Synthesis of [^Me2^CAAC–Bi­(CS_2_NMe_2_)­Cl_2_] (**7**)

Bi­(S_2_CNMe_2_)­Cl_2_py_3_ (**4-Cl**, 340 mg, 0.533 mmol, 1.0 equiv) and ^Me2^CAAC (152 mg, 0.533 mmol, 1.0 equiv) were taken into a vial and THF (6 mL) was added at room temperature. The color of the reaction mixture immediately changed from colorless to dark green, and subsequently the mixture was kept for crystallization at −30 °C. Crystalline material suitable for single crystal X-ray analyses was obtained after storing the solution for 2 days at −30 °C. Following, the solution was carefully decanted and the crystalline material was isolated. **Yield**: 310 mg, 0.452 mmol, 85%. ^**1**^ **H NMR** (500 MHz, 298 K, THF-*d*_8_): δ = 1.25 (d, ^3^ *J* = 6.6 Hz, 6H, CH­(C*H*_3_)_2_), 1.31 (bs, 6H, CH­(C*H*_3_)_2_), 1.44 (s, 6H, NCC­(C*H*_3_)_2_), 1.69 (s, 6 H, NC­(C*H*_3_)_2_), 2.04 (s, 2H, CC*H*_2_N), 3.02 (sept, ^3^ *J* = 6.5 Hz, 2H, C*H*(C*H*_3_)_2_), 3.18 (s, 6H, N­(C*H*_3_)_2_) 7.25 (d, ^3^ *J* = 7.6 Hz, 2H, Dipp-*m-H*), 7.33 (t, ^3^ *J* = 7.6 Hz, 1H, Dipp*-p-H*) ppm. ^**1**^ **H NMR** (500 MHz, 298 K, CD_2_Cl_2_): δ = 1.31 (d, ^3^ *J* = 6.5 Hz, 6H, CH­(C*H*_3_)_2_), 1.37 (d, ^3^ *J =* 6.4 Hz, 6H, CH­(C*H*_3_)_2_), 1.50 (s, 6H, NCC­(C*H*_3_)_2_), 1.83 (s, 6H, NC­(C*H*_3_)_2_), 2.17 (s, 2H, CC*H*_2_N), 2.91 (sept, ^3^ *J* = 6.5 Hz, 2H, C*H*(C*H*_3_)_2_), 3.27 (s, 6H, N­(C*H*_3_)_2_) 7.35 (d, ^3^ *J* = 7.9 Hz, 2H, Dipp-*m-H*), 7.49 (t, ^3^ *J* = 8.0 Hz 1H, Dipp*-p-H*) ppm. ^**13**^ **C­{** ^**1**^ **H} NMR** (126 MHz, 298 K, CD_2_Cl_2_): δ = 25.8, 26.2, 29.4, 29.6, 30.3, 44.5, 68.3, 126.9, 131.4, 146.5, 184.8 ppm. *Note:* Due to the low solubility of **7** in both CD_2_Cl_2_ and THF-*d*_8_, a satisfactory ^13^C NMR spectrum could not be obtained after several tries. Although the resonances are identifiable in the ^1^H NMR spectra, the compound easily starts degrading. Hence the ^13^C NMR spectra in CD_2_Cl_2_ shows a mixture of products. Compound **7** degrades even faster in pyridine-*d*_5_ and bismuth black precipitates from the solution. **HR-MS** (LIFDI, pos.): Calc. for (^12^C_3_^1^H_6_^209^Bi_1_^35^Cl_1_^14^N_1_^32^S_2_) ([**7** – (^Me2^CAAC)­(Cl)]^+^): *m*/*z* = 363.94340, found *m*/*z* = 363.94406; calc. for (^12^C_20_^1^H_32_^14^N_1_)^+^ ([^Me2^CAAC]^+^): *m*/*z* = 286.25347, found *m*/*z* = 286.25446; calc. for (^12^C_3_^1^H_6_^209^Bi_1_^32^S_2_^14^N_1_)^+^ ([**7** – (^Me2^CAAC)­(Cl_2_)]^+^): *m*/*z* = 328.9740, found *m*/*z* = 328.9735.

### Synthesis of [^Me2^CAAC–Bi­(CS_2_NMe_2_)_2_Br] (**8**)

Bi­(CS_2_NMe_2_)_2_Br (**5-Br**, 370 mg, 699 mmol, 1.0 equiv) and ^Me2^CAAC (0.200 mg, 701 mmol, 1.0 equiv) were taken in a vial and THF (10 mL) was added at room temperature. The color of the reaction mixture immediately changed from colorless to dark green and the solution was stirred for 1 h at room temperature. Subsequently the mixture was filtered and the solution was kept for crystallization at – 30 °C. Crystalline material suitable for single crystal X-ray analyses was obtained after storing the solution for 2 days at – 30 °C. Following, the solution was carefully decanted and the crystalline material was isolated. **Yield**: 337 mg, 0.414 mmol, 62%. ^**1**^ **H NMR** (500 MHz, 298 K, THF-*d*_8_): δ = 1.24–1.27 (m, 2 × 6H, CH­(C*H*_3_)_2_), 1.36 (bs, 6H, CH­(C*H*_3_)_2_), 1.36 (s, 6H, NCC­(C*H*_3_)_2_), 1.68 (s, 6H, NC­(C*H*_3_)_2_), 1.78 (s, 2H, CC*H*_2_N), 3.01 (sept, 2H, C*H*(C*H*_3_)_2_), 3.26 (s, 6H, N­(C*H*_3_)_2_) 7.17 (d, ^3^ *J* = 7.6 Hz, 2H, Dipp-*m-H*), 7.24 (t, ^3^ *J* = 7.5 Hz 1H, Dipp*-p-H*) ppm. ^**13**^ **C­{** ^**1**^ **H} NMR** (126 MHz, 298 K, CDCl_3_): δ = 22.71, 26.6, 27.2, 28.4, 30.0, 32.8, 43.6, 51.5, 62.2, 83.8, 102.2, 124.7, 125.4, 127.7, 128.9, 142.0, 146.9, 149.6, 153.2, 204.4 ppm. *Note:* Due to the low solubility of **8** in THF-*d*_8_, a satisfactory ^13^C NMR spectrum could not be obtained after several tries. Although the resonances are identifiable in the ^1^H NMR spectra, the compound easily starts degrading. **HR-MS** (LIFDI, pos.): Calc. for (^12^C_6_^1^H_12_^209^Bi_1_^14^N_2_^32^S_4_) ([**8**–(CAAC)­(Br)]^+^): *m*/*z* = 448.9682, found *m*/*z* = 448.9680. **Elemental analysis** (%), calc. for C_26_H_43_BiBrN_3_S_4_•(C_4_H_8_O)_1.4_ (915.73 g mol^–1^): C 41.45, H 5.97, N 4.59; found: C 41.34, H 6.08, N 4.86.

### Synthesis of [^Me2^CAAC–SiMe_3_]­[ Bi­(CS_2_NMe_2_)_4_] (**9**)

A solution of 1,4-dihydro-1,4-bis­(trimethylsilyl)-pyrazine (Mashima’s reagent, 16.5 mg, 0.07 mmol, 1 equiv) in THF (1 mL) was added dropwise at room temperature to solutions of **7** (50 mg, 0.07 mmol, 1 equiv) or **8** (57 mg, 0.07 mmol, 1 equiv) in THF (5 mL). During the additions a dark solid precipitated and was subsequently filtered off. Crystalline material suitable for single crystal X-ray analyses was obtained after storing the solutions at – 30 °C for three to 4 days. However, only few crystals of compound **10** were obtained and the compound could not be isolated in amounts necessary for reliable spectroscopic analyes, elemental analyses or the calculation of a yield.

### Synthesis of Li­[S_2_CNMe_2_] (**10**)

CS_2_ (0.146 mL, 2.41 mmol, 1.5 equiv) was added to a stirred solution of LiNMe_2_ (82 mg, 1.61 mmol, 1.0 equiv) in THF (3 mL) at room temperature, which resulted in an immediate change of color from clear-colorless to clear-yellow and heat formation. The reaction mixture was stirred for 10 min at room temperature and subsequently all volatiles were removed *in vacuo*. After washing with *n-*pentane (2 × 10 mL) and subsequent drying *in vacuo*, Li­[S_2_CNMe_2_] (**10**) was afforded as an off-white powder. *Note*: Even after thorough drying, a small amount of THF remains in the product, which is consistent with the results of NMR spectroscopy and elemental analyses. **Yield:** 191 mg, 1.48 mmol, 92%. ^**1**^ **H NMR** (300 MHz, 298 K, Py-*d*_5_): δ = 3.70 (s, 6H, N­(C*H*_3_)_2_) ppm. ^**13**^ **C­{** ^**1**^ **H} NMR** (75 MHz, 298 K, Py-*d*_5_): δ = 44.0 (s, N­(*C*H_3_)_2_), 214.2 (s, *C*S_2_) ppm. ^**7**^ **Li NMR** (117 MHz, 298 K, Py-*d*_5_): δ = 3.15 (s) ppm. **Elemental analysis** (%): Calc. for C_3_H_6_LiNS_2_·(C_4_H_8_O)_0.02_ (128.6 g mol^–1^): C 28.77, H 4.83, N 10.89; found: C 28.70, H 4.69, N 10.84.

### Synthesis of [Li­(py)_4_]­[Bi­(S_2_CNMe_2_)_4_] (**11**)

Li­[S_2_CNMe_2_] (**10**, 15 mg, 0.118 mmol, 1.0 equiv) and Bi­(S_2_CNMe_2_)_3_ (**6**, 67 mg, 0.118 mmol, 1.0 equiv) were combined and dissolved in pyridine (2 mL). The resulting clear-yellow solution was stirred for 5 min at room temperature, in which the color of the solution darkened. Subsequently all volatiles were removed *in vacuo*. After washing with *n-*pentane (2 × 10 mL) and subsequent drying *in vacuo*, [Li­(py)_4_]­[Bi­(S_2_CNMe_2_)_4_] (**11**) was afforded as pale-green powder. **Yield:** 113 mg, 0.112 mmol, 95%. ^**1**^ **H NMR** (500 MHz, 298 K, Py-*d*_5_): δ = 3.25 (s, 24H, N­(C*H*_3_)_2_) ppm. ^**13**^ **C­{** ^**1**^ **H} NMR** (125 MHz, 298 K, Py-*d*_5_): δ = 43.8 (s, N­(*C*H_3_)_2_), 208.6 (s, *C*S_2_) ppm. ^**7**^ **Li NMR** (194 MHz, 298 K, Py-*d*_5_): δ = 3.09 (s) ppm. **Elemental analysis** (%): Calc. for C_12_H_24_BiLiN_4_S_8_·(C_5_H_5_N)_4_ (1013.2 g mol^–1^): C 37.94, H 4.38, N 11.06, S 25.31; found: C 38.00, H 4.24, N 11.06, S 25.57.

## Supplementary Material



## Notes

Crystallographic details, plots of NMR, HRMS, EDX, and UV/vis spectra, and powder diffractograms (PDF)
