# Supplementary material for: Insertion of CS2 into Bi–N Bonds: Coordination Chemistry and Reactivity of Bismuth Dithiocarbamates
Source: Inorg Chem. 2026 Sep 30;65(40):23664–76. doi: 10.1021/acs.inorgchem.6c03443 (PMC13664088; doi:10.1021/acs.inorgchem.6c03443)
Supplement: Supplementary file 1 [file ic6c03443_si_001.pdf]

Supporting Information

for

Insertion of CS_2 into Bi–N Bonds: Coordination Chemistry and Reactivity of Bismuth Dithiocarbamates

Gargi Kundu,[a,b]+ Frerik Wurm,[a,b]+ Kai Oberdorf,[a] Ahmed Fetoh,[a,b,c] Pulkit Dhankhar,[d] Celina Becker,[b,e] Johanna Heine,[d] Kerstin Volz,[b,e] and Crispin Lichtenberg*[a,b]

[a] Department of Chemistry, Philipps-University Marburg, Hans-Meerwein-Str. 4, D-35032 Marburg, Germany. E-mail: crispin.lichtenberg@chemie.uni-marburg.de

[b] mar.quest | Marburg Center for Quantum Materials and Sustainable Technologies, D-35032 Marburg, Germany

[c] Department of Chemistry, Faculty of Science, Mansoura University, Dakahlia Governorate 11432 Mansoura, Egypt

[d] Institute of Chemistry, Carl von Ossietzky Universität Oldenburg, Ammerländer Heerstraße 114-118, D-26129 Oldenburg, Germany

[e] Materials Science Center and Department of Physics, Philipps-University Marburg, Hans-Meerwein-Str. 6, D-35032 Marburg, Germany.

\+ These authors contributed equally to this work.

Table of Contents

I. NMR and HRMS Spectra S2

II. UV/Vis Spectra S27

III. P-XRD, EDX and SEM Data S28

IV. X-Ray Diffraction Analysis S30

V. Solid-State Structure of $[Bi(NMe_2)_2Cl]_\infty$ (**3-Cl**) S43

VI. Solid-State Structure of $[Bi(S_2CNMe_2)_3]_2$ (**6**) S44

VII. Solid-State Structure of $[CoCp_2][S_2CNMe_2]$ S45

VIII. Solid-State Structure of $[Bi(CAAC)Cl_2(S_2CNMe_2)_3]_2$ (**7**) S46

IX. References S47

I. NMR and HRMS Spectra

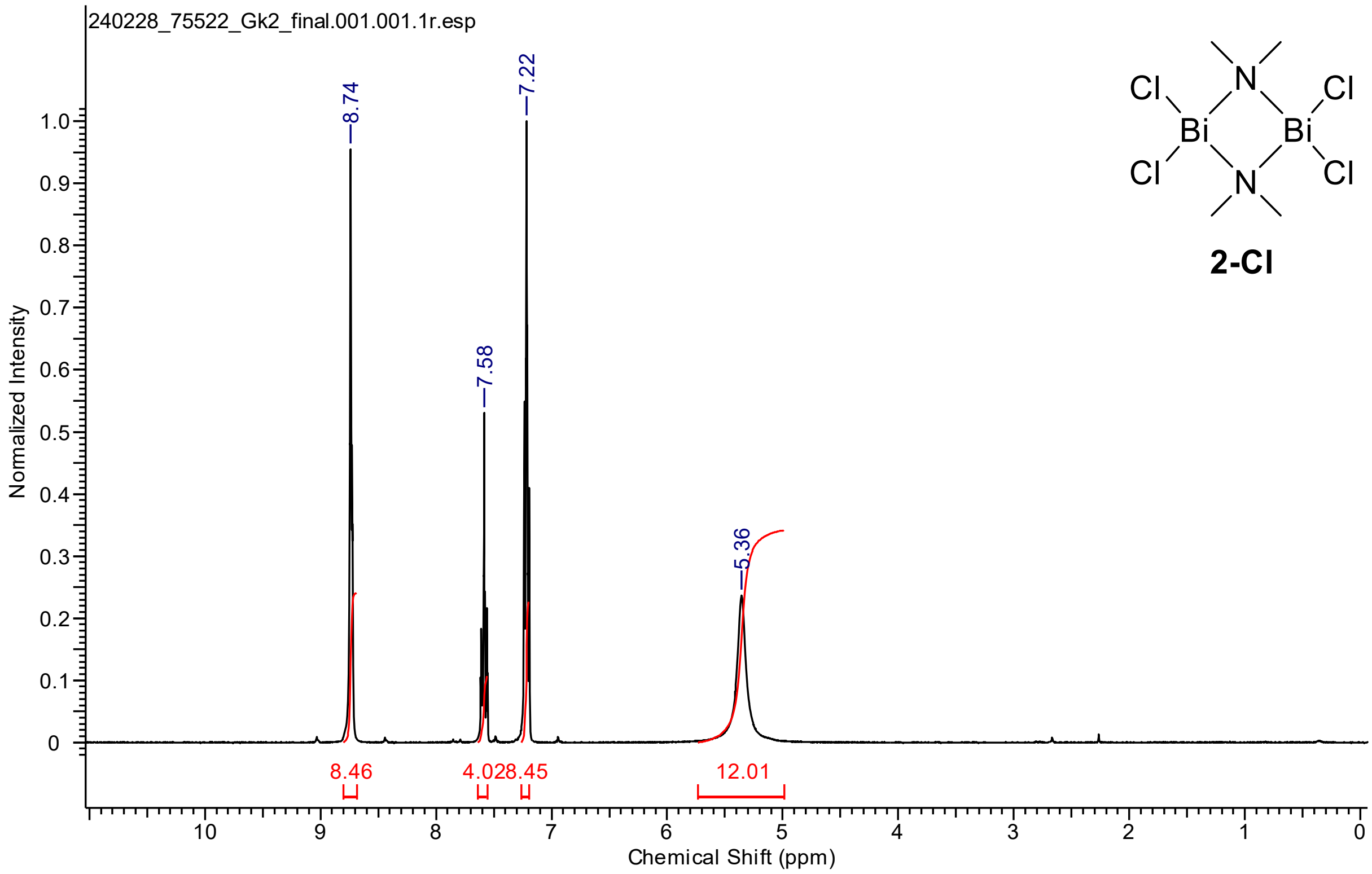

Figure S1: ^{1}H NMR spectrum of **2-Cl** (500 MHz, 298 K, Py-d_5).

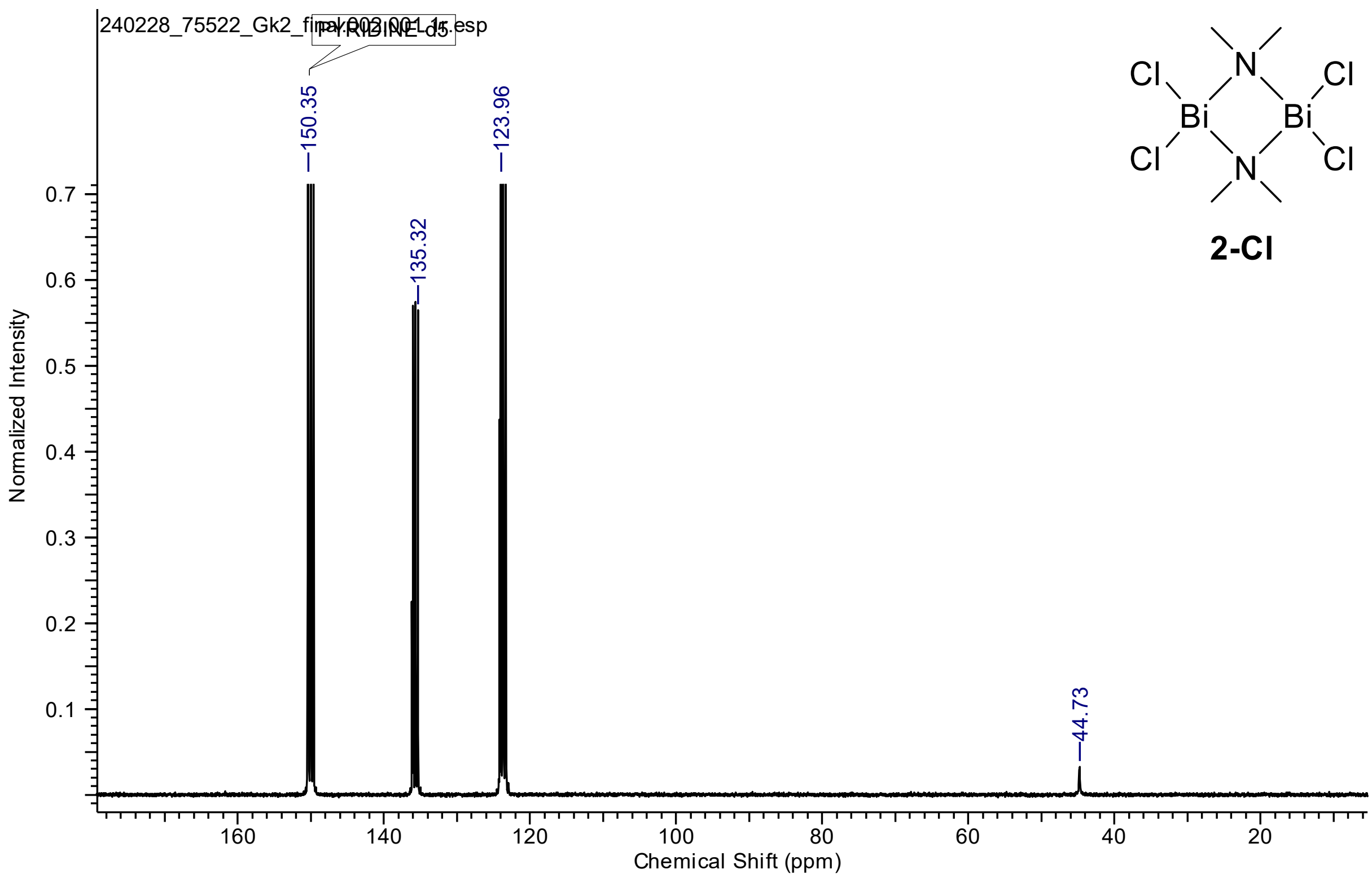

Figure S2: $^{13}C\{^{1}H\}$ NMR spectrum of **2-Cl** (126 MHz, 298 K, Py-d_5).

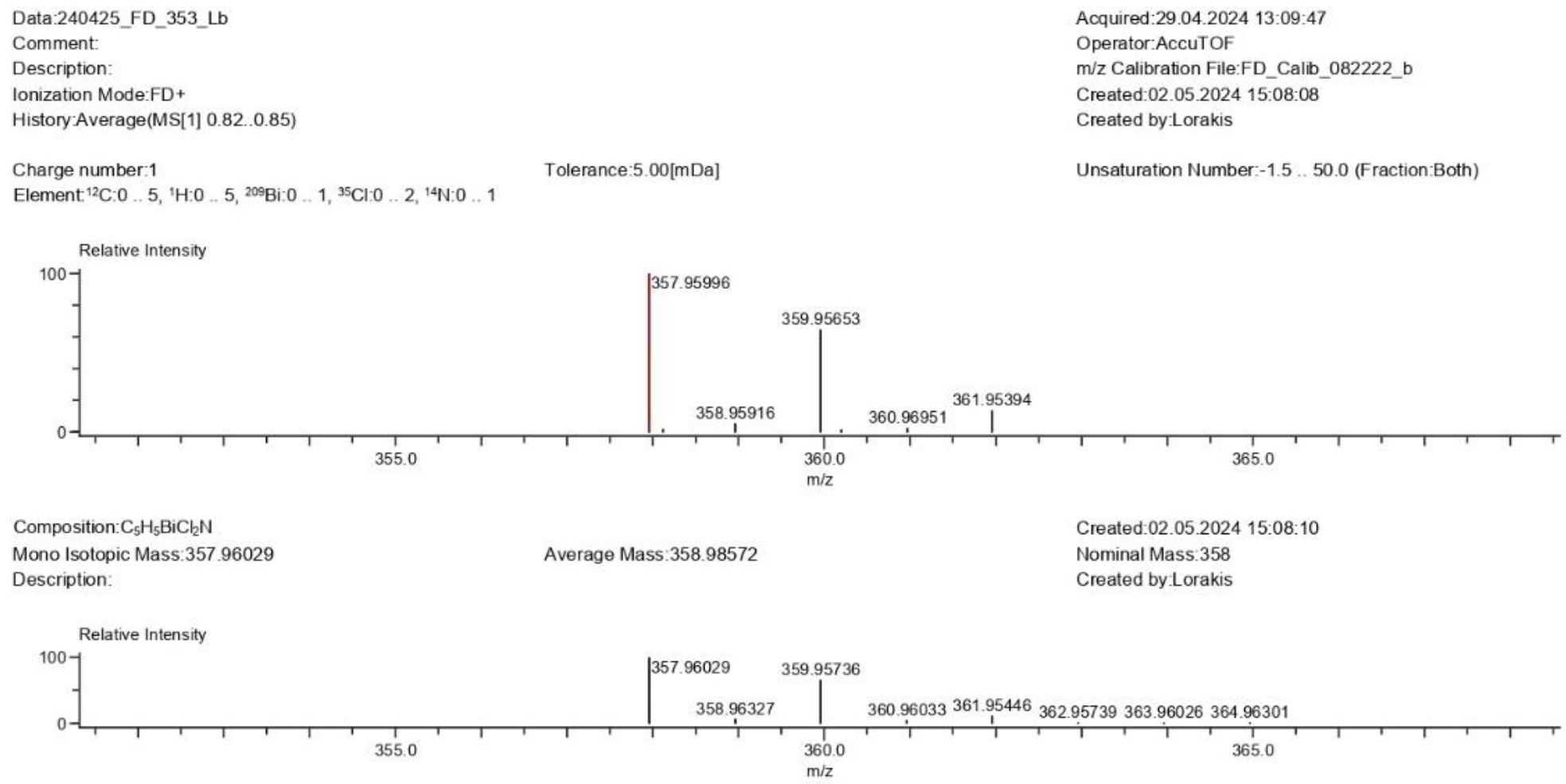

Mass	Intensity	Relative Intensity	Calc. Mass	Mass Difference [mDa]	Mass Difference [ppm]	Possible Formula	Unsaturation Number
357.95996	6493.72	100.00	357.96029	-0.32	-0.91	$^{12}C_5{}^1H_5{}^{209}Bi_1{}^{35}Cl_2{}^{14}N_1$	3.5

Figure S3: HR-MS (LIFDI, pos.) spectrum of **2-Cl**.

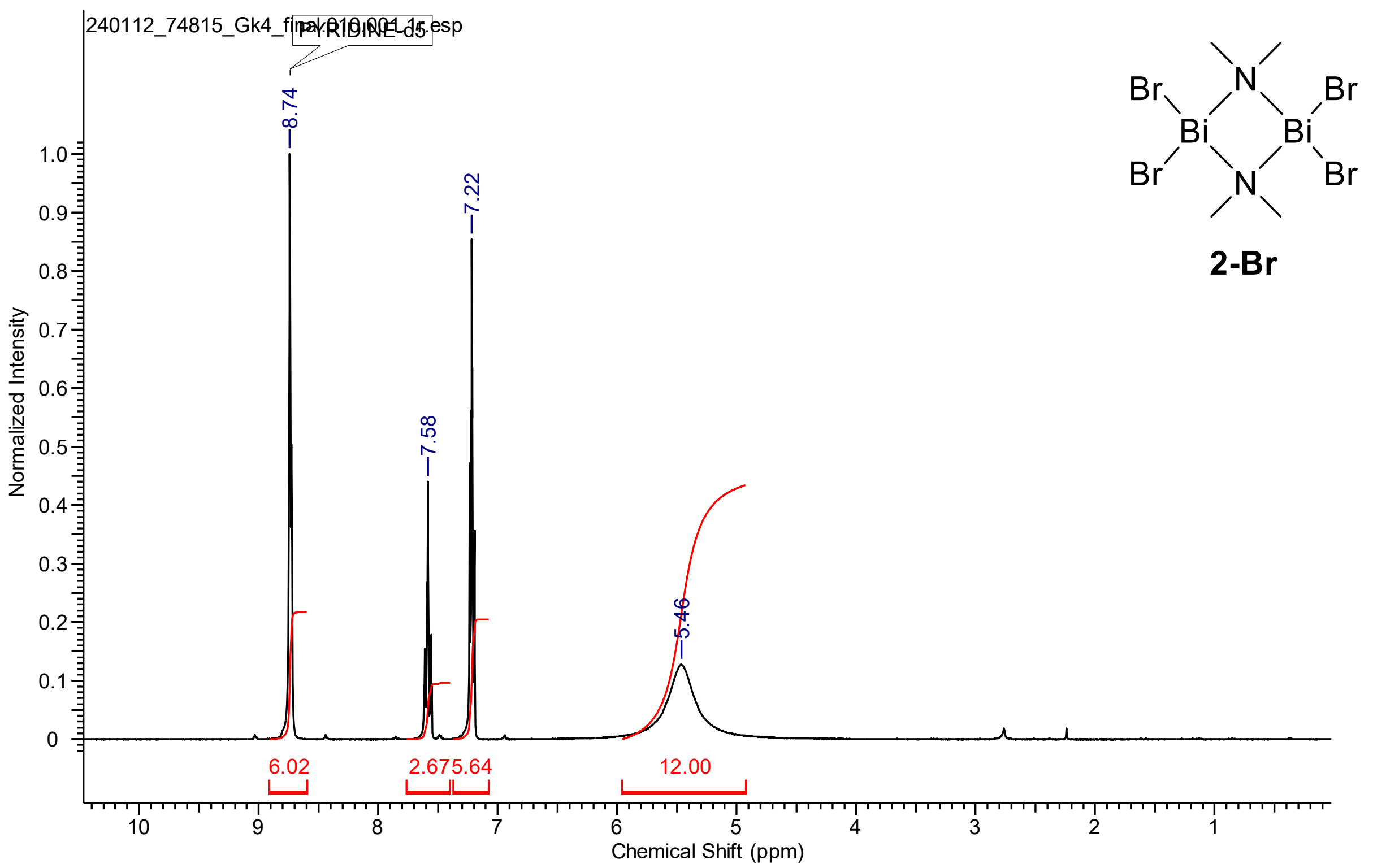

Figure S4: ^{1}H NMR spectrum of **2-Br** (500 MHz, 298 K, Py-d_5).

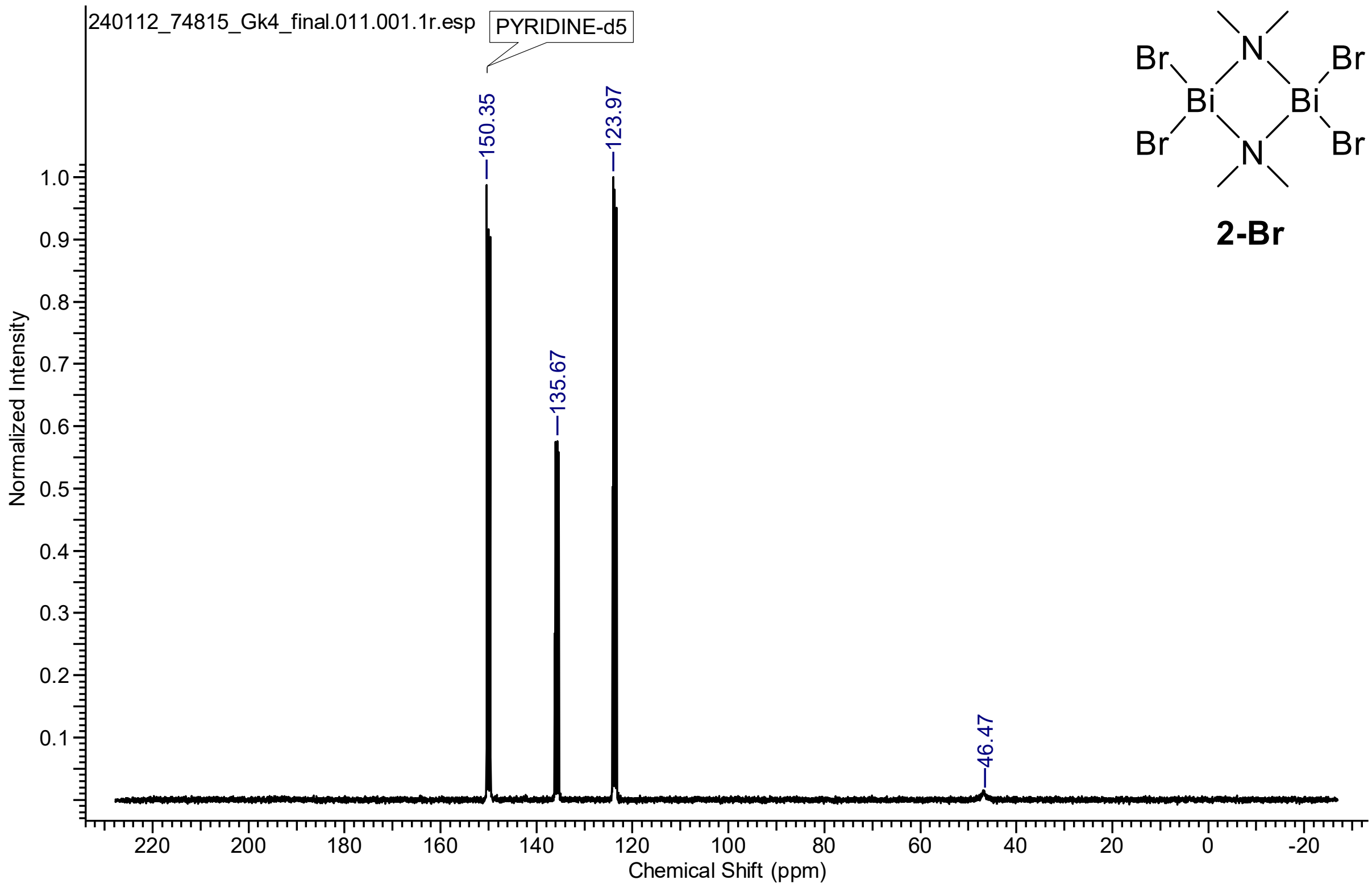

Figure S5: $^{13}C\{^{1}H\}$ NMR spectrum of **2-Br** (126 MHz, 298 K, Py-d_5).

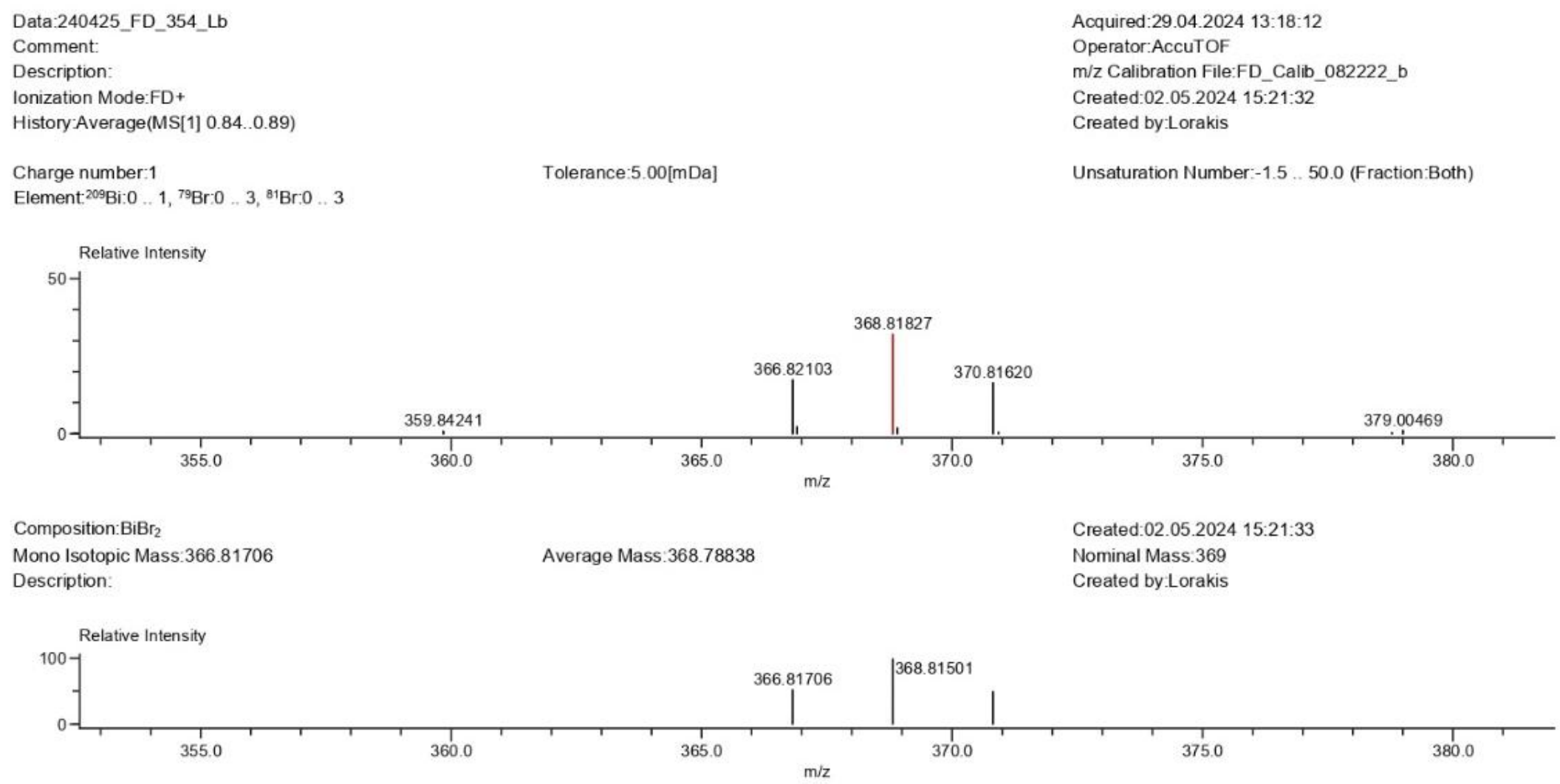

Mass	Intensity	Relative Intensity	Calc. Mass	Mass Difference [mDa]	Mass Difference [ppm]	Possible Formula	Unsaturation Number
368.81827	4500.53	31.85	368.81501	3.26	8.84	$^{209}Bi_1{}^{79}Br_1{}^{81}Br_1$	0.5

Figure S6: HR-MS (LIFDI, pos.) spectrum of **2-Br**.

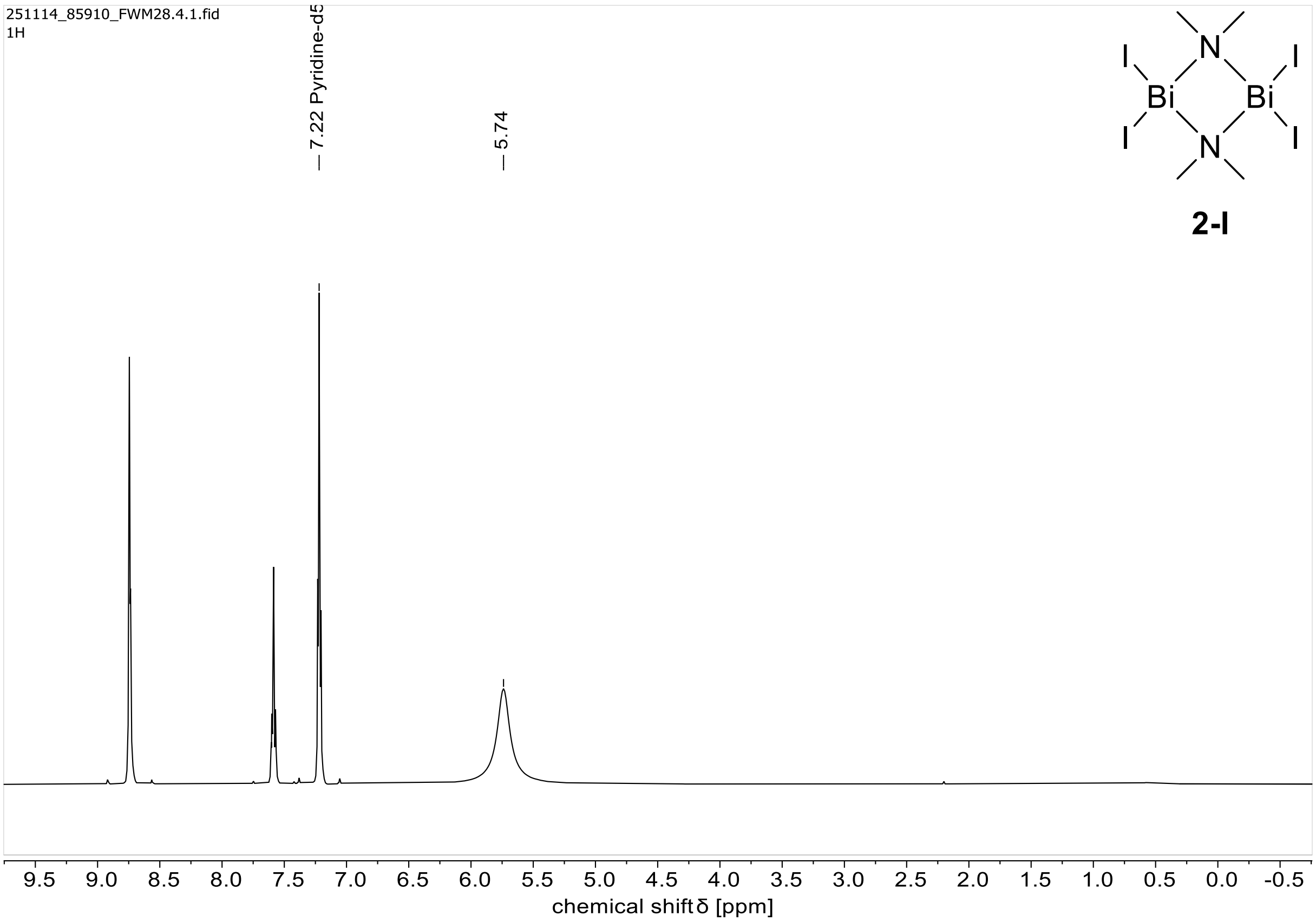

Figure S7: ^{1}H NMR spectrum of **2-I** (500 MHz, 298 K, Py-d_5).

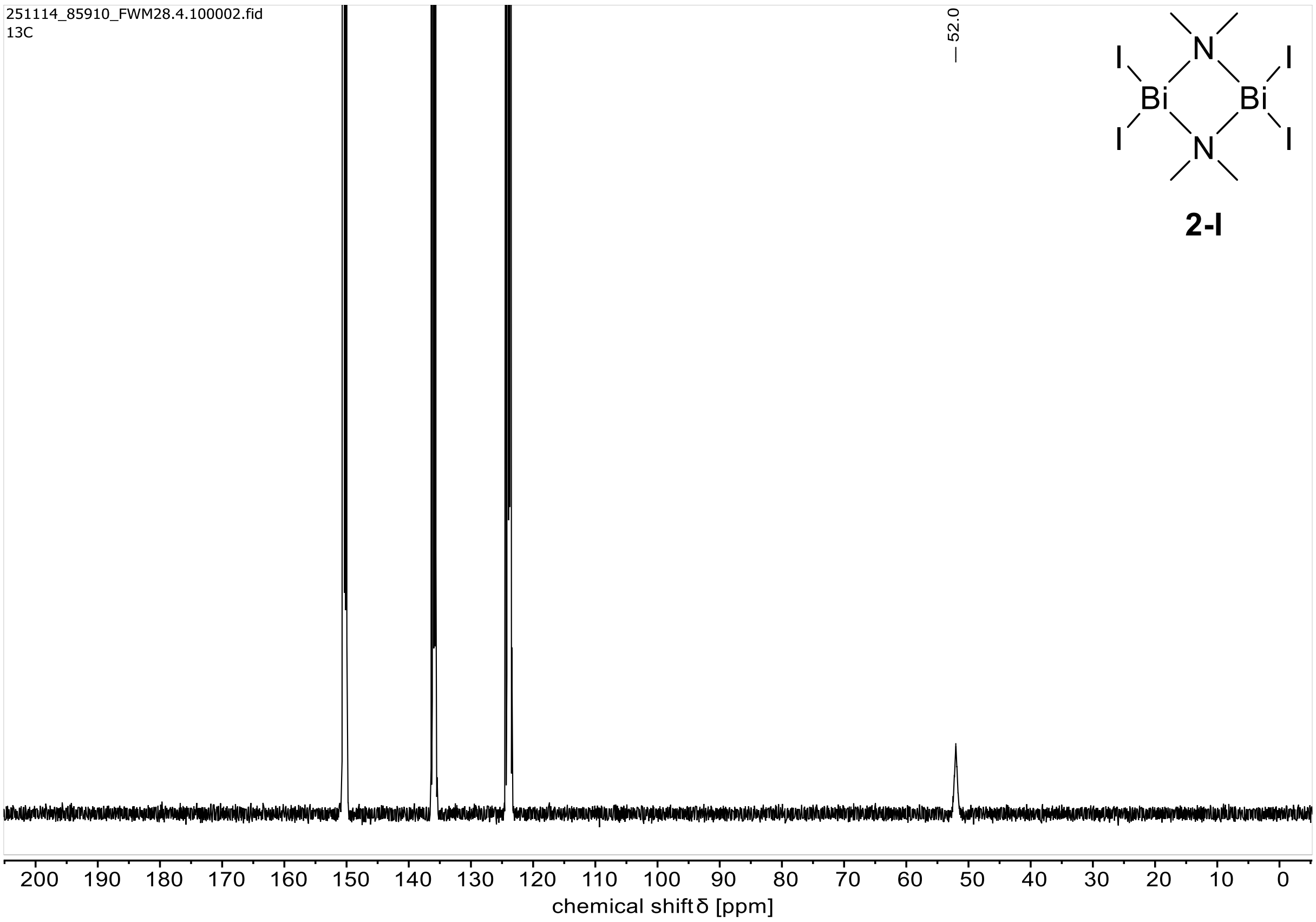

Figure S8: $^{13}C\{^{1}H\}$ NMR spectrum of **2-I** (126 MHz, 298 K, Py-d_5).

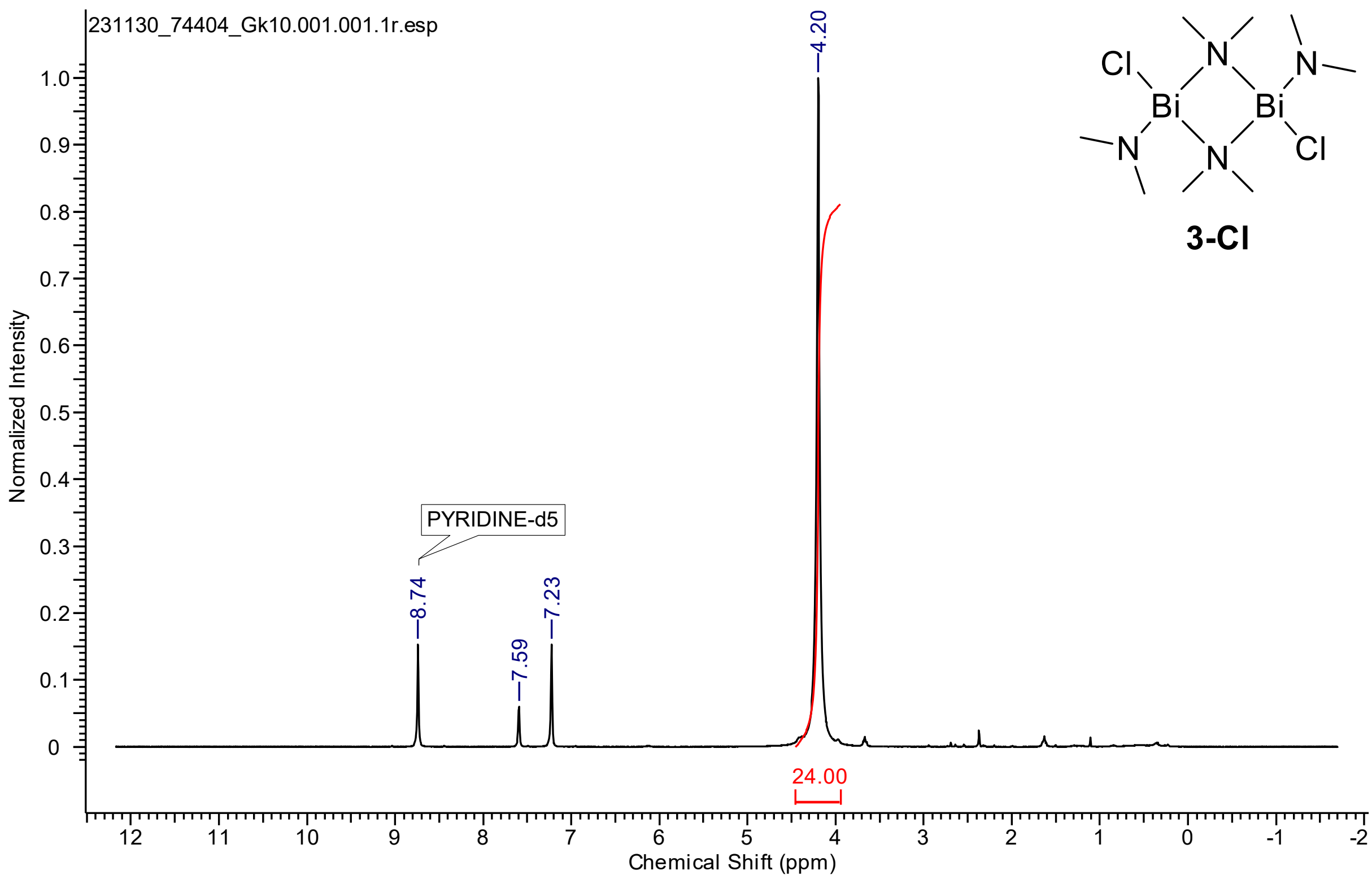

Figure S9: ^{1}H NMR spectrum of **3-Cl** (500 MHz, 298 K, Py-d_5).

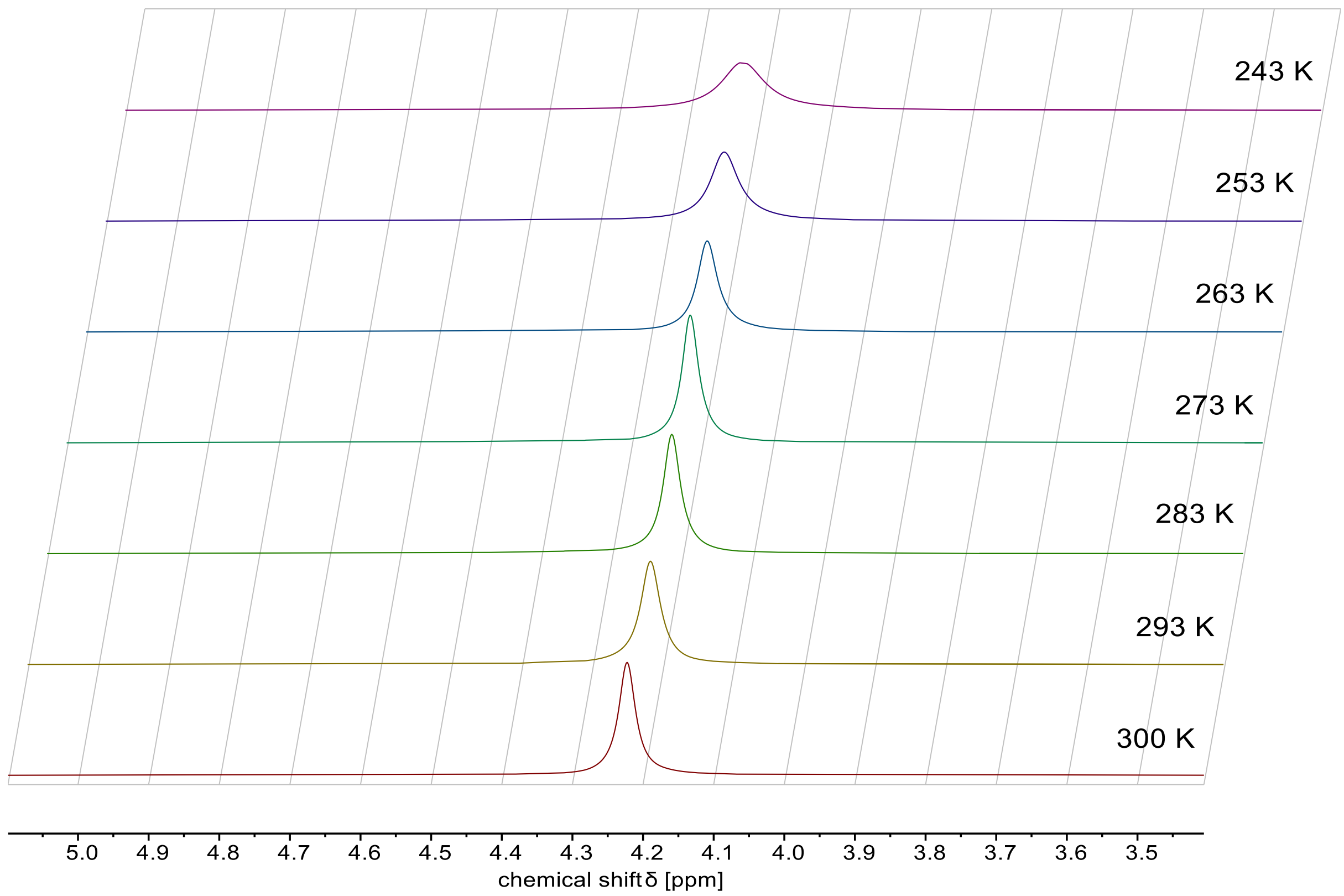

Figure S10: ^{1}H NMR spectra of **3-Cl** in the temperature range of 300 K – 243 K showing the expected signal broadening at lower temperatures (500 MHz, Py-d_5). All spectra are standardized on the intensity of the solvent resonance.

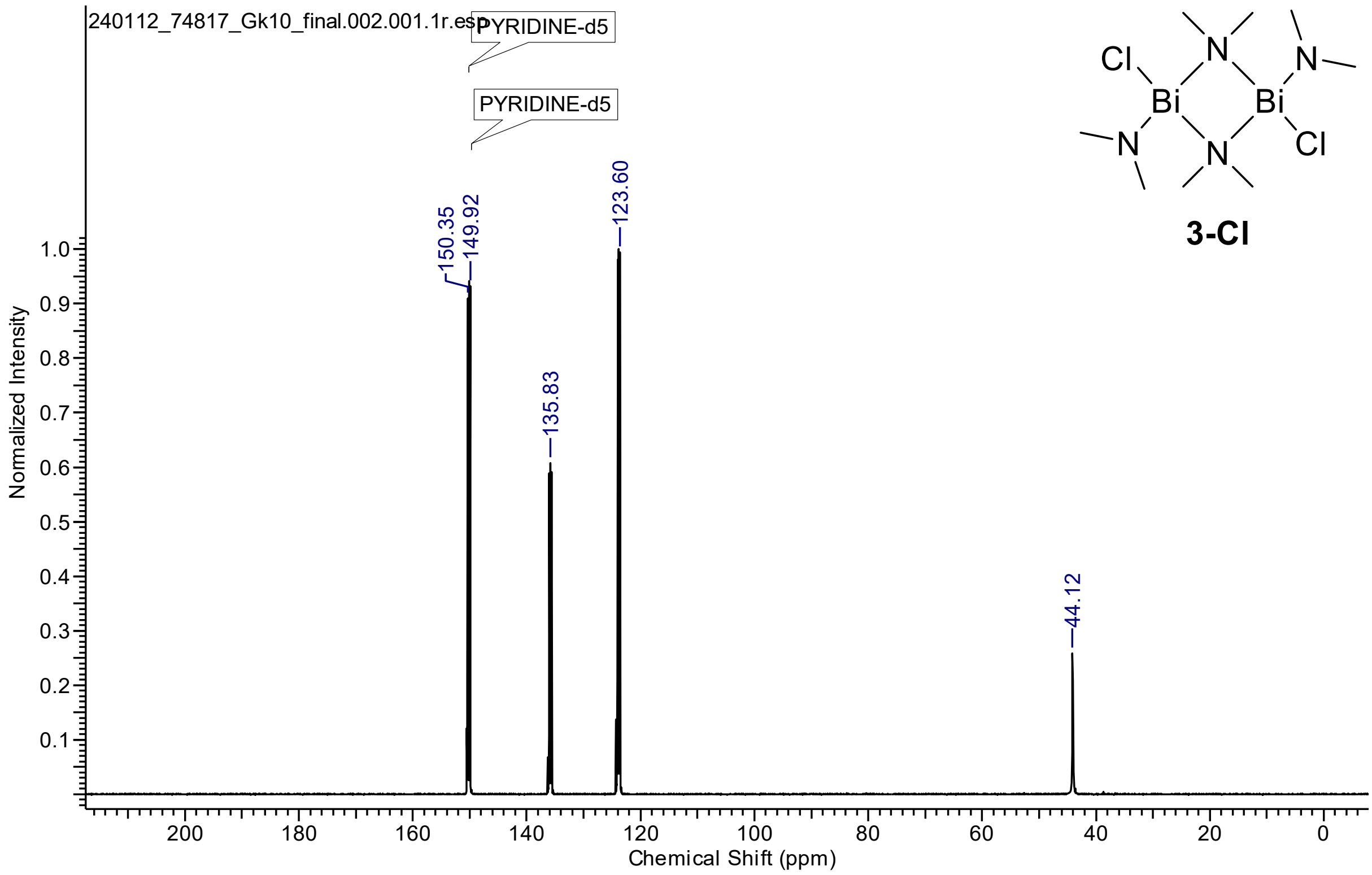

Figure S11: $^{13}C\{^{1}H\}$ NMR spectrum of **3-Cl** (126 MHz, 298 K, Py-d_5).

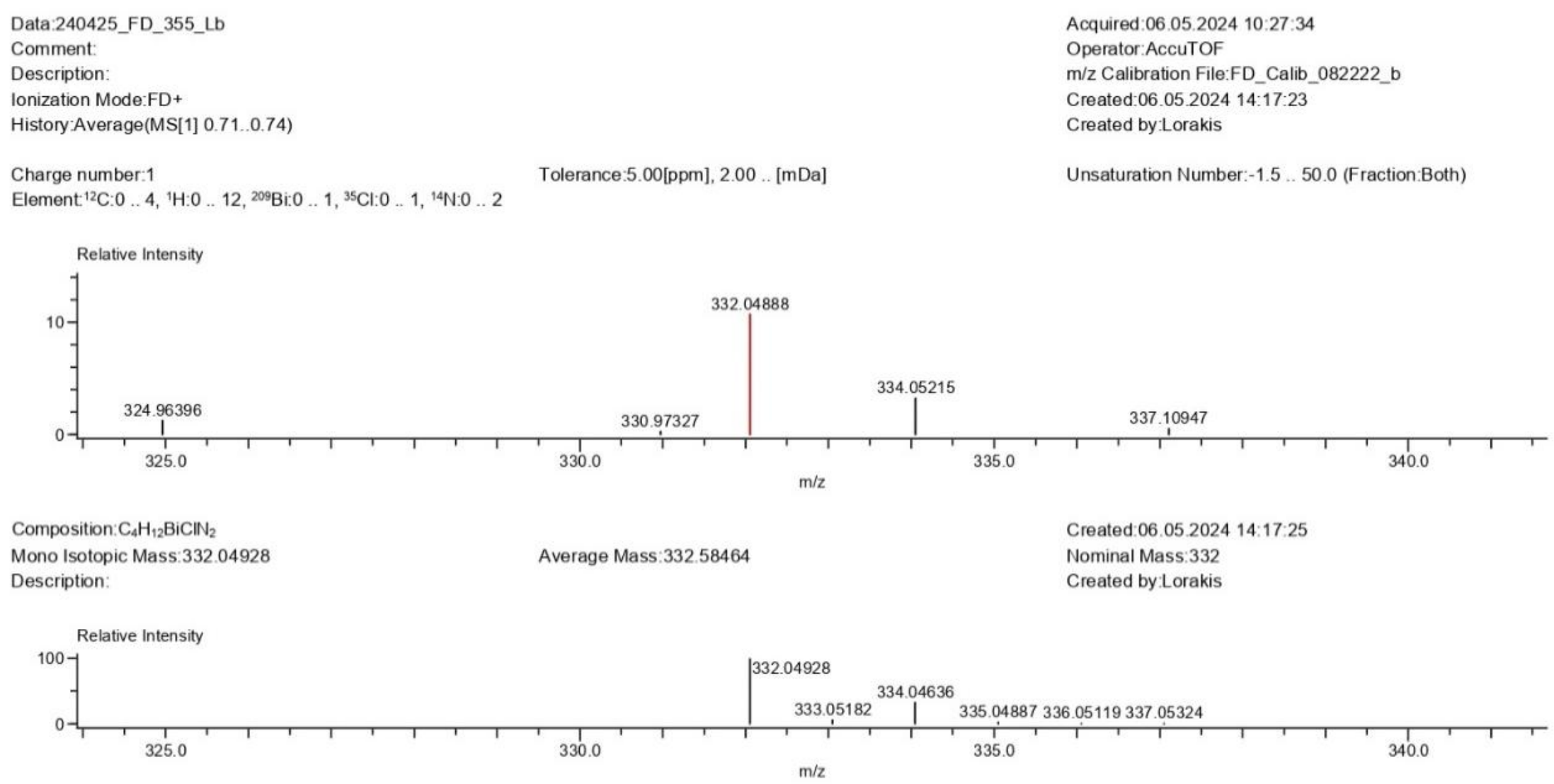

Mass	Intensity	Relative Intensity	Calc. Mass	Mass Difference [mDa]	Mass Difference [ppm]	Possible Formula	Unsaturation Number
332.04888	2440.62	10.68	332.04928	-0.41	-1.22	$^{12}C_4{}^{1}H_{12}{}^{209}Bi_1{}^{35}Cl_1{}^{14}N_2$	0.0

Figure S12: HR-MS (LIFDI, pos.) spectrum of **3-Cl**.

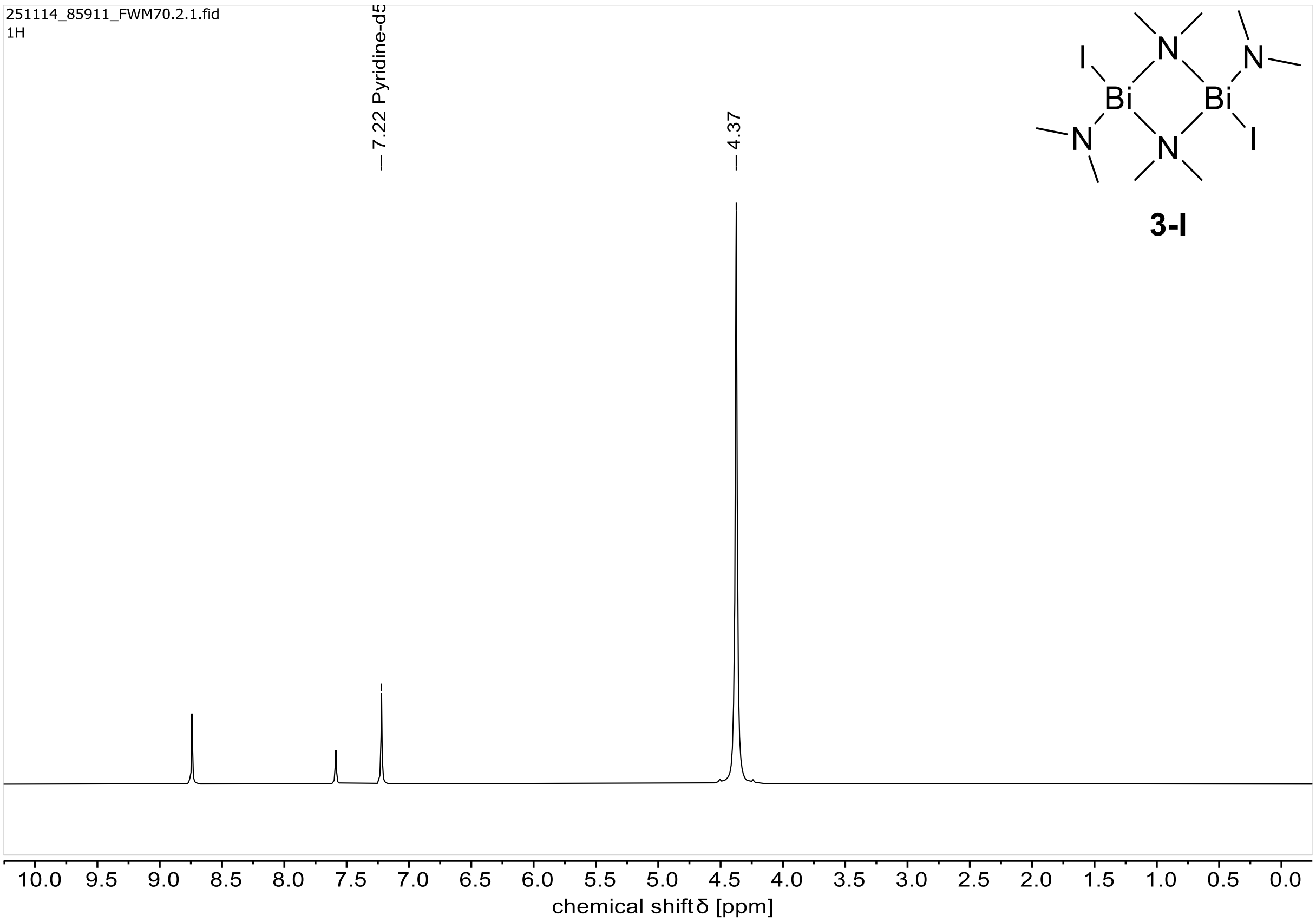

Figure S13: ^{1}H NMR spectrum of **3-I** (500 MHz, 298 K, Py-d_5).

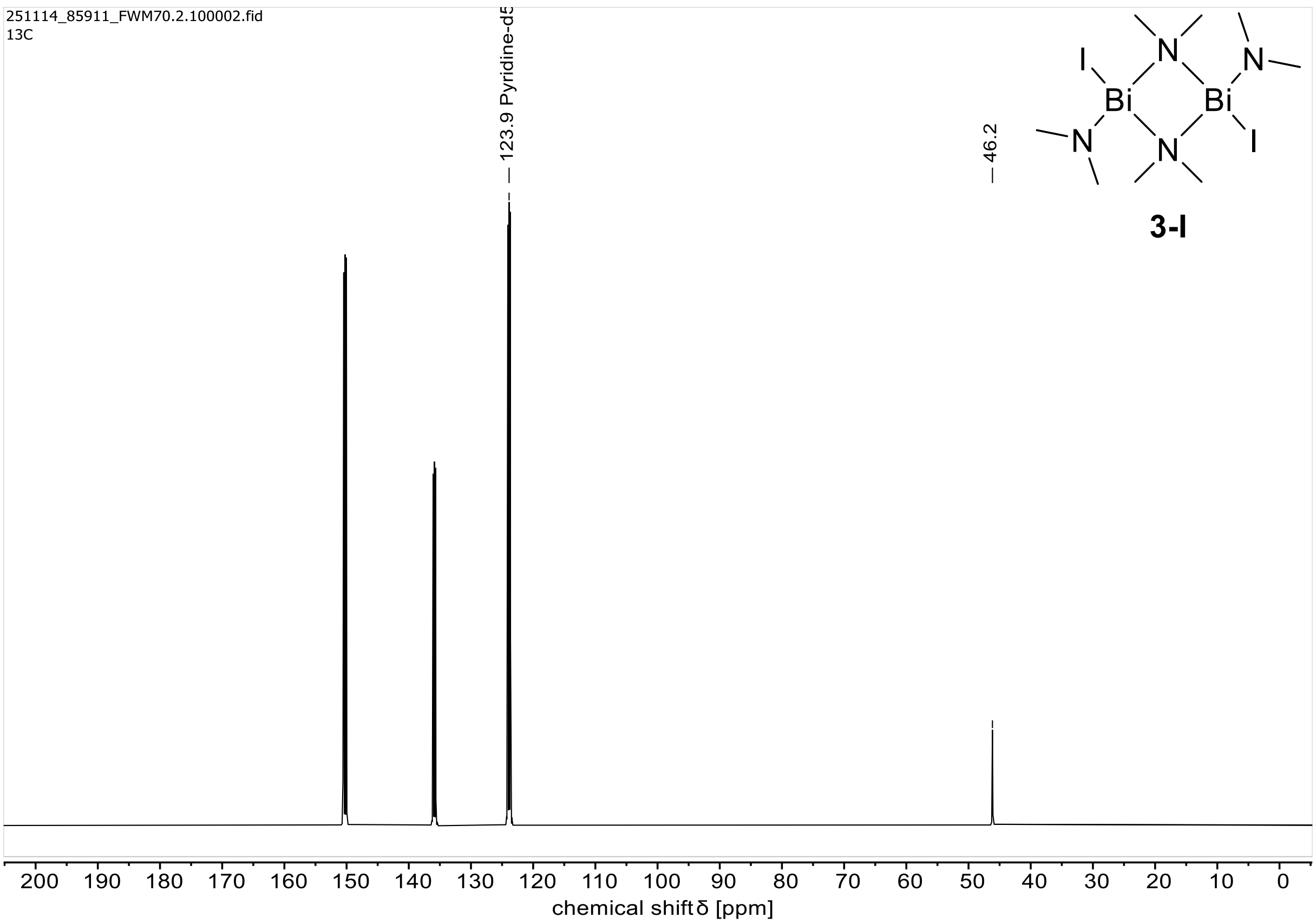

Figure S14: ^{13}C{^{1}H} NMR spectrum of **3-I** (126 MHz, 298 K, Py-d_5).

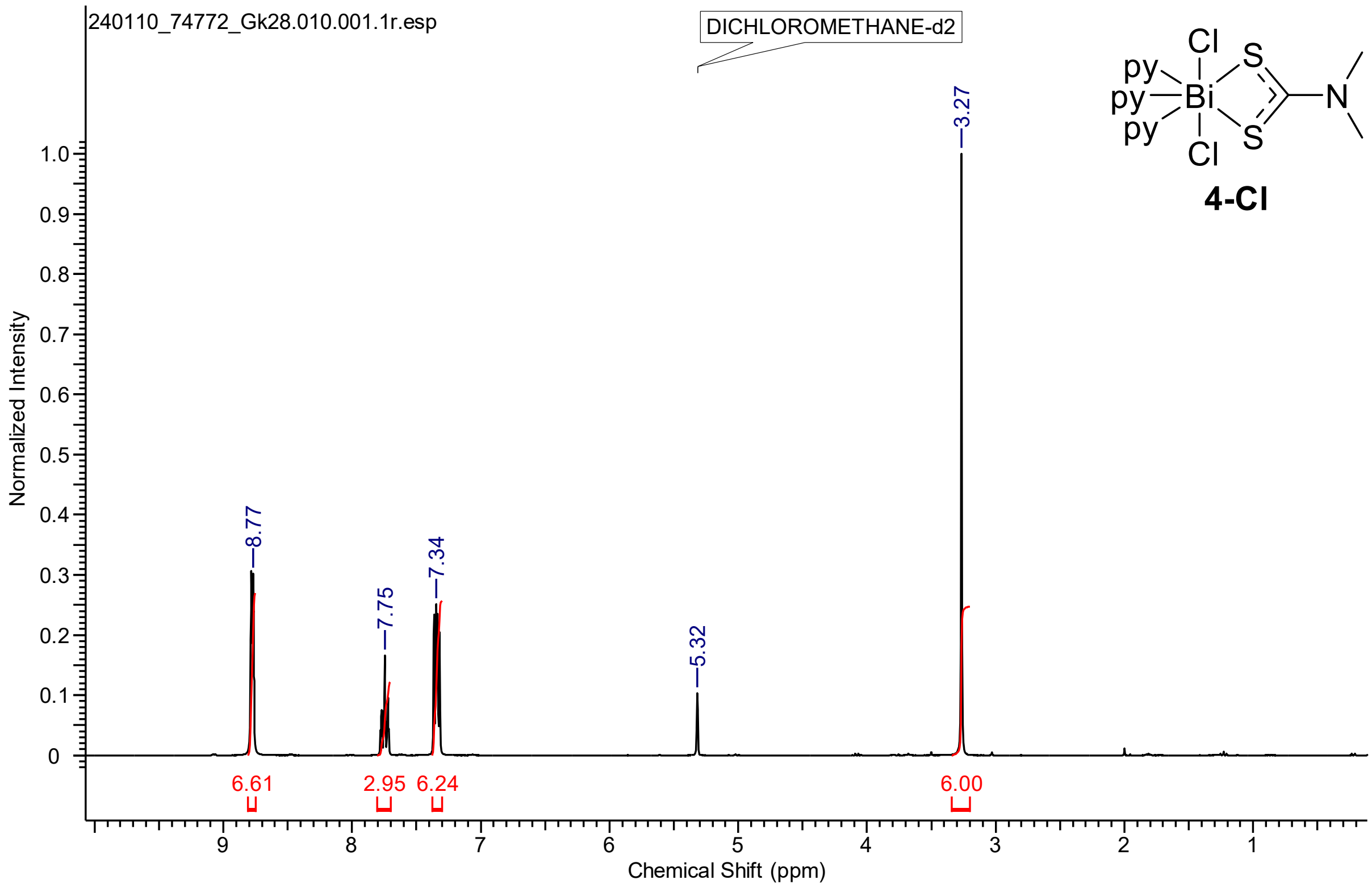

Figure S15: ^{1}H NMR spectrum of **4-Cl** (500 MHz, 298 K, CD_2Cl_2).

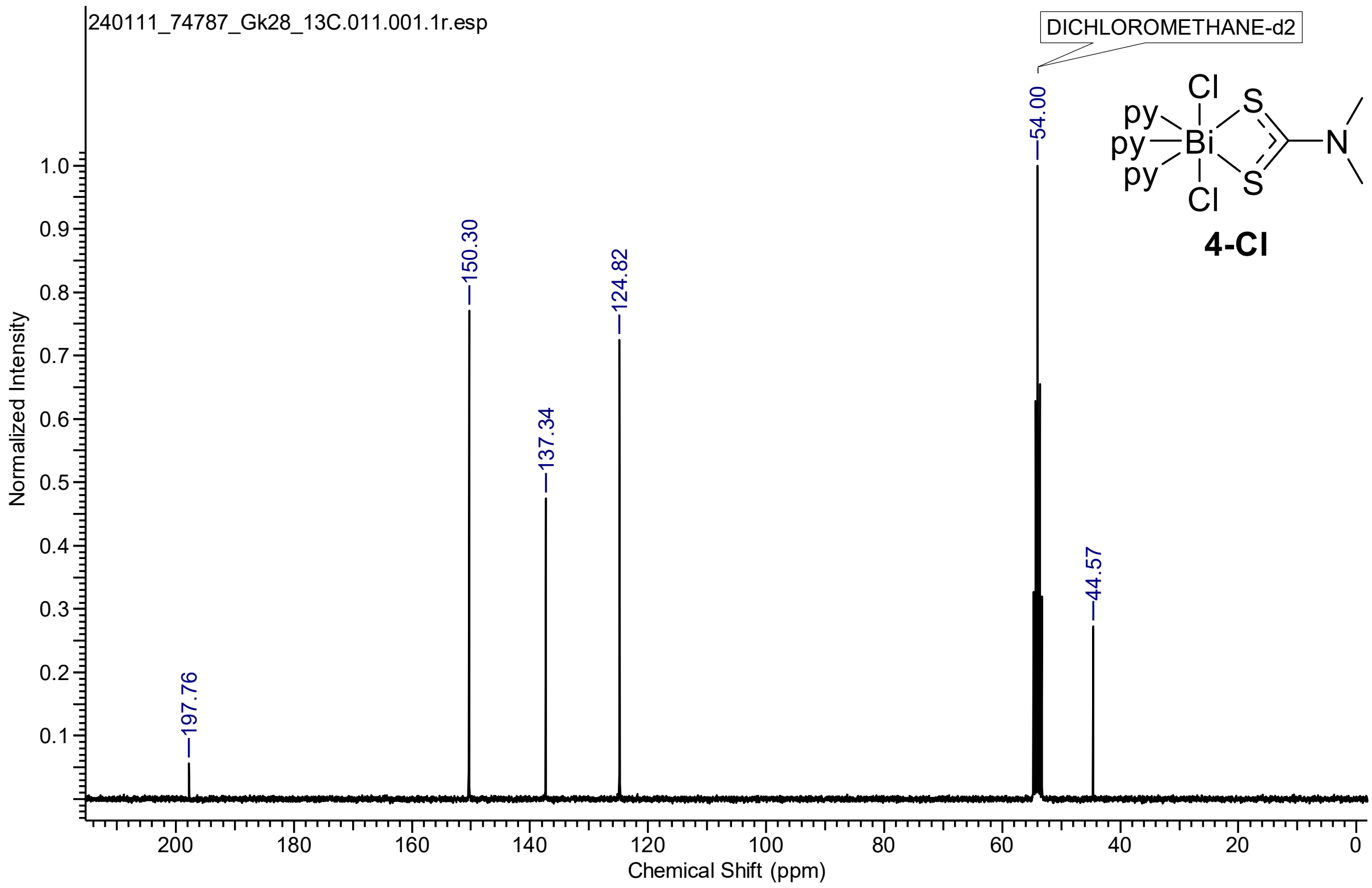

Figure S16: $^{13}C\{^{1}H\}$ NMR spectrum of **4-Cl** (126 MHz, 298 K, CD_2Cl_2).

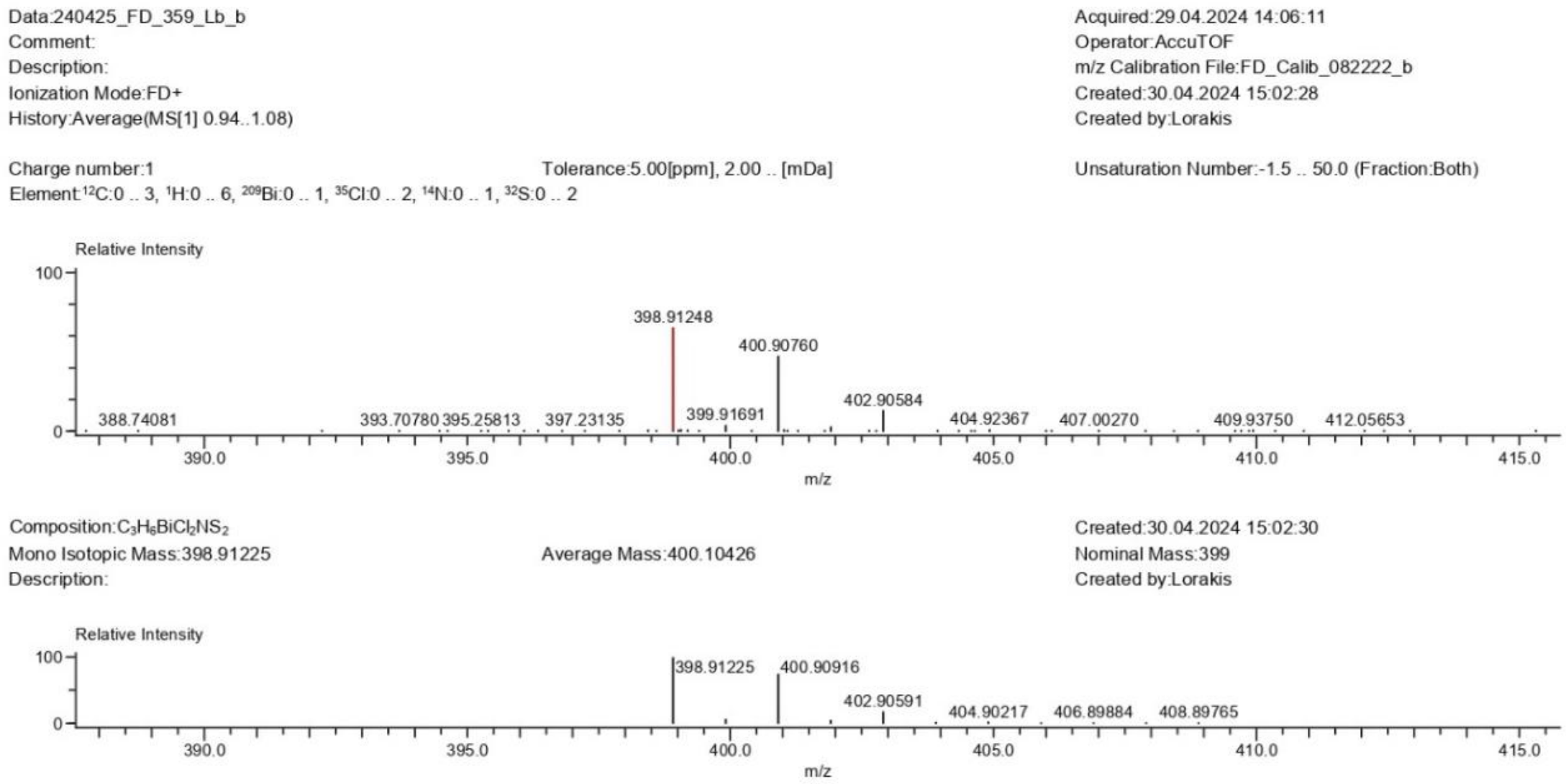

Mass	Intensity	Relative Intensity	Calc. Mass	Mass Difference [mDa]	Mass Difference [ppm]	Possible Formula	Unsaturation Number
398.91248	6767.96	64.95	398.91225	0.23	0.58	$^{12}C_3{}^1H_6{}^{209}Bi_1{}^{35}Cl_2{}^{14}N_1{}^{32}S_2$	3.0

Figure S17: HR-MS (LIFDI, pos.) spectrum of **4-Cl**.

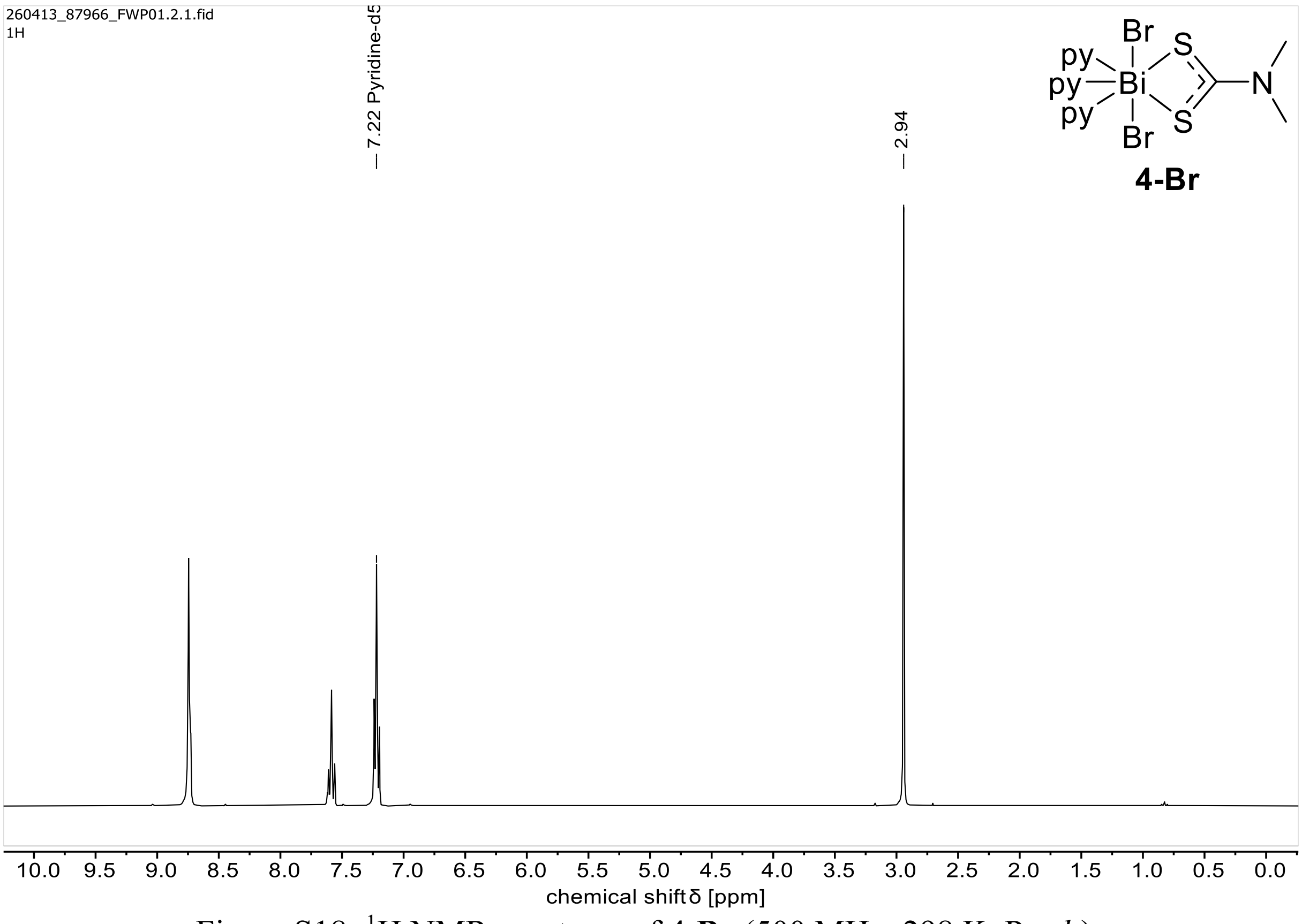

Figure S18: ^{1}H NMR spectrum of **4-Br** (500 MHz, 298 K, Py-d_5).

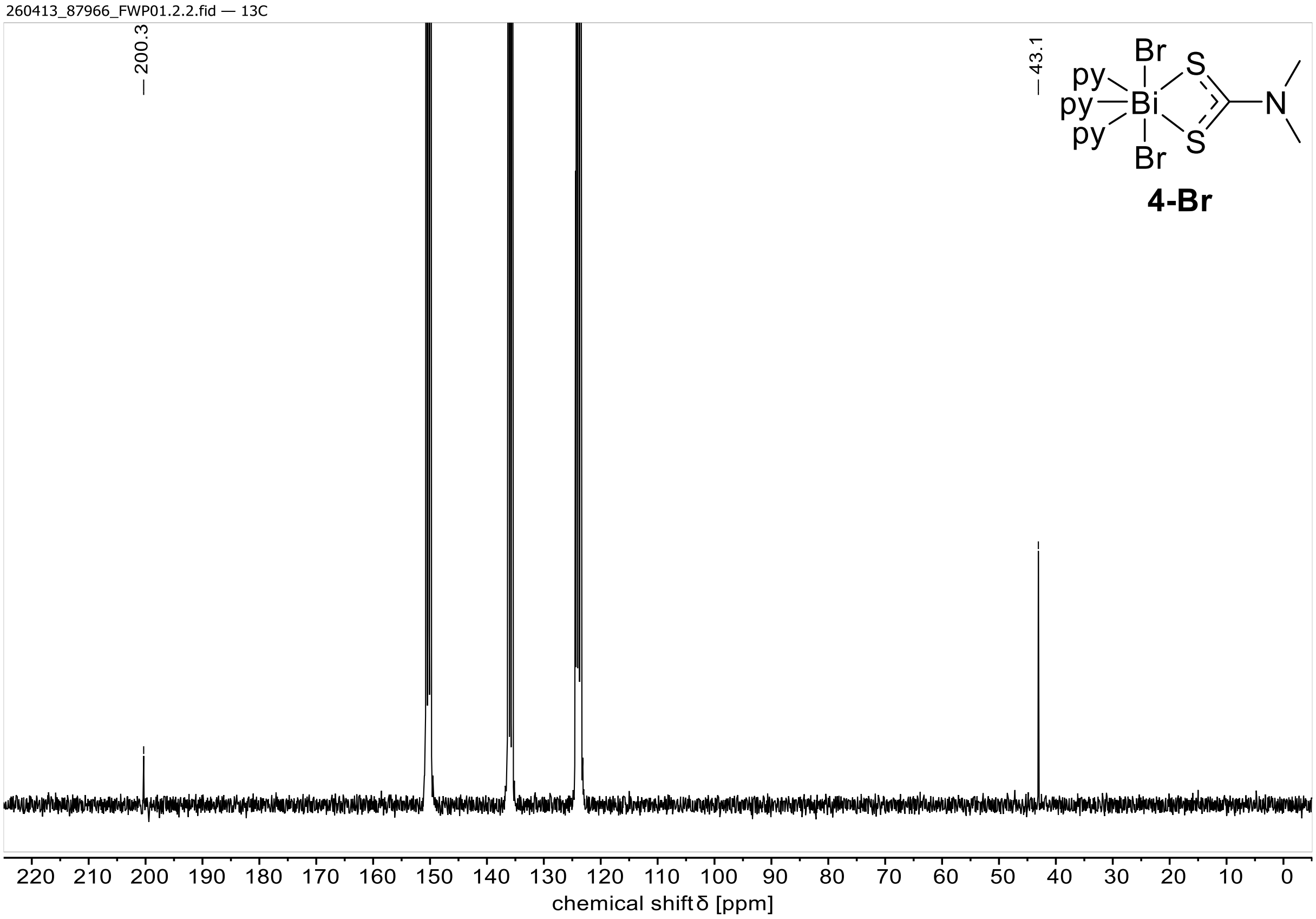

Figure S19: $^{13}C\{^{1}H\}$ NMR spectrum of **4-Br** (126 MHz, 298 K, Py-d_5).

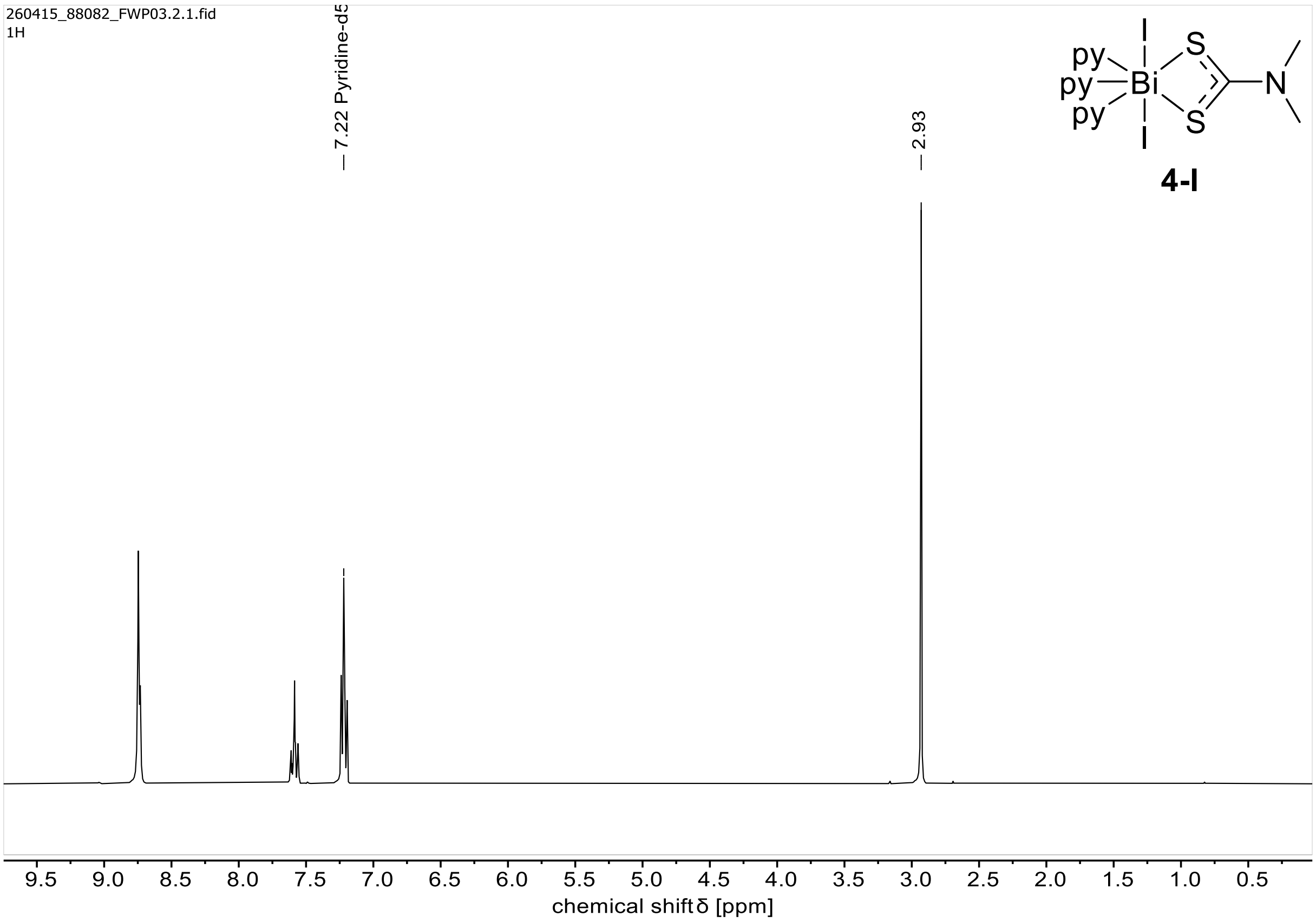

Figure S20: ^{1}H NMR spectrum of **4-I** (500 MHz, 298 K, Py-d_5).

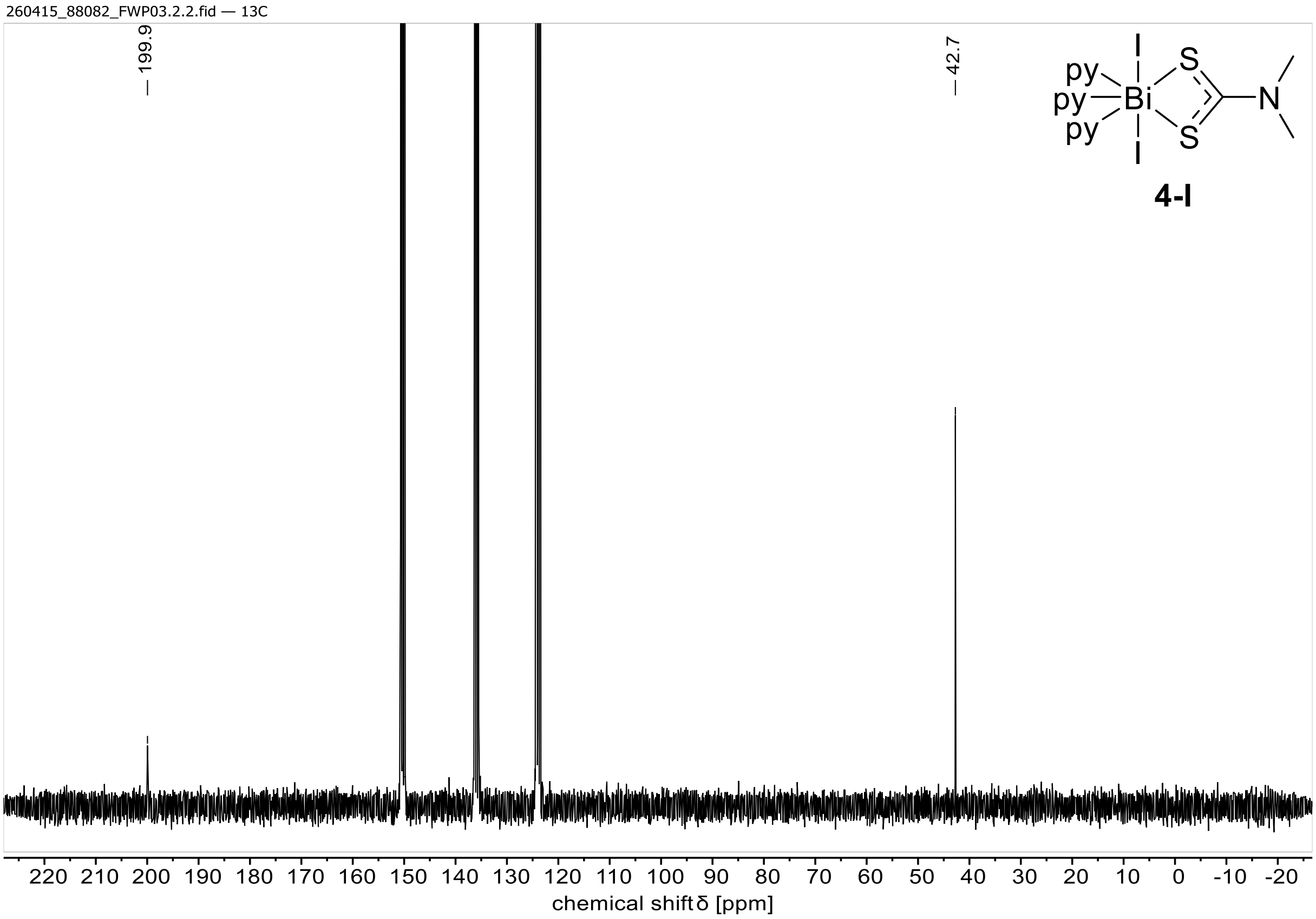

Figure S21: $^{13}C\{^{1}H\}$ NMR spectrum of **4-I** (126 MHz, 298 K, Py-d_5).

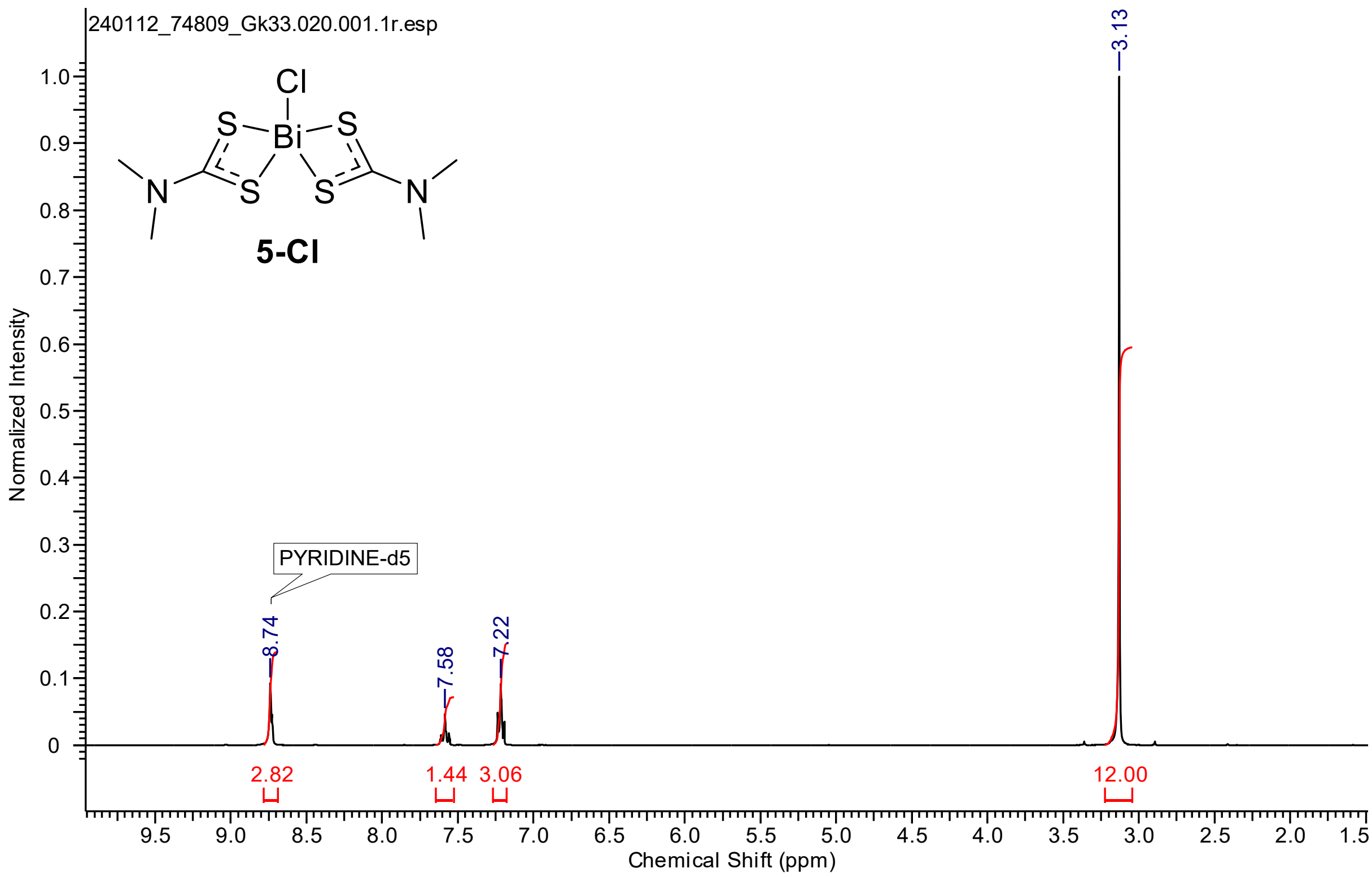

Figure S22: ^{1}H NMR spectrum of **5-Cl** (500 MHz, 298 K, Py-d_5).

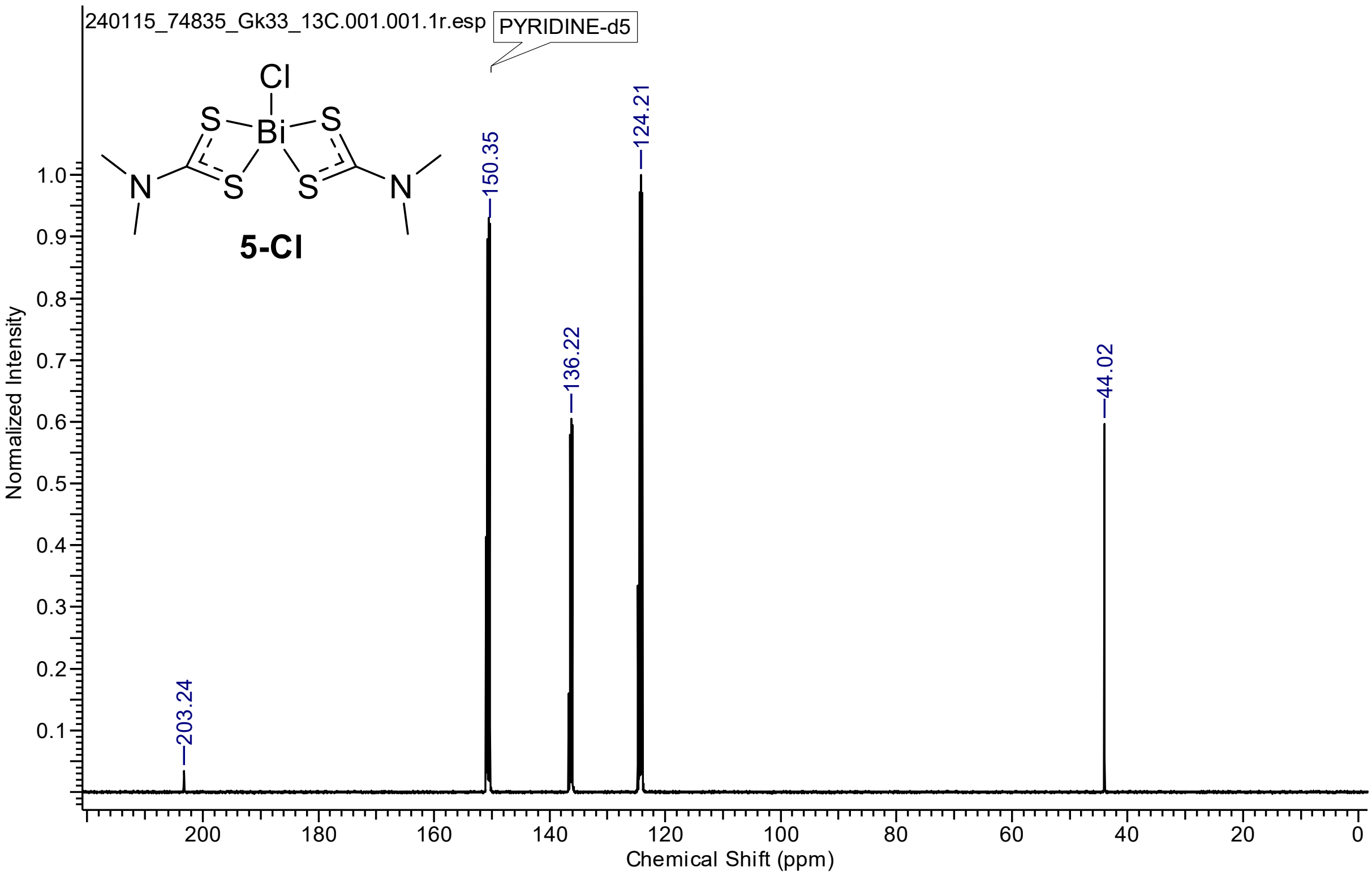

Figure S23: $^{13}C\{^1H\}$ NMR spectrum of **5-Cl** (126 MHz, 298 K, Py-d_5).

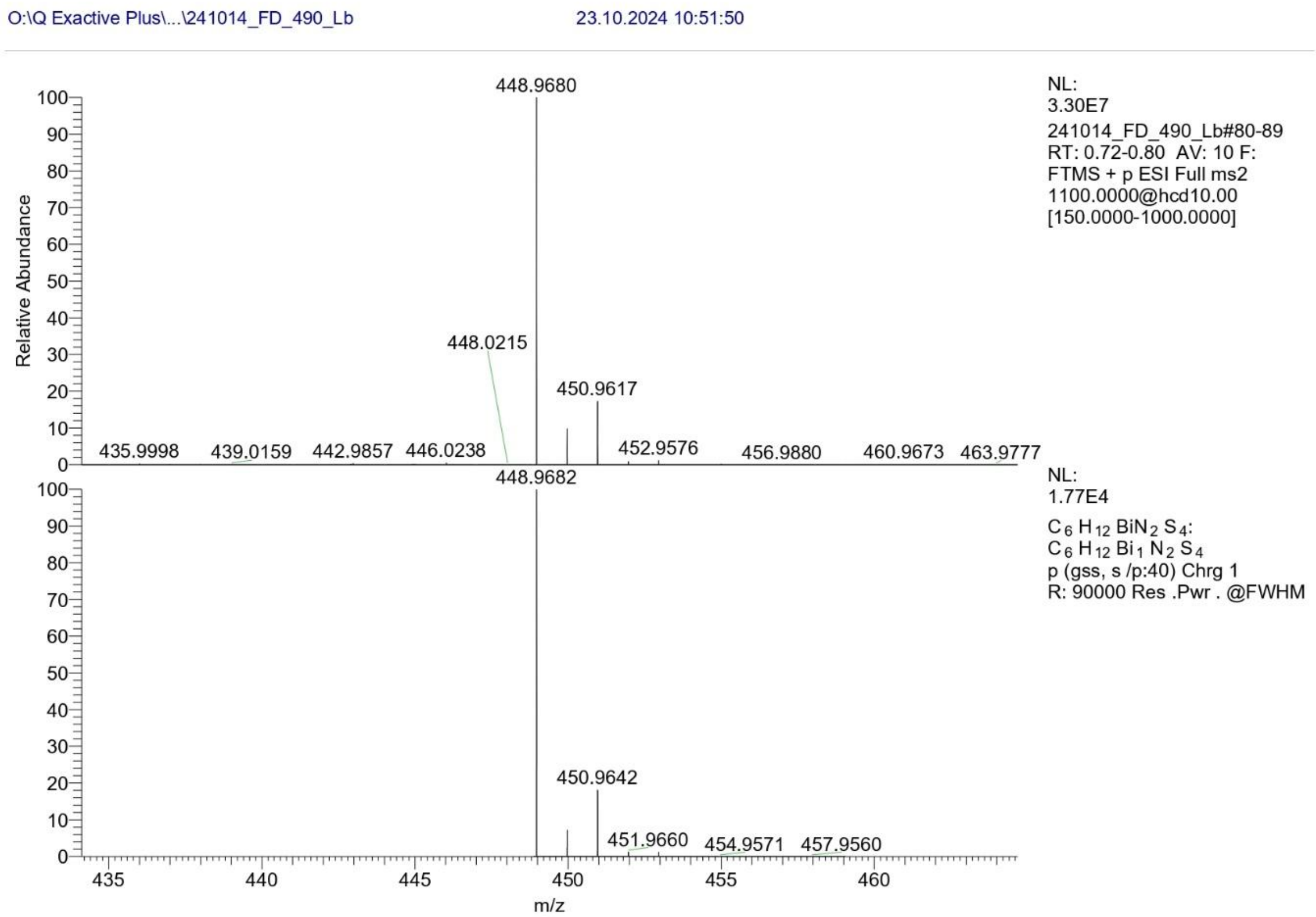

Figure S24: HR-MS (LIFDI, pos.) spectrum of **5-Cl**.

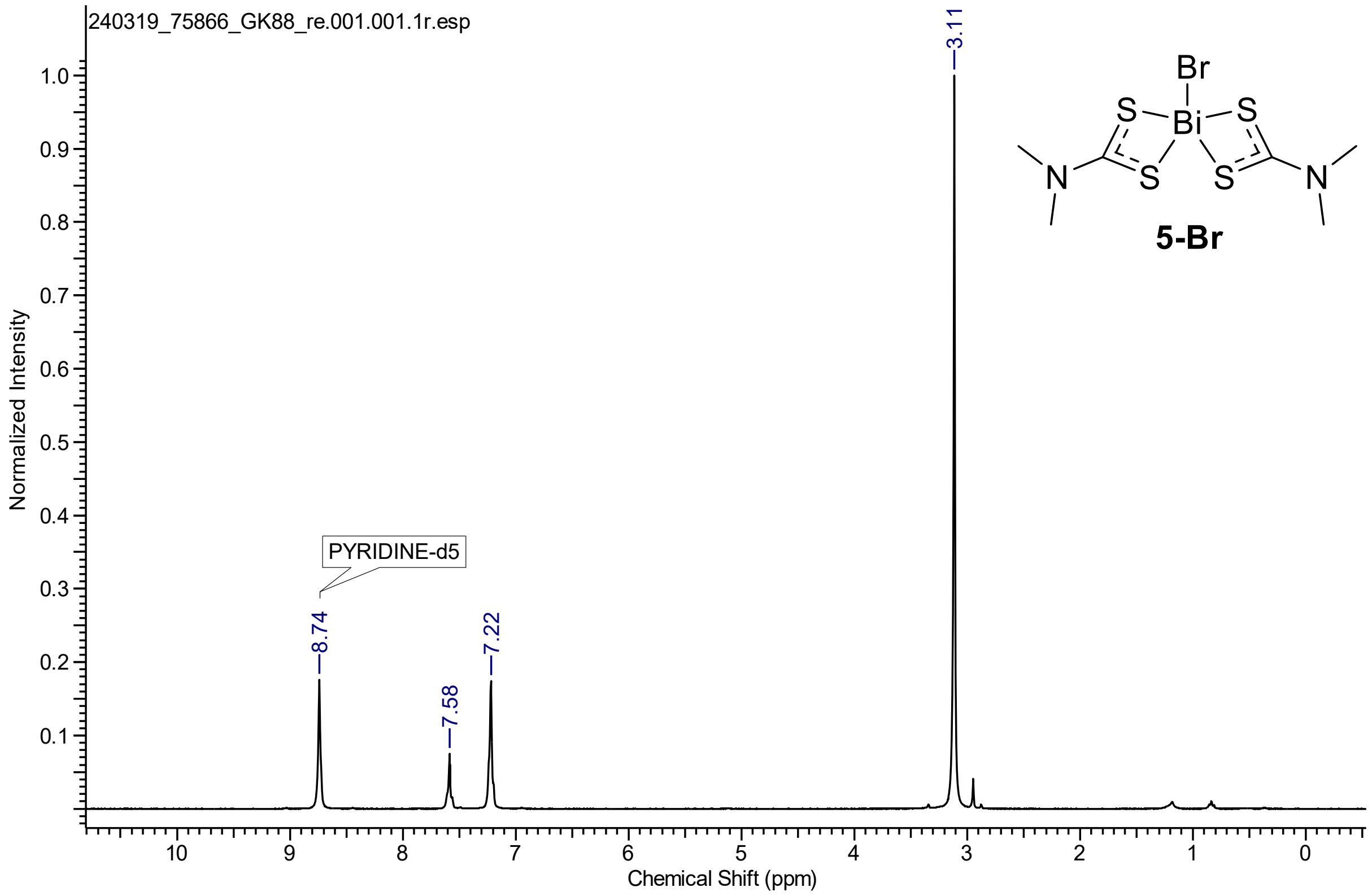

Figure S25: ^{1}H NMR spectrum of **5-Br** (500 MHz, 298 K, Py-*d*$_5$).

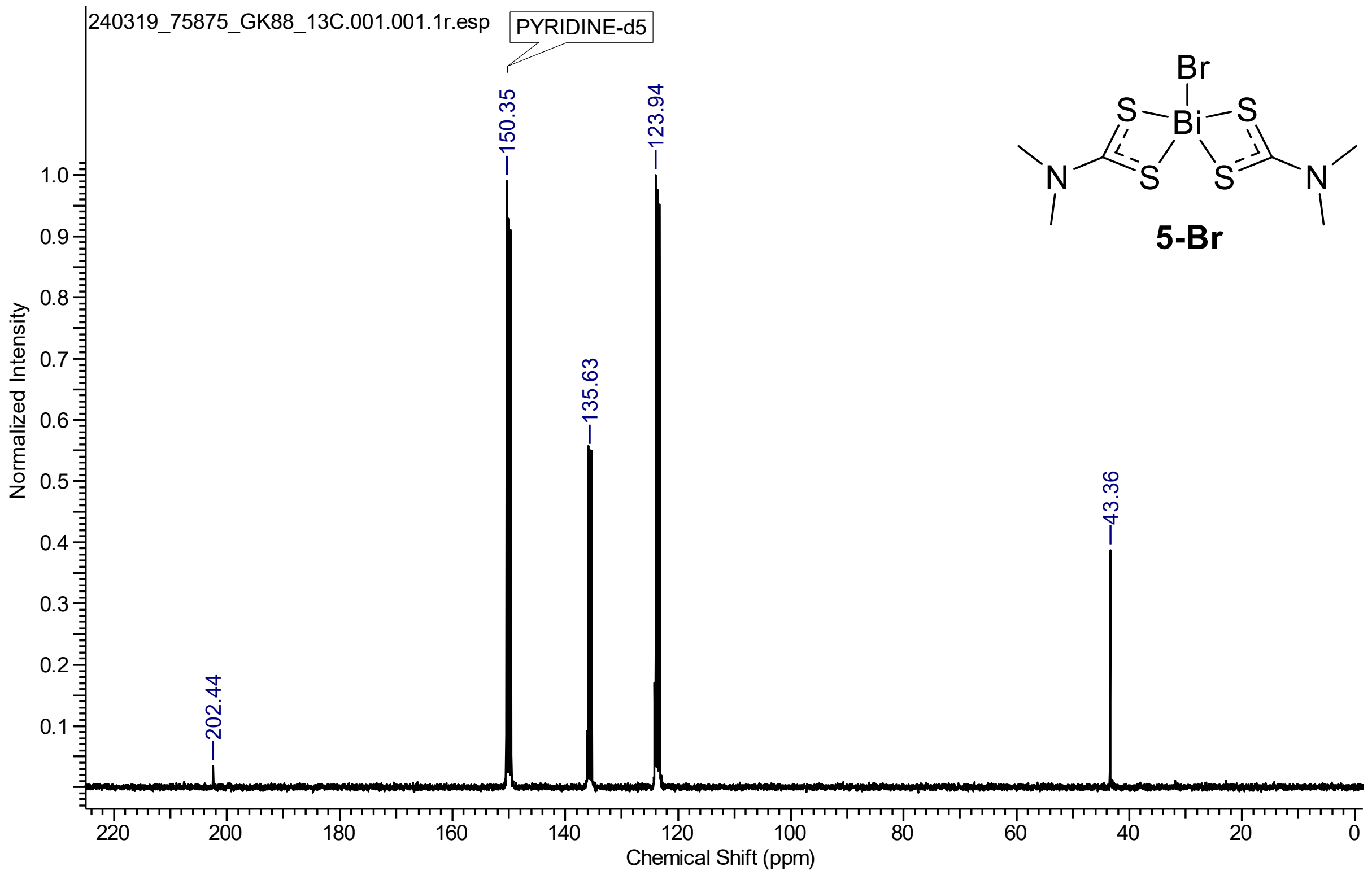

Figure S26: ^{13}C{^{1}H} NMR spectrum of **5-Br** (126 MHz, 298 K, Py-*d*$_5$).

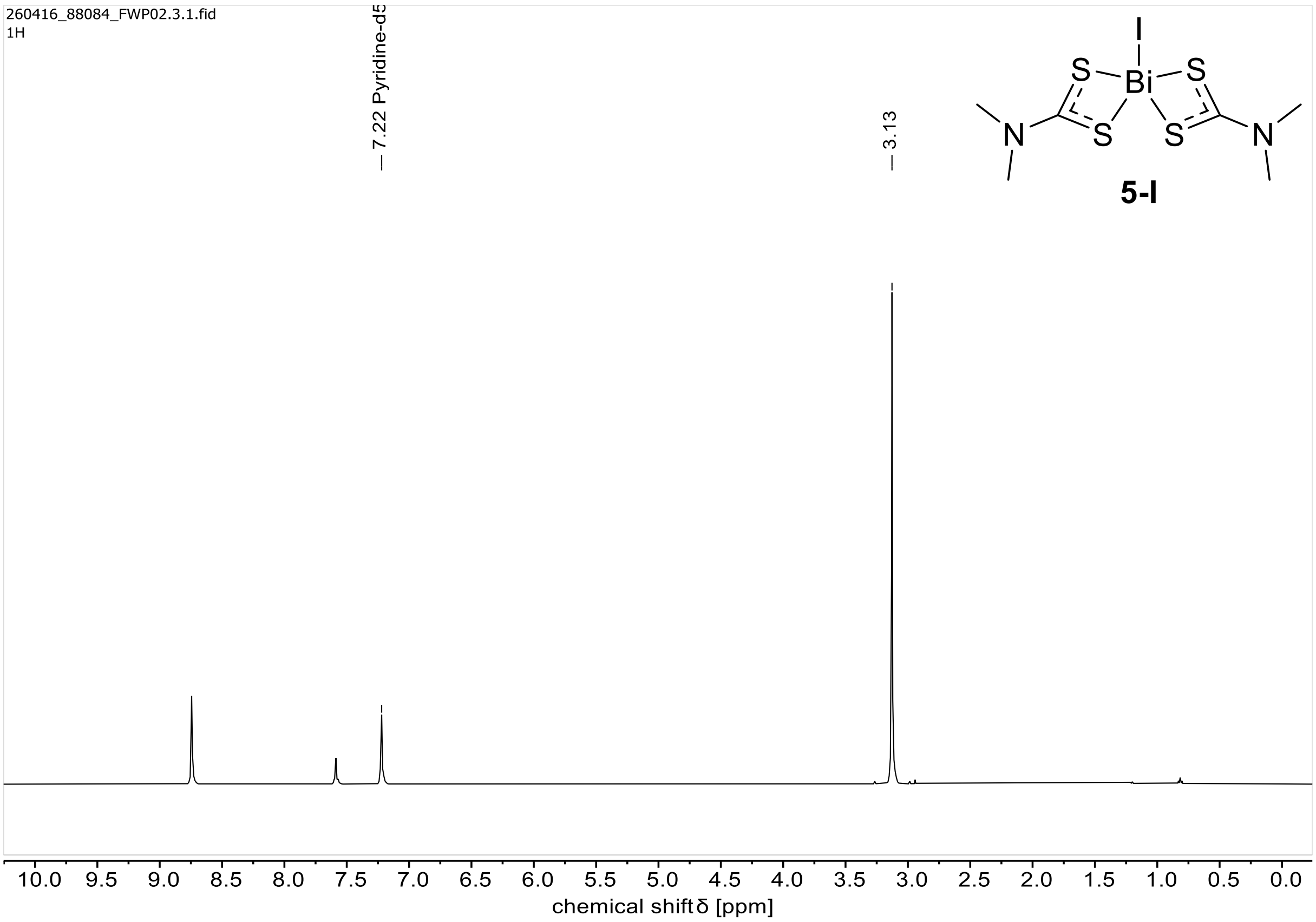

Figure S27: ^{1}H NMR spectrum of **5-I** (500 MHz, 298 K, Py-d_5).

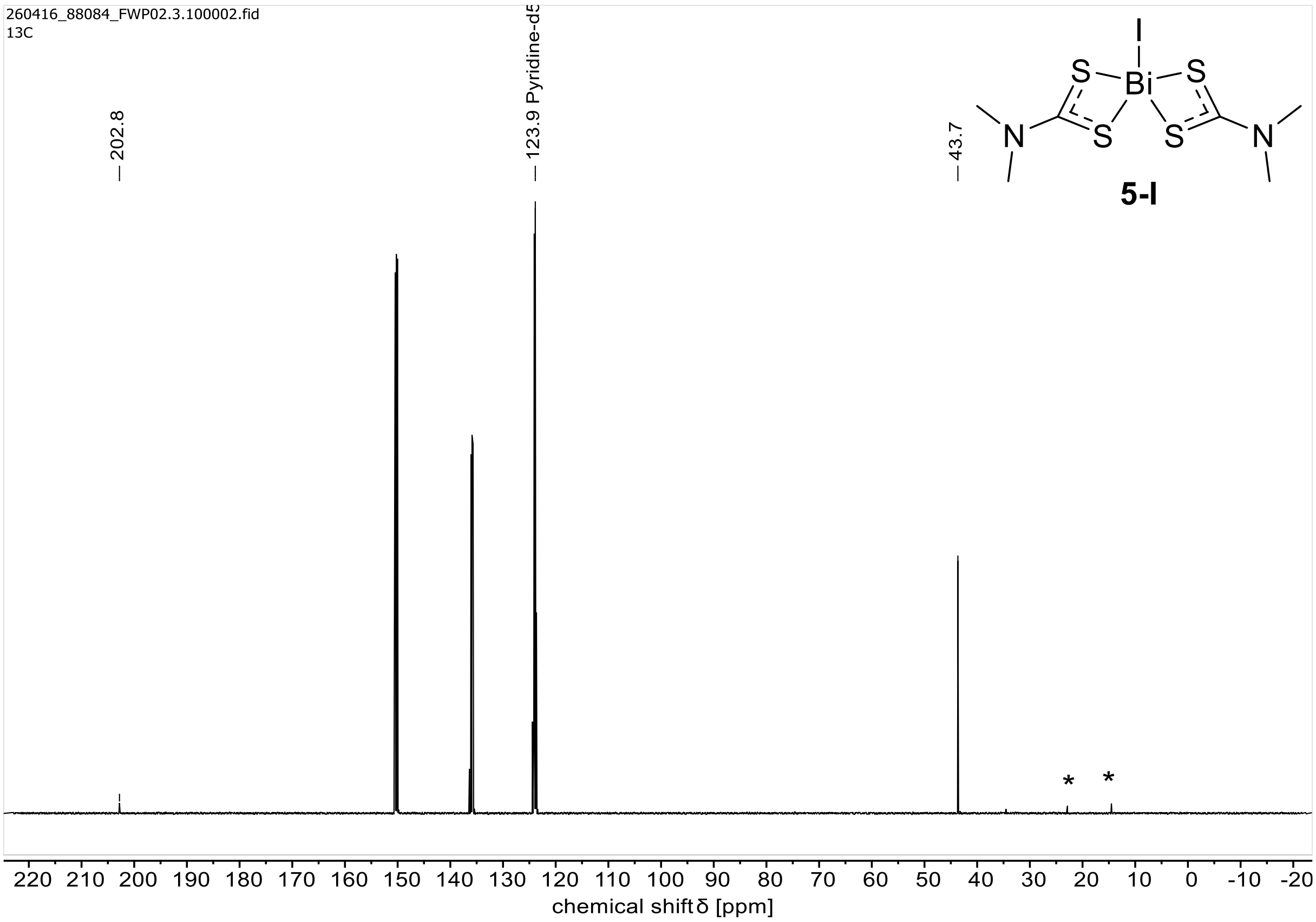

Figure S28: ^{13}C{^{1}H} NMR spectrum of **5-I** (126 MHz, 298 K, Py-d_5). * Residual amounts of ^{n}pentane.

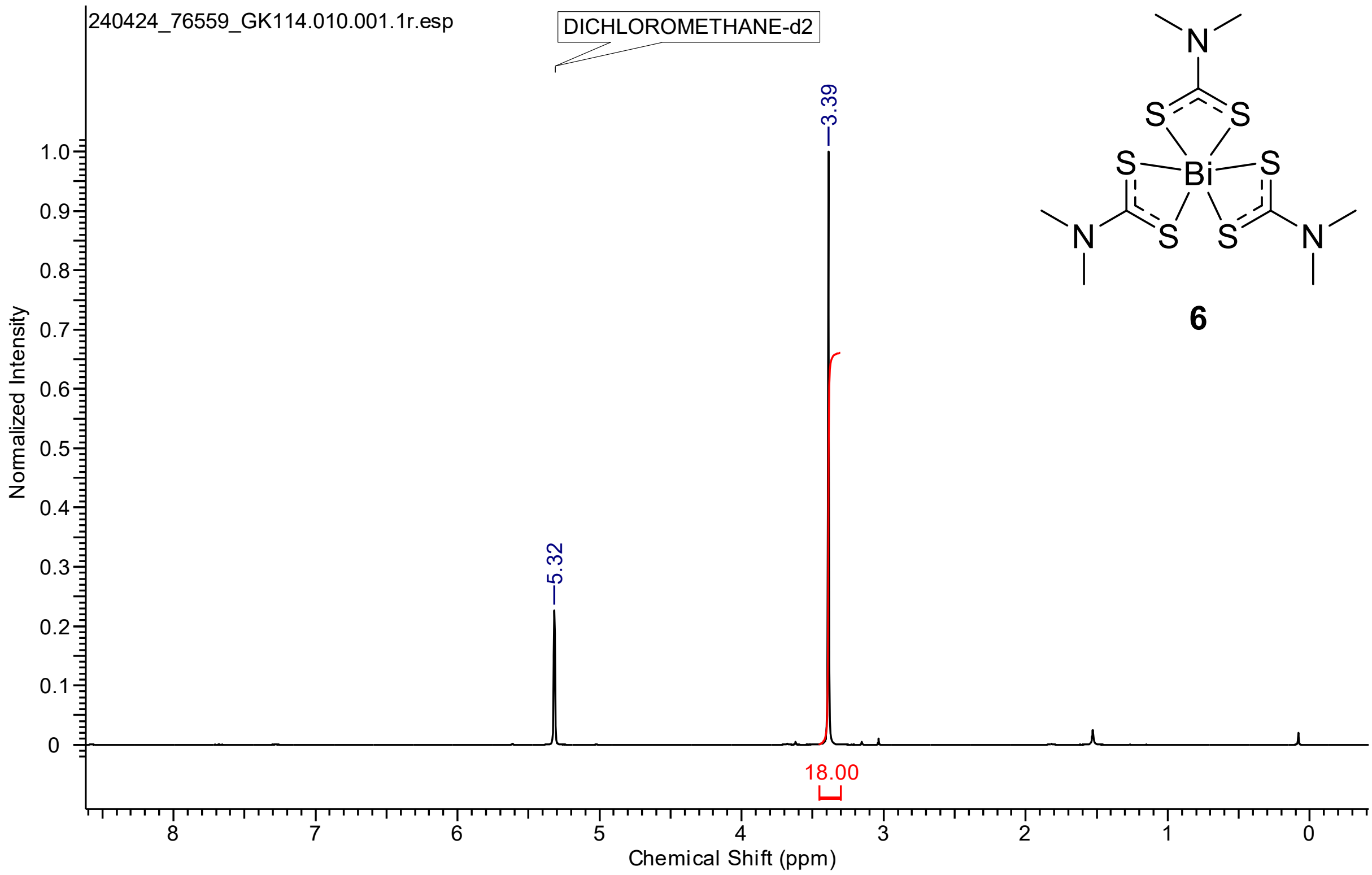

Figure S29: ^{1}H NMR spectrum of **6** (500 MHz, 298 K, CD_2Cl_2).

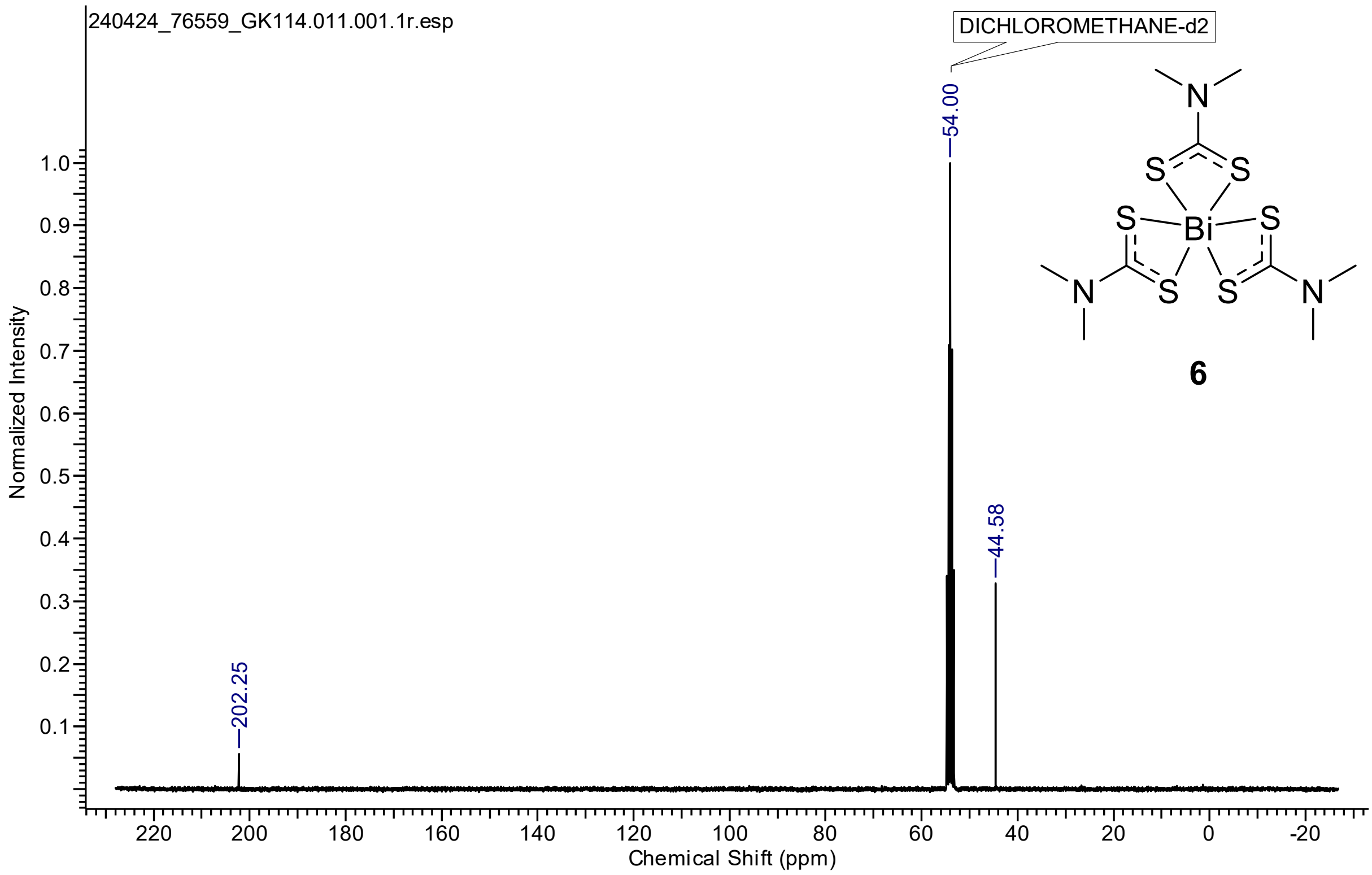

Figure S30: $^{13}C\{^{1}H\}$ NMR spectrum of **6** (126 MHz, 298 K, CD_2Cl_2).

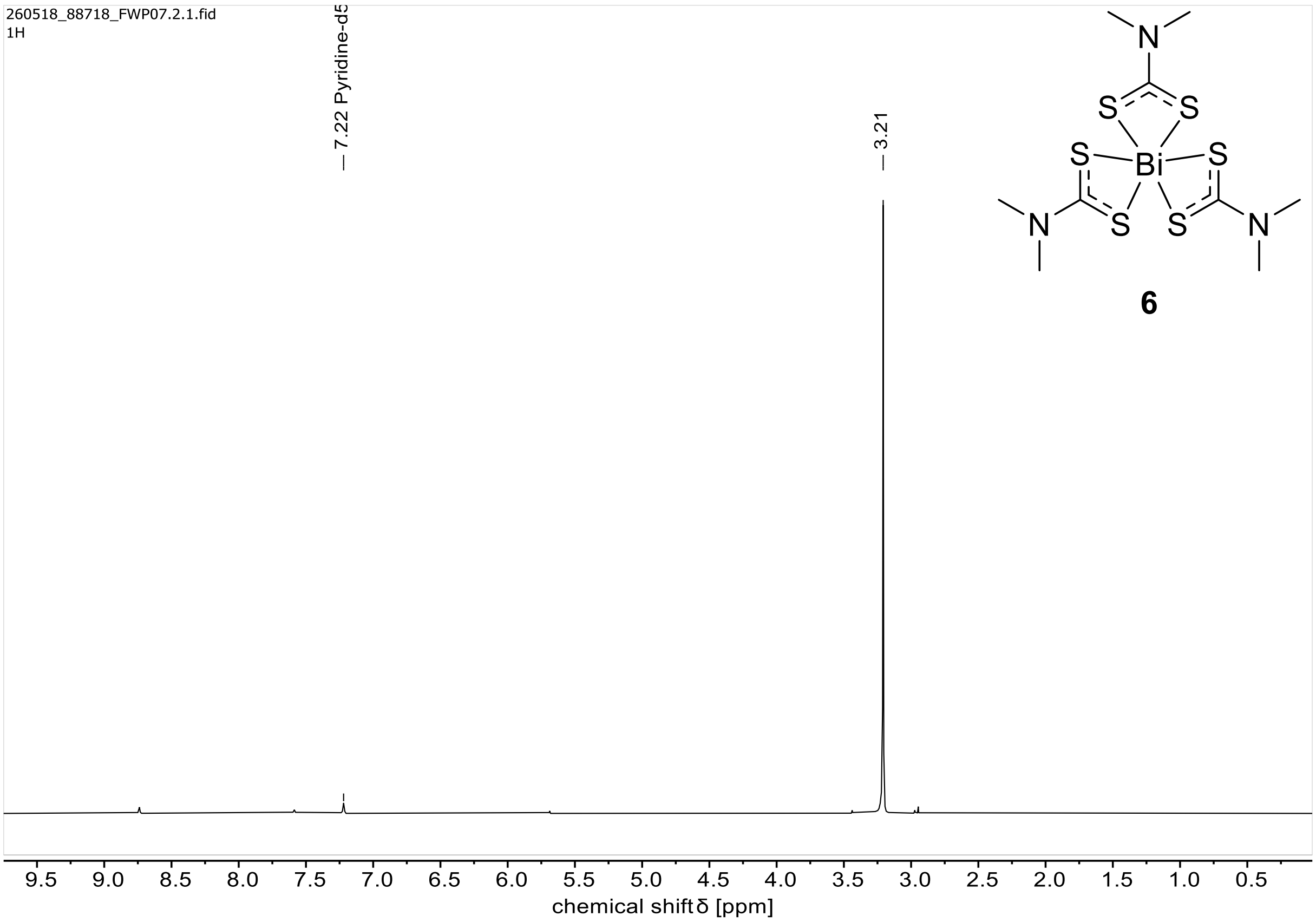

Figure S31: ^{1}H NMR spectrum of **6** (300 MHz, 298 K, Py-*d*$_5$).

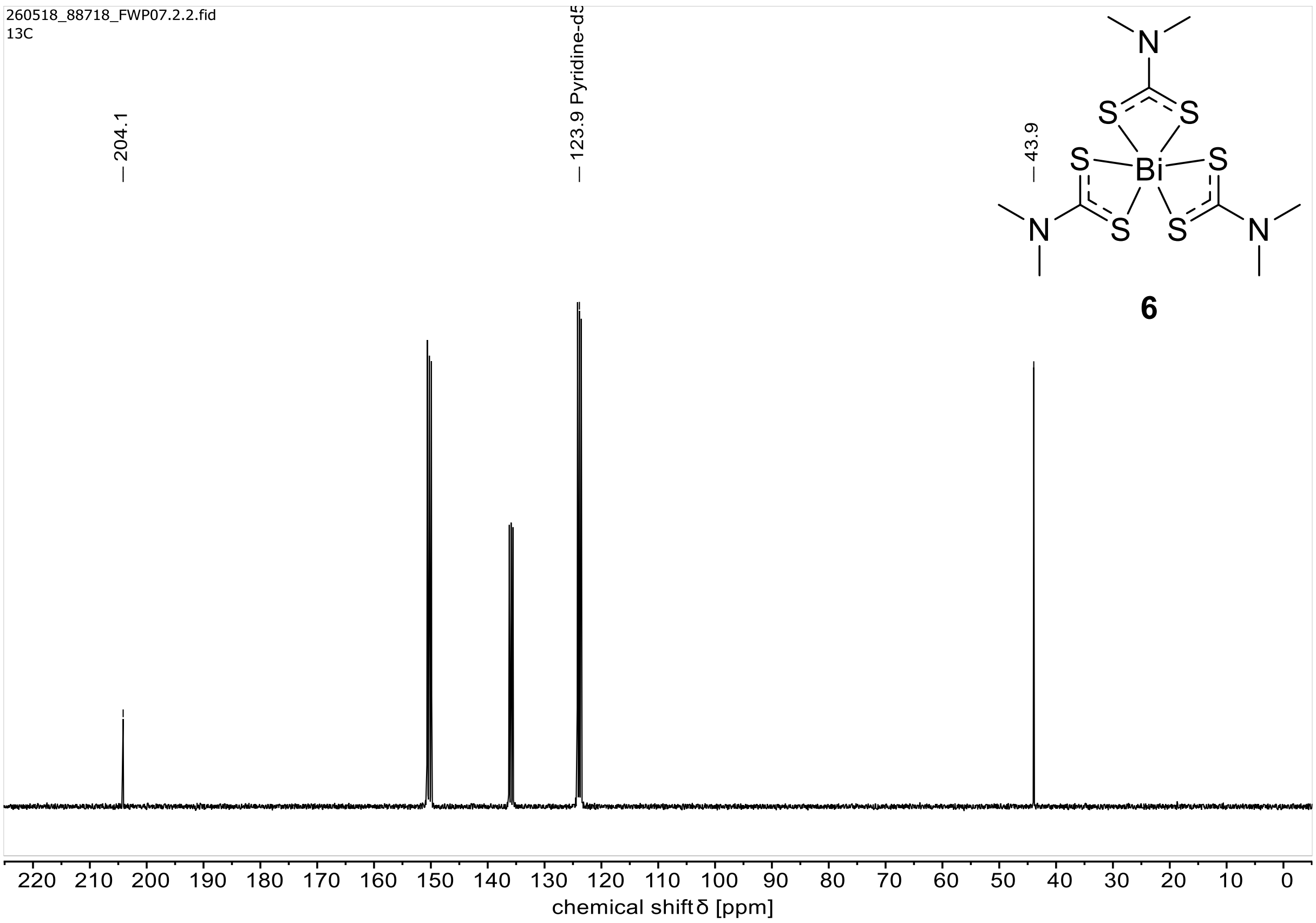

Figure S32: ^{13}C{^{1}H} NMR spectrum of **6** (75 MHz, 298 K, Py-*d*$_5$).

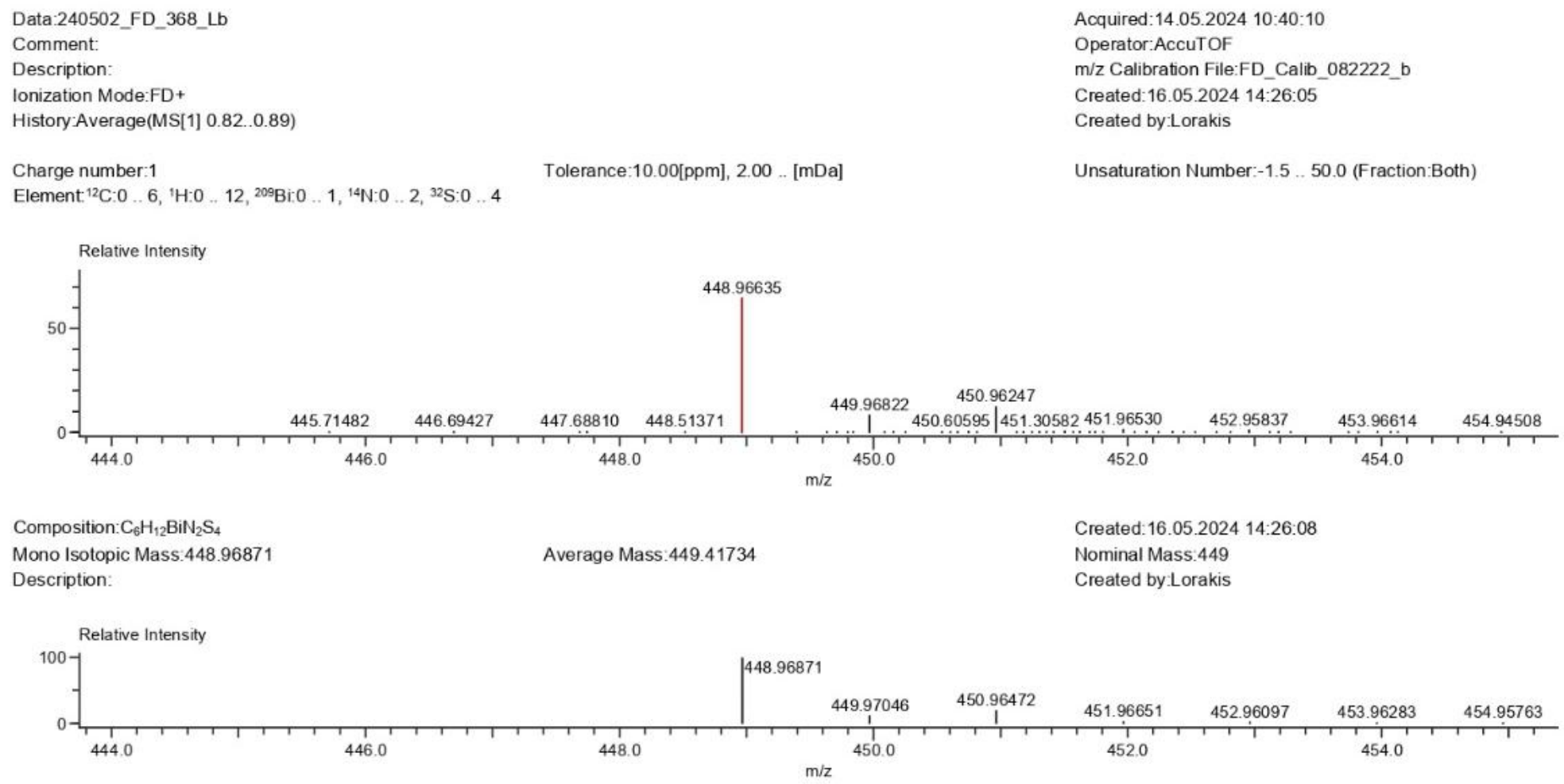

Mass	Intensity	Relative Intensity	Calc. Mass	Mass Difference [mDa]	Mass Difference [ppm]	Possible Formula	Unsaturation Number
448.96635	185052.47	64.33	448.96871	-2.36	-5.26	$^{12}C_6{}^1H_{12}{}^{209}Bi_1{}^{14}N_2{}^{32}S_4$	6.5

Figure S33: HR-MS (LIFDI, pos.) spectrum of **6**.

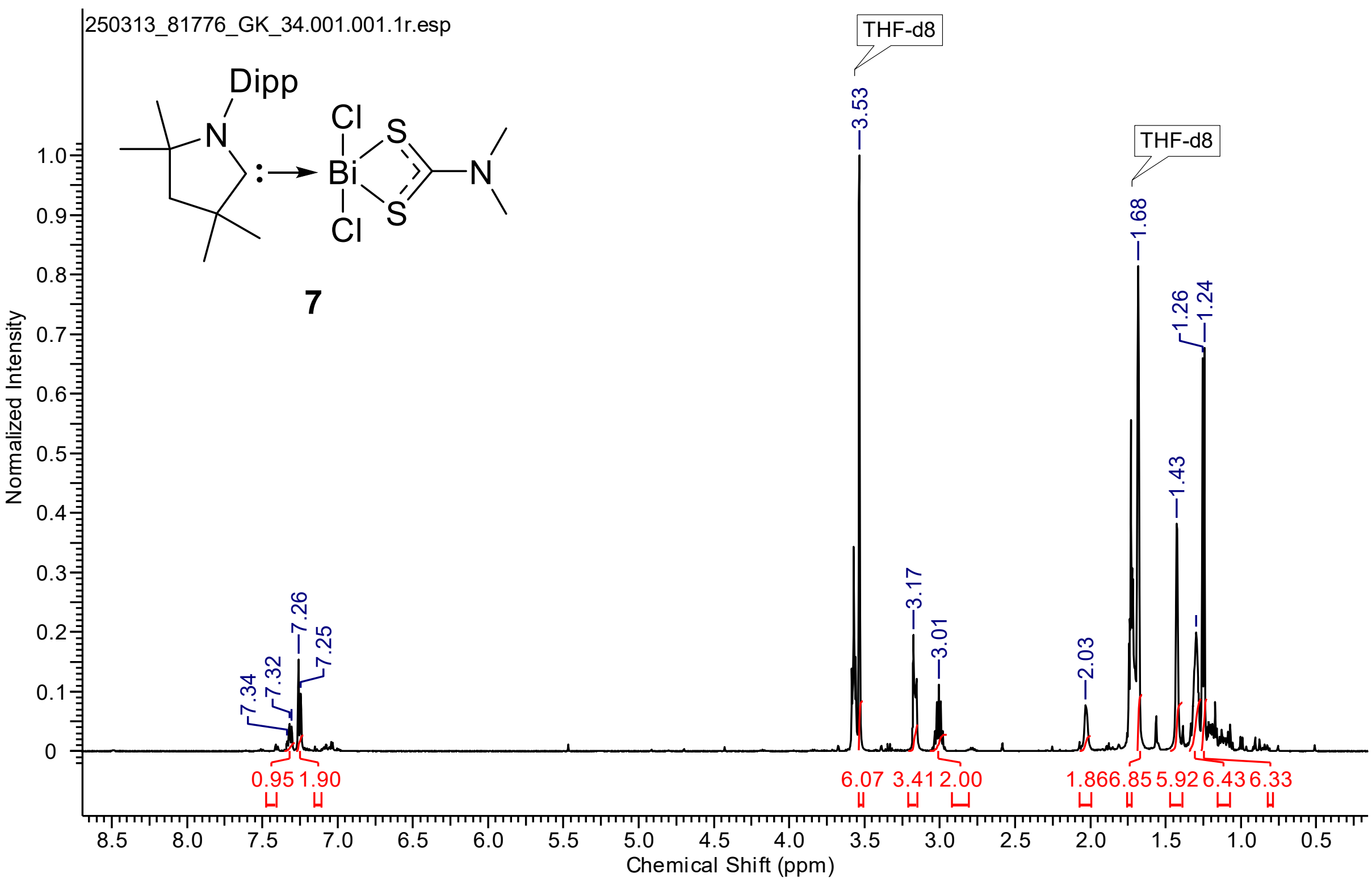

Figure S34: ^{1}H NMR spectrum of **7** (500 MHz, 298 K, THF-d_8).

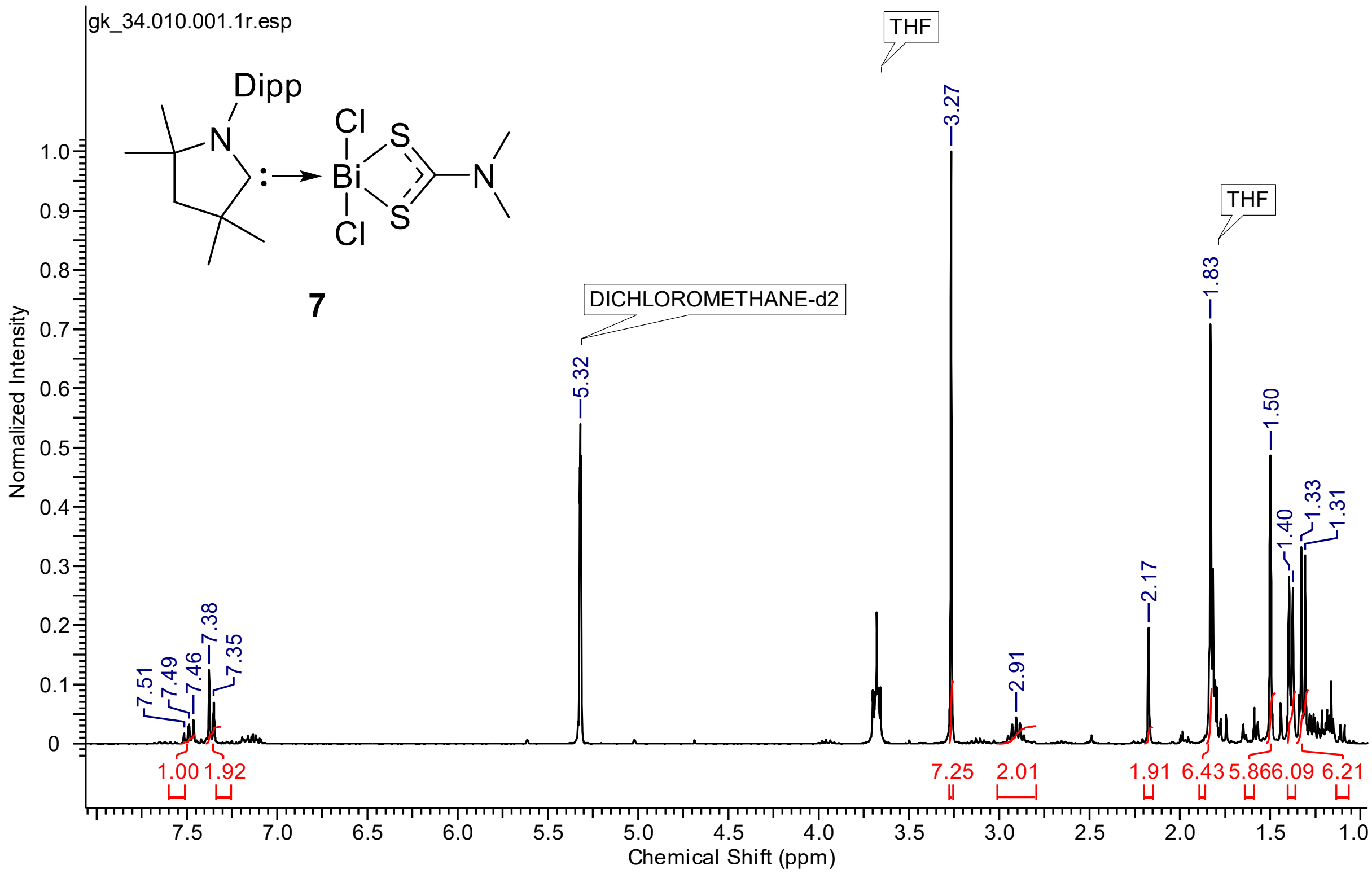

Figure S35: ^{1}H NMR spectrum of **7** (500 MHz, 298 K, CD_2Cl_2).

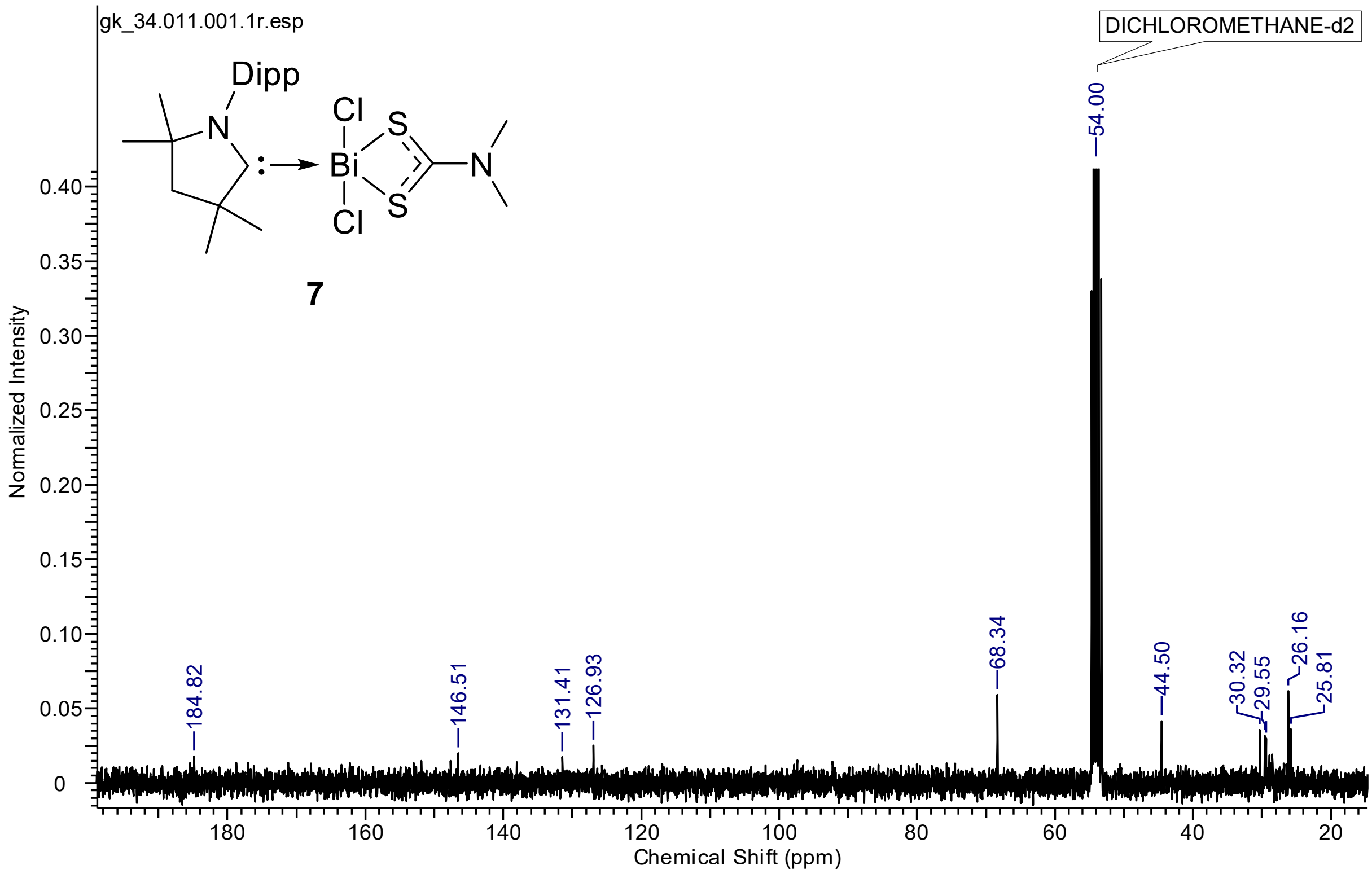

Figure S36: $^{13}C\{^{1}H\}$ NMR spectrum of **7** (126 MHz, 298 K, CD_2Cl_2).

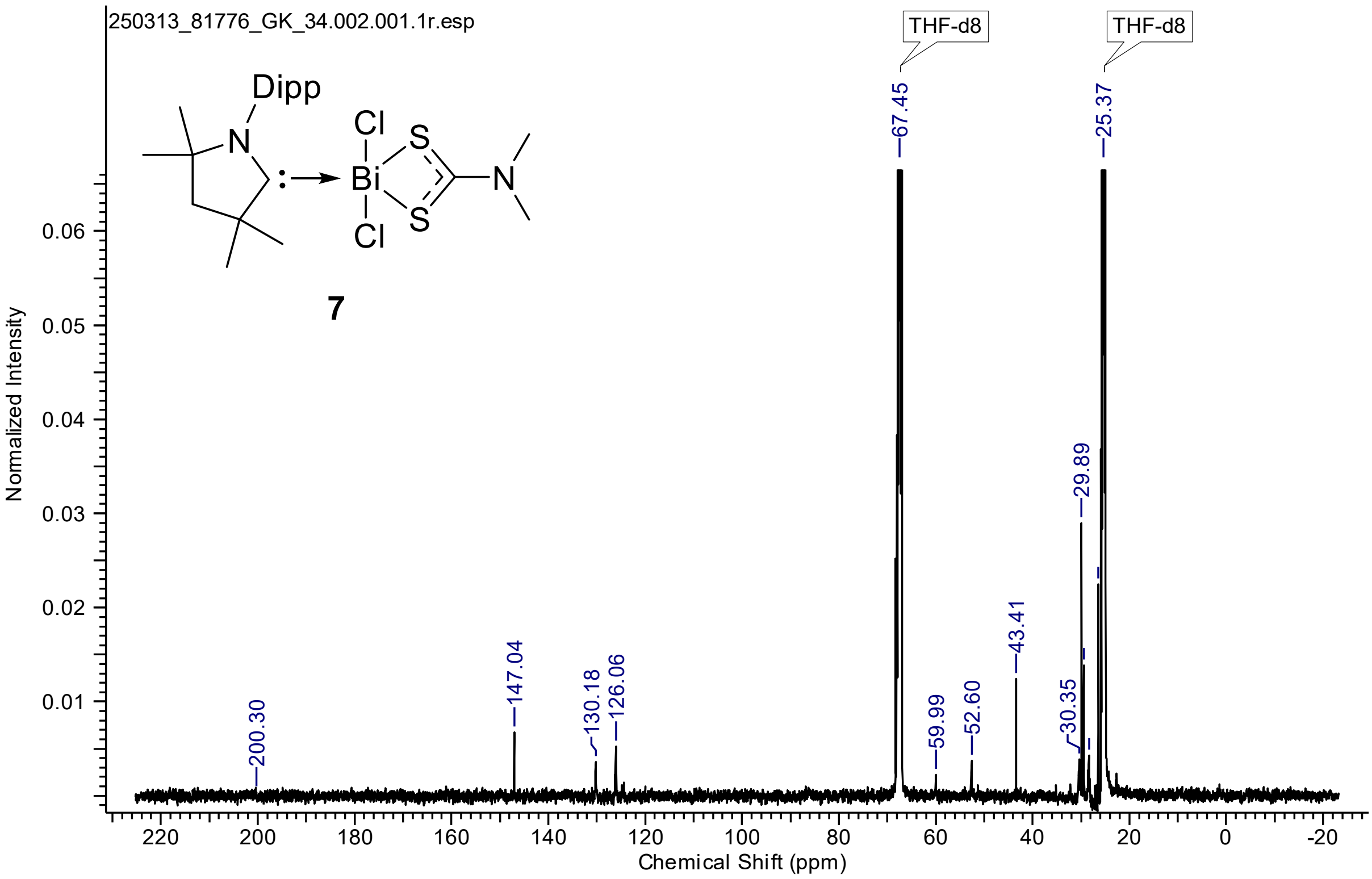

Figure S37: $^{13}C\{^{1}H\}$ NMR spectrum of **7** (126 MHz, 298 K, THF-d_8).

Data:240510_FD_380_Lb
Comment:
Description:
Ionization Mode:FD+
History:Average(MS[1] 0.91..0.96)

Acquired:16.05.2024 10:00:41
Operator:AccuTOF
m/z Calibration File:FD_Calib_082222_b
Created:21.05.2024 11:12:06
Created by:Lorakis

Charge number:1
Tolerance:5.00[ppm], 2.00 .. [mDa]
Unsaturation Number:-1.5 .. 50.0 (Fraction:Both)
Element:^{12}C:0 .. 21, ^{1}H:0 .. 33, ^{209}Bi:1 .. 1, ^{35}Cl:0 .. 10, ^{14}N:0 .. 1, ^{32}S:0 .. 2

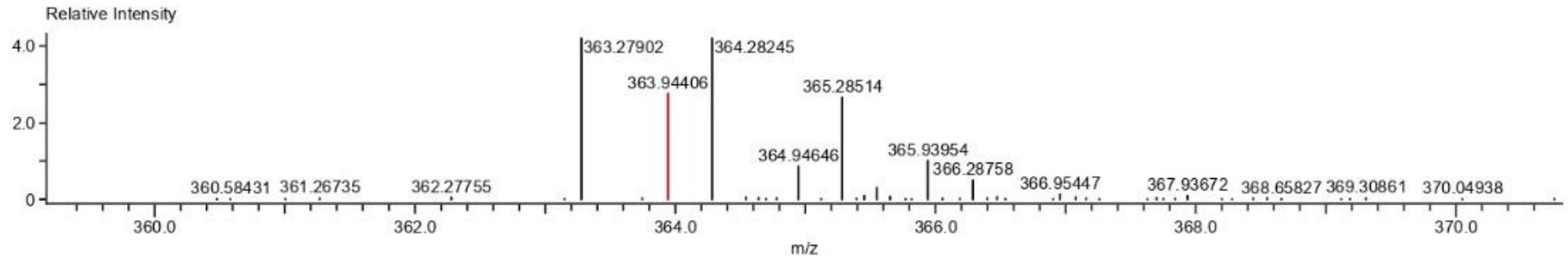

Composition:$C_3H_6BiClNS_2$
Mono Isotopic Mass:363.94340
Average Mass:364.65156
Description:

Created:21.05.2024 11:12:16
Nominal Mass:364
Created by:Lorakis

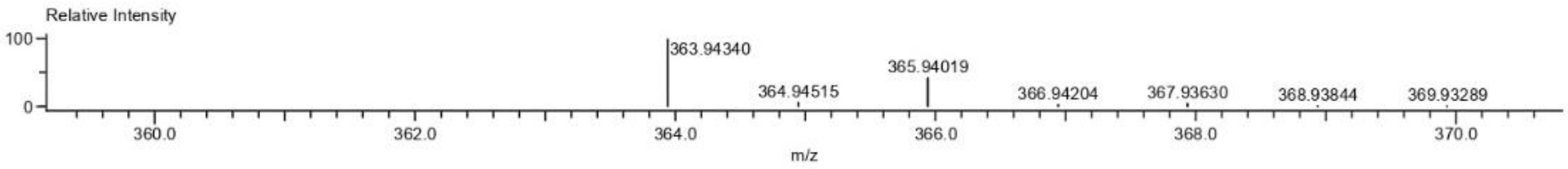

Mass	Intensity	Relative Intensity	Calc. Mass	Mass Difference [mDa]	Mass Difference [ppm]	Possible Formula	Unsaturation Number
363.94406	17728.71	2.75	363.94340	0.66	1.80	$^{12}C_3{}^{1}H_6{}^{209}Bi_1{}^{35}Cl_1{}^{14}N_1{}^{32}S_2$	3.5

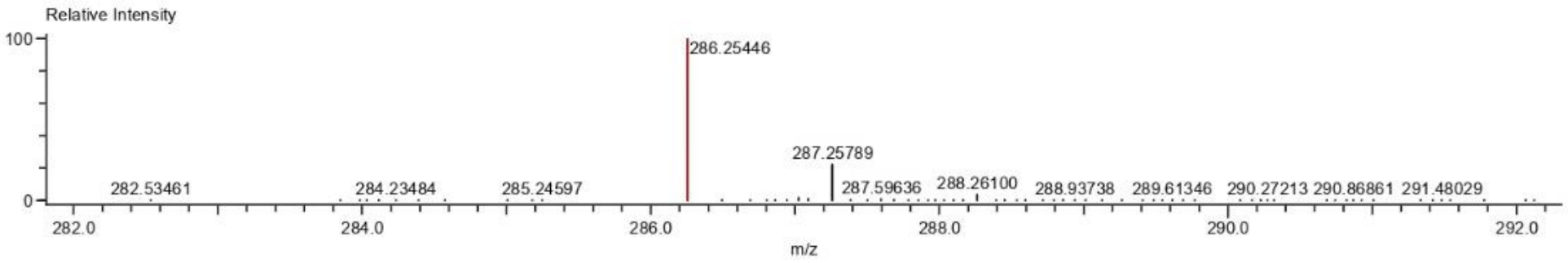

Composition:$C_{20}H_{32}N$
Mono Isotopic Mass:286.25347
Average Mass:286.47482
Description:

Created:21.05.2024 11:00:37
Nominal Mass:286
Created by:Lorakis

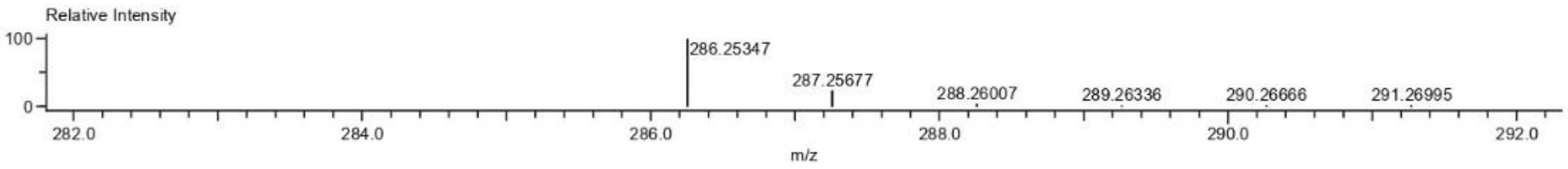

Mass	Intensity	Relative Intensity	Calc. Mass	Mass Difference [mDa]	Mass Difference [ppm]	Possible Formula	Unsaturation Number
286.25446	414964.08	100.00	286.25347	0.99	3.46	$^{12}C_{20}{}^{1}H_{32}{}^{14}N_1$	5.5

O:\Q Exactive Plus\...\250313_FD_629_Lb   17.03.2025 09:57:34

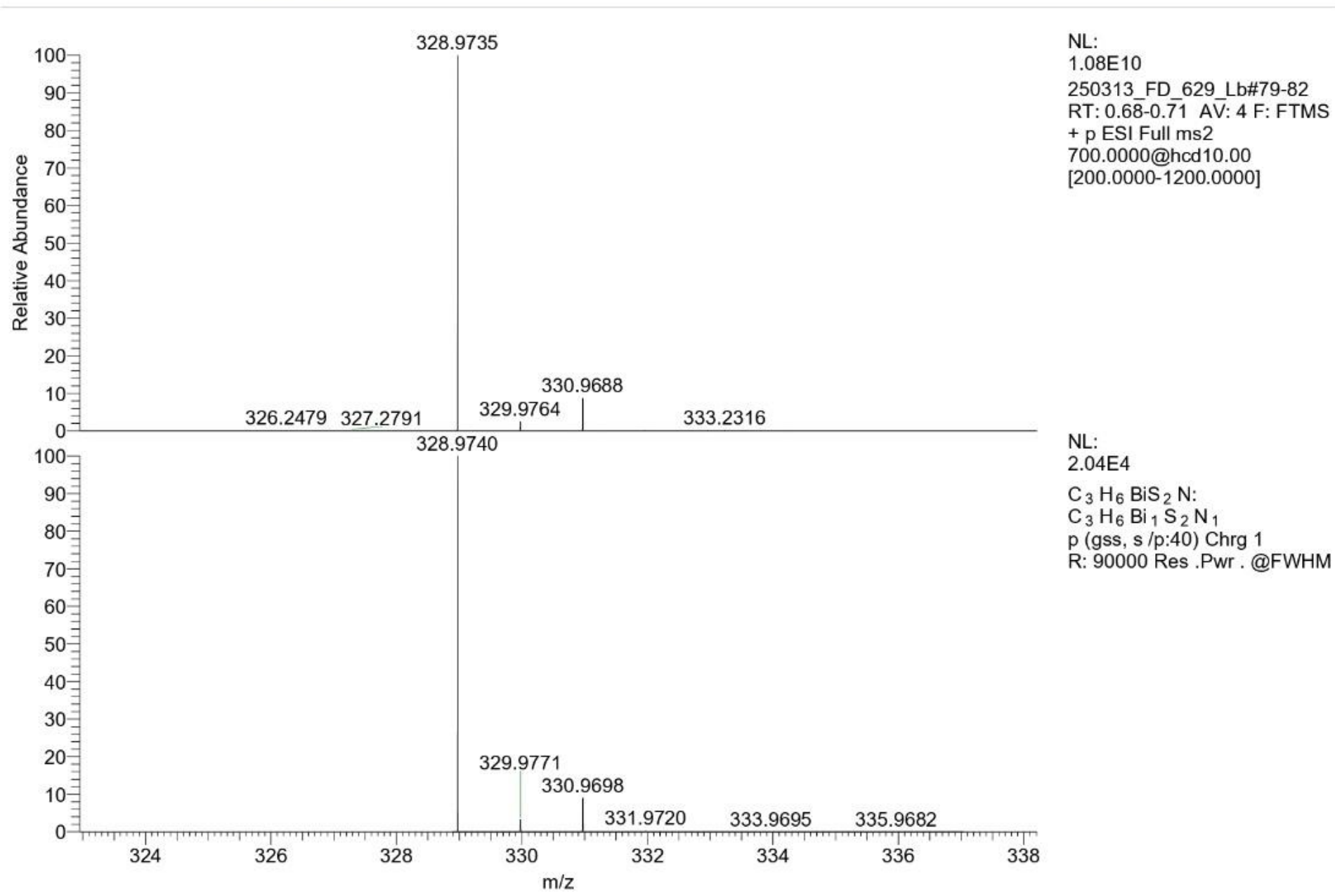

Figure S38: HR-MS (LIFDI, pos.) spectra of **7**.

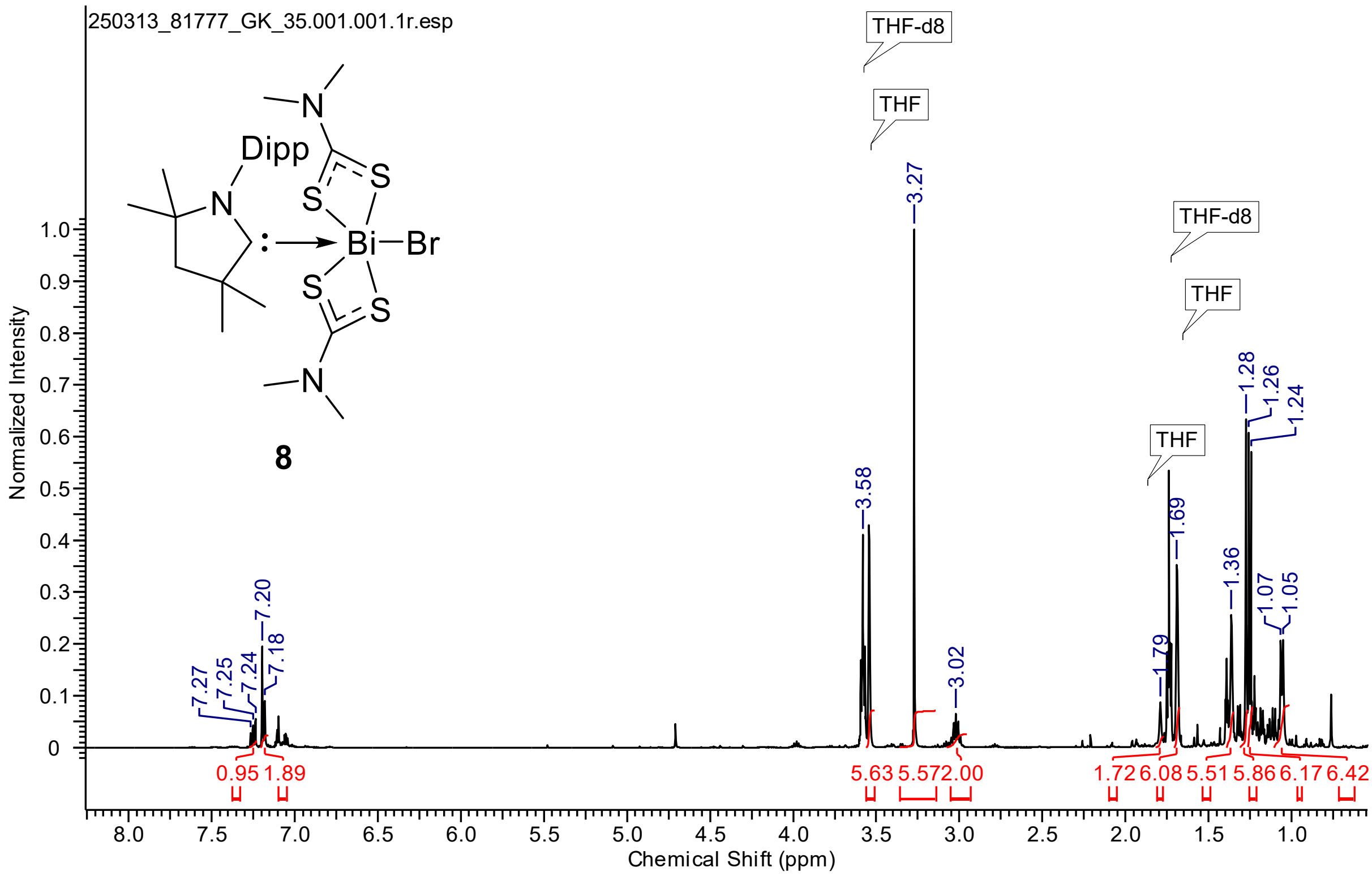

Figure S39: ^{1}H NMR spectrum of **8** (500 MHz, 298 K, THF-d_8).

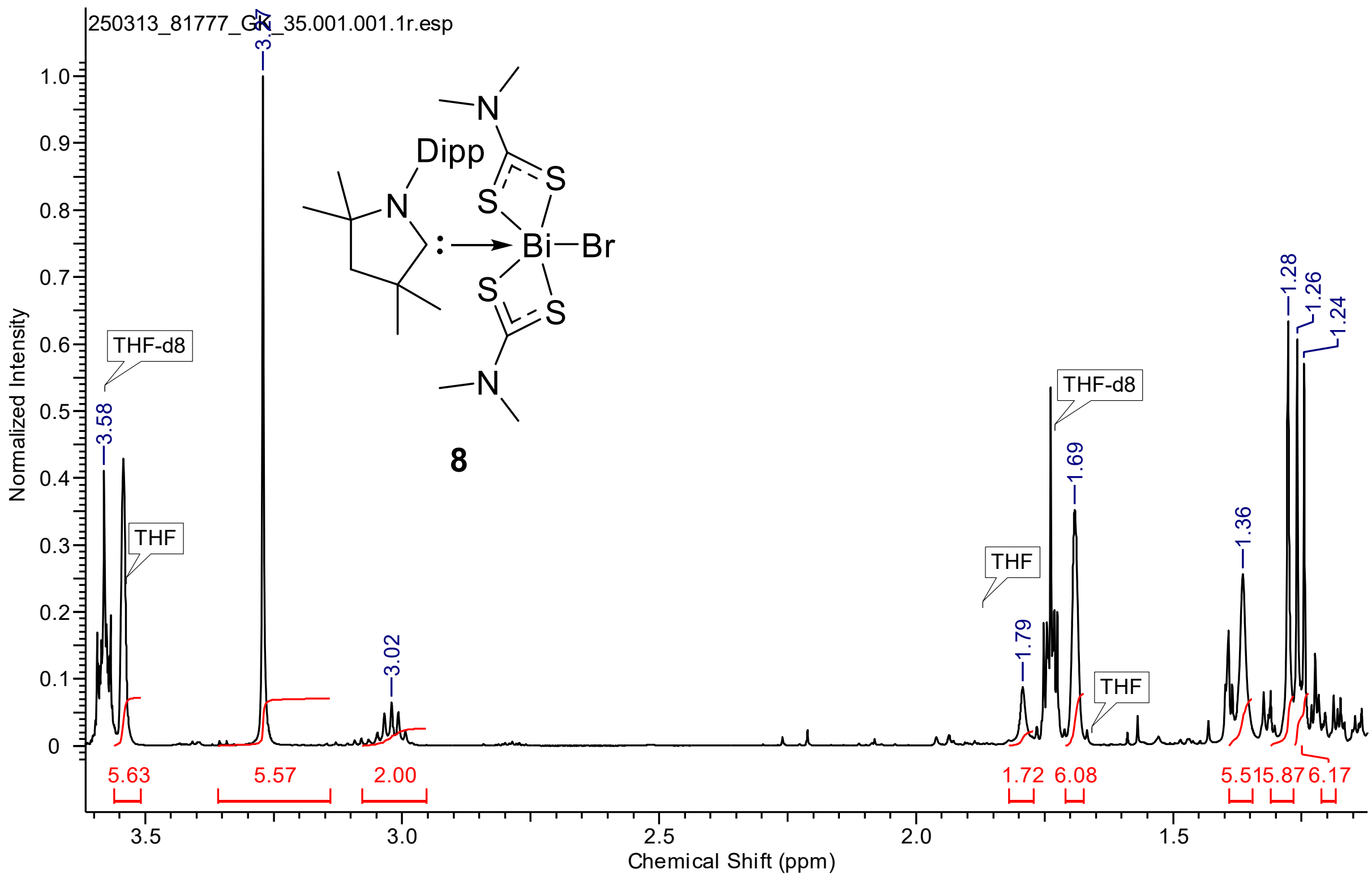

Figure S40: Close-up of the ^{1}H NMR spectrum of **8**.

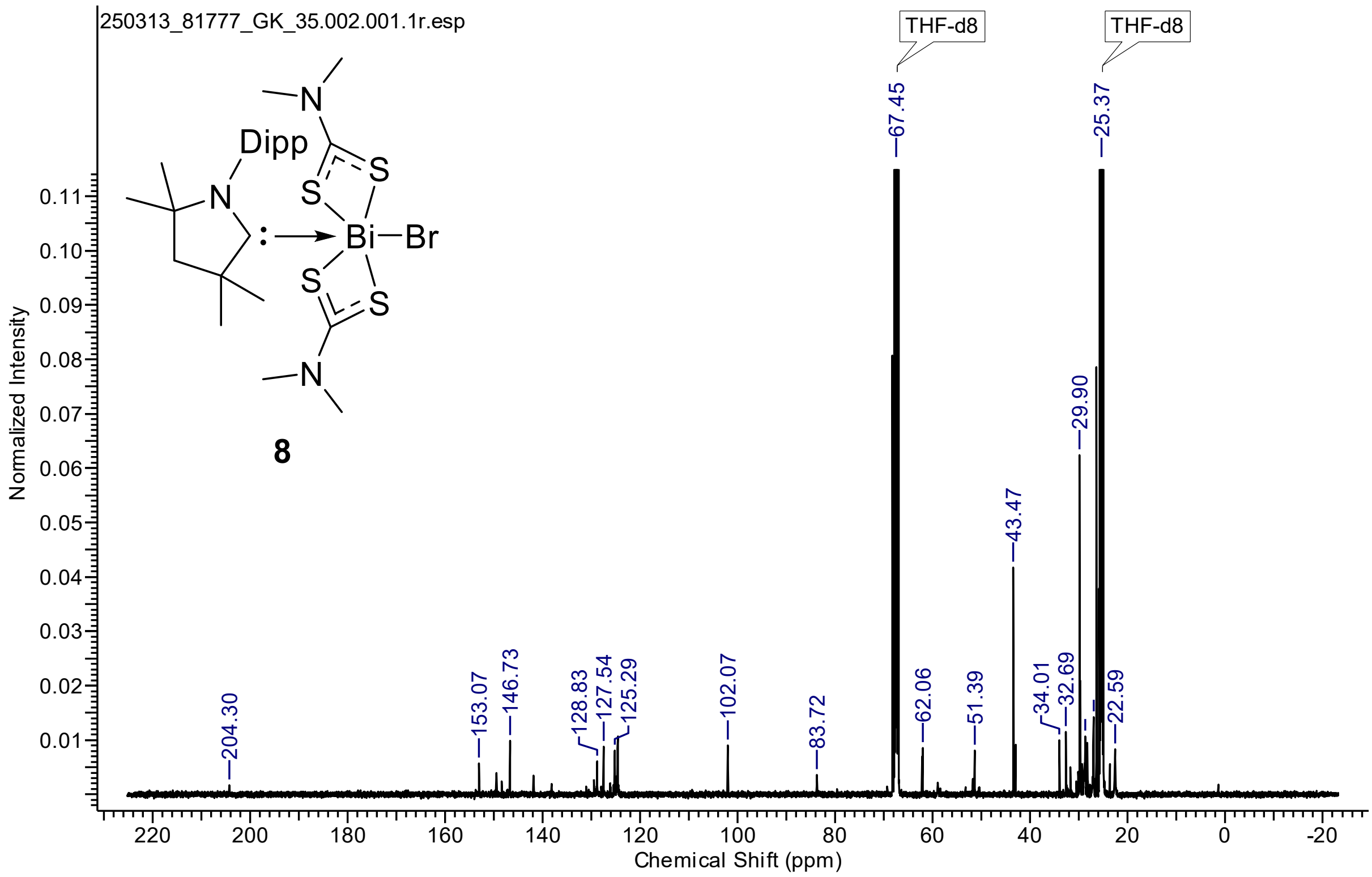

Figure S41: $^{13}C\{^{1}H\}$ NMR spectrum of **8** (126 MHz, 298 K, THF-d_8).

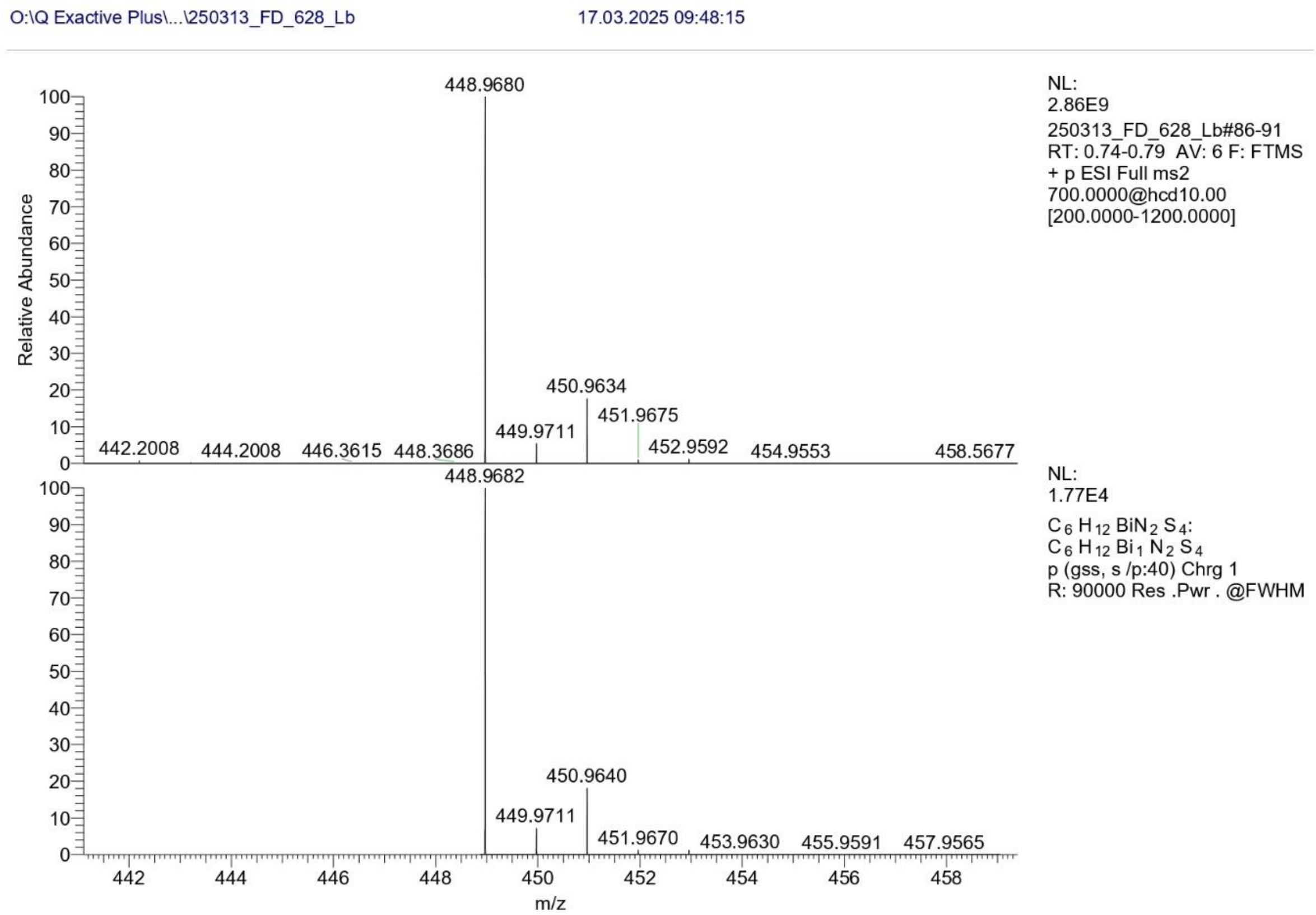

Figure S42: HR-MS (LIFDI, pos.) spectrum of **8**.

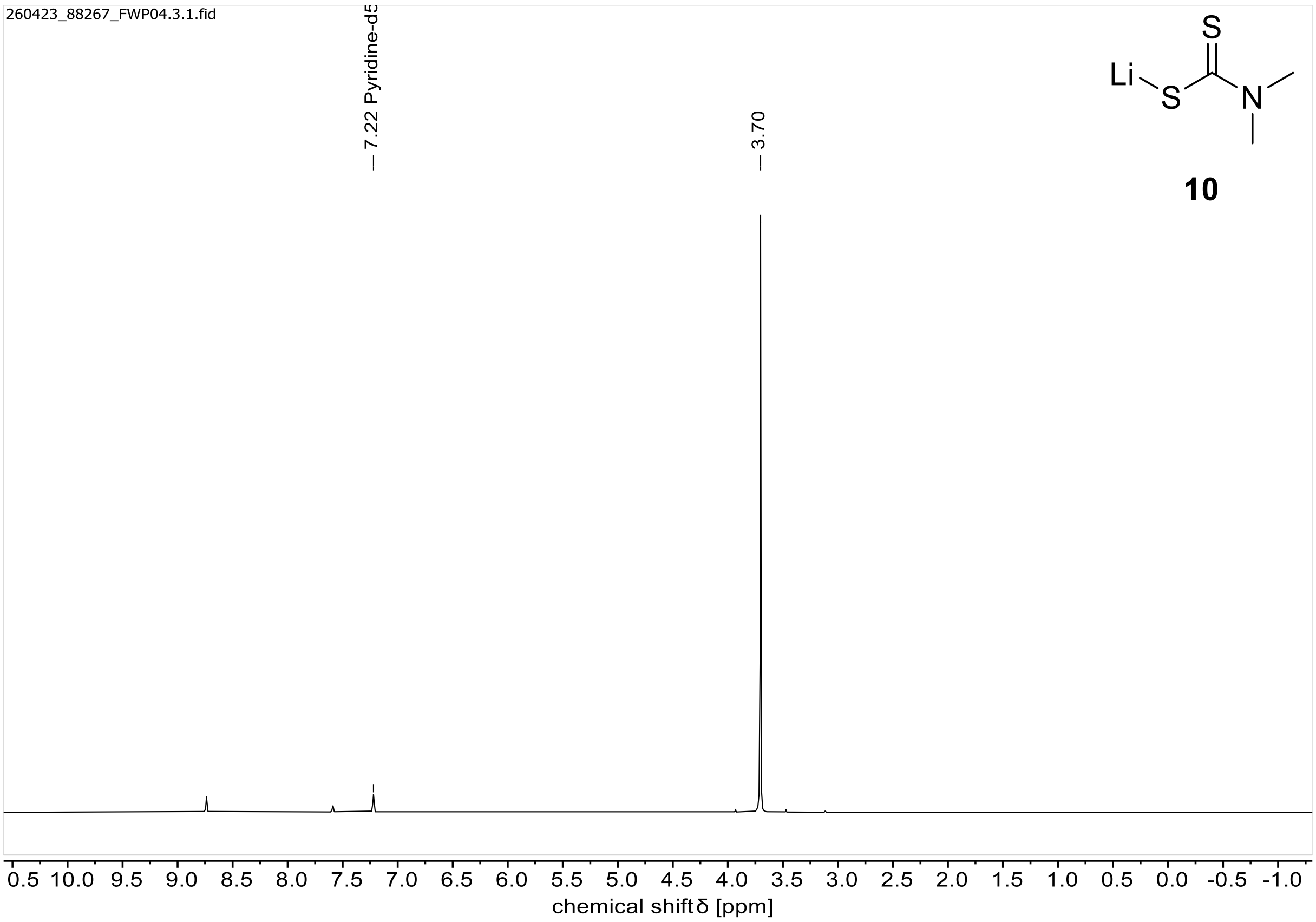

Figure S43: ^{1}H NMR spectrum of **10** (300 MHz, 298 K, Py-d_5).

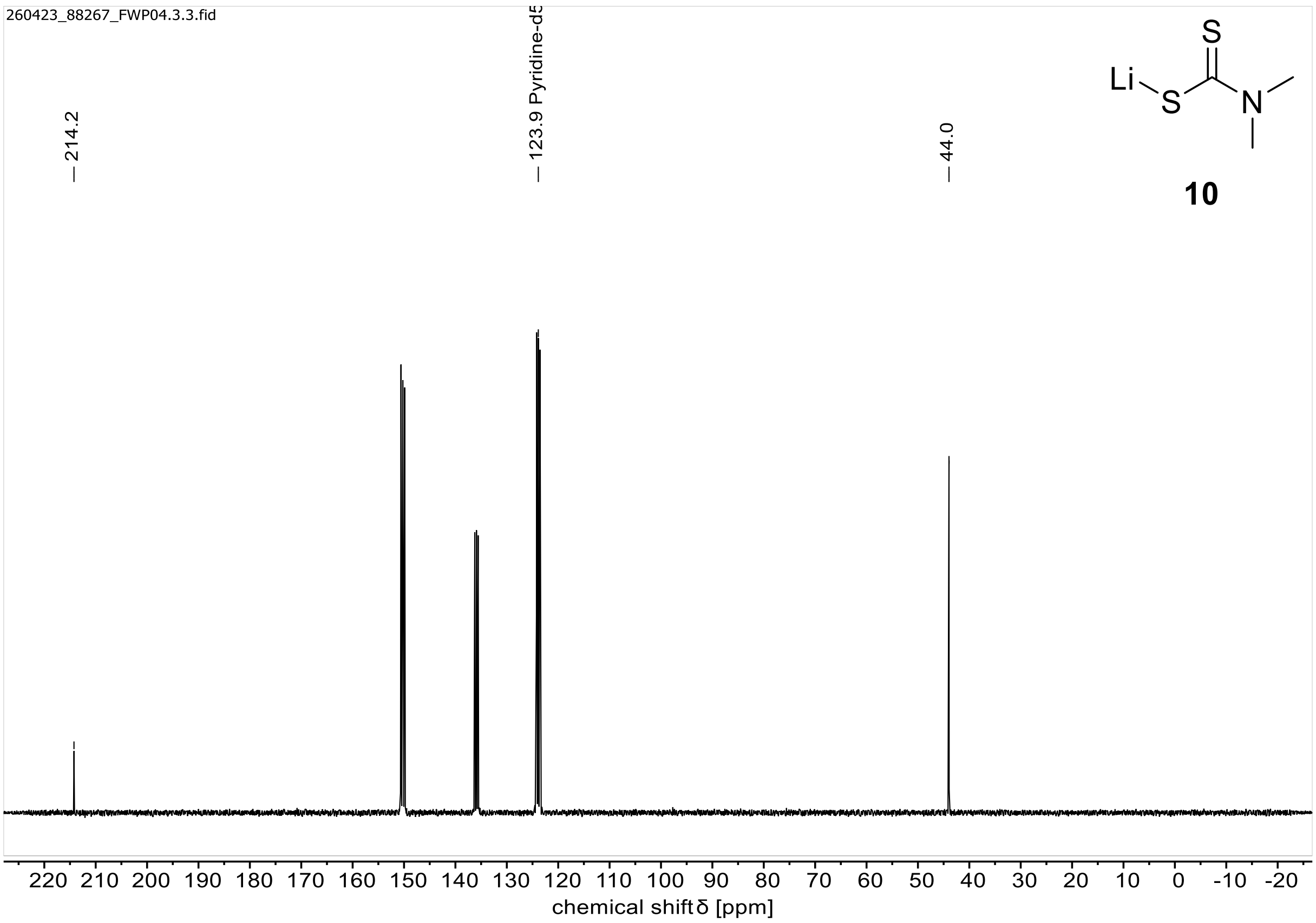

Figure S44: ^{13}C{^{1}H} NMR spectrum of **10** (75 MHz, 298 K, Py-d_5).

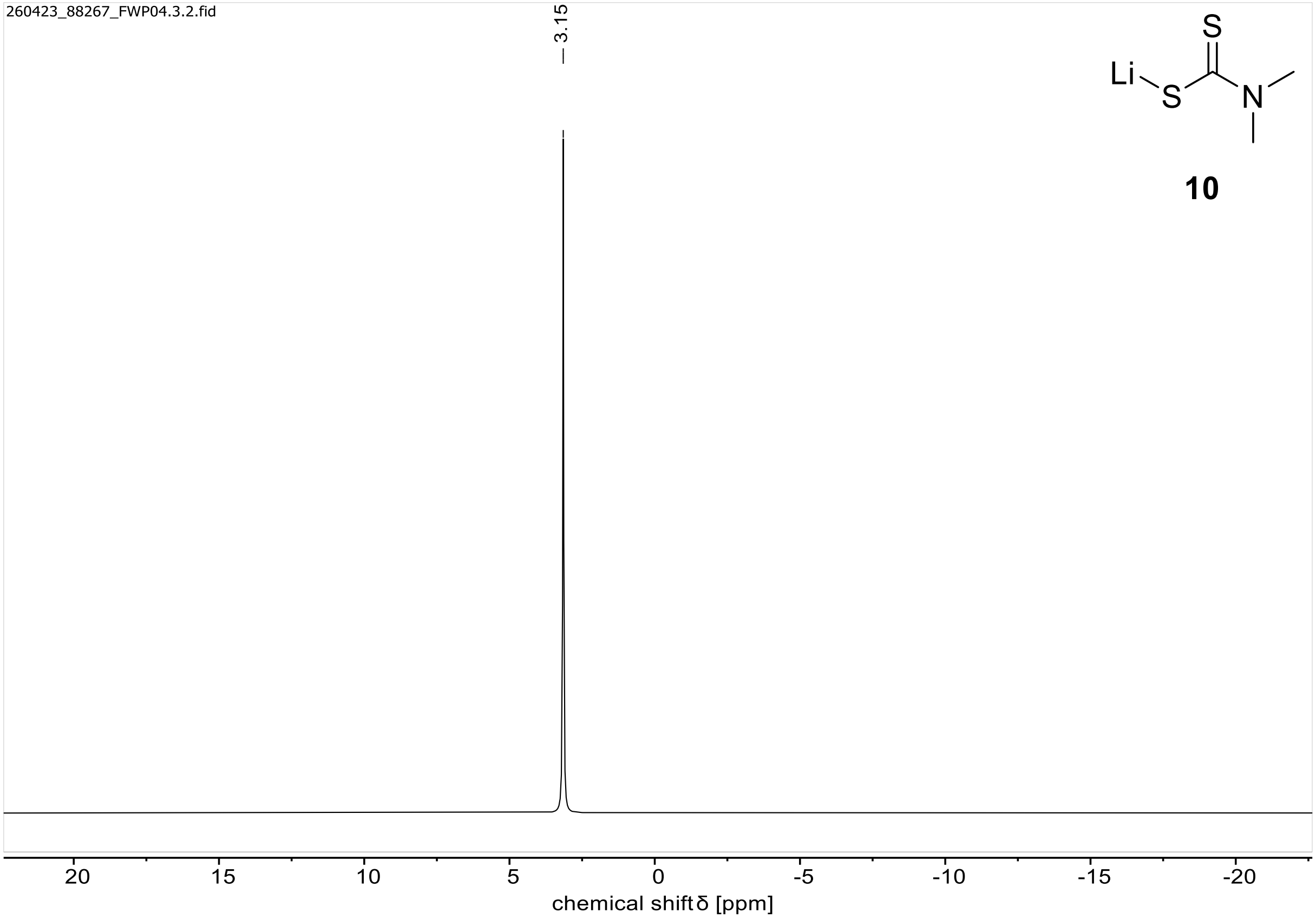

Figure S45: ^{7}Li NMR spectrum of **10** (117 MHz, 298 K, Py-d_5).

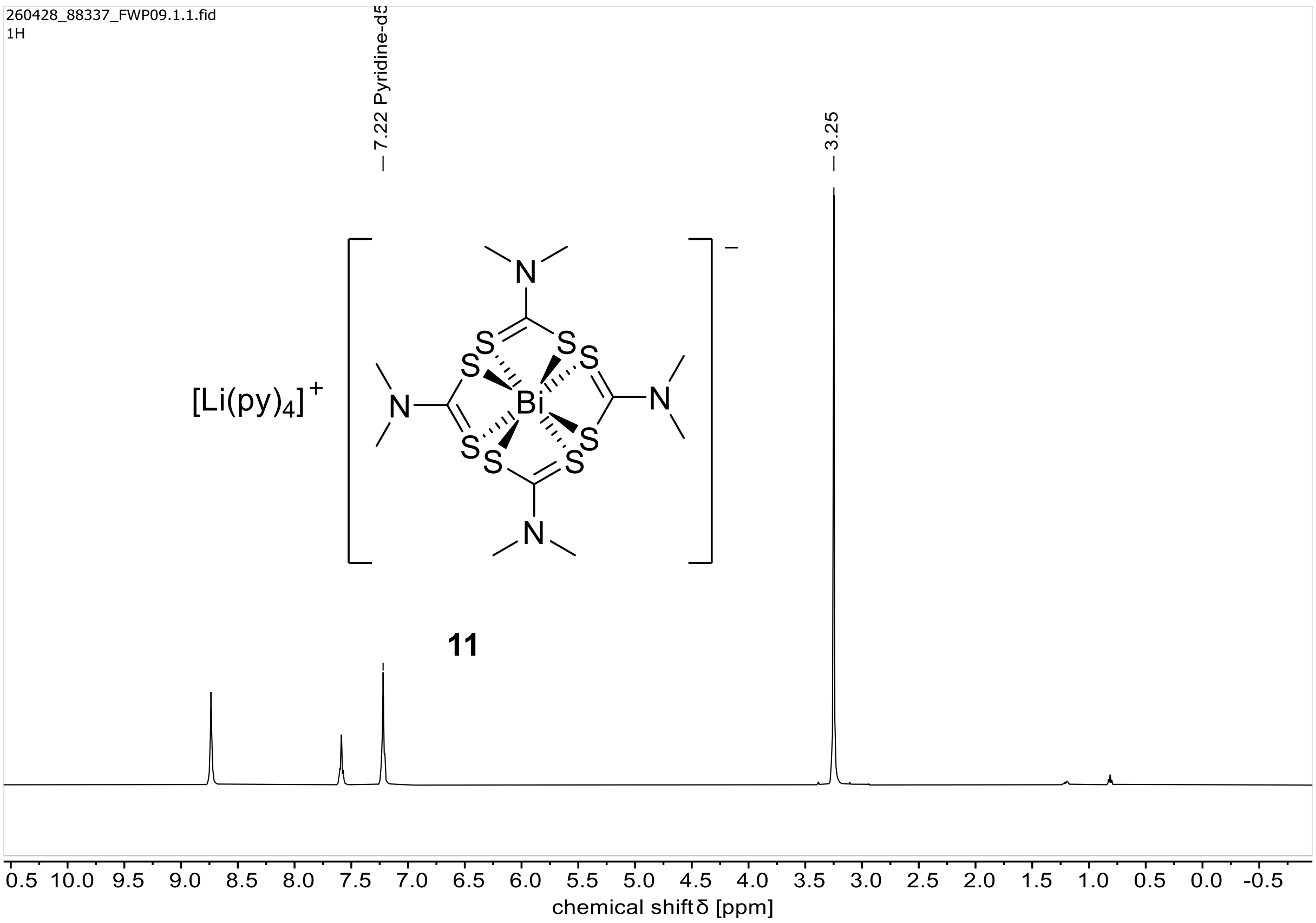

Figure S46: ^{1}H NMR spectrum of **11** (500 MHz, 298 K, Py-d_5).

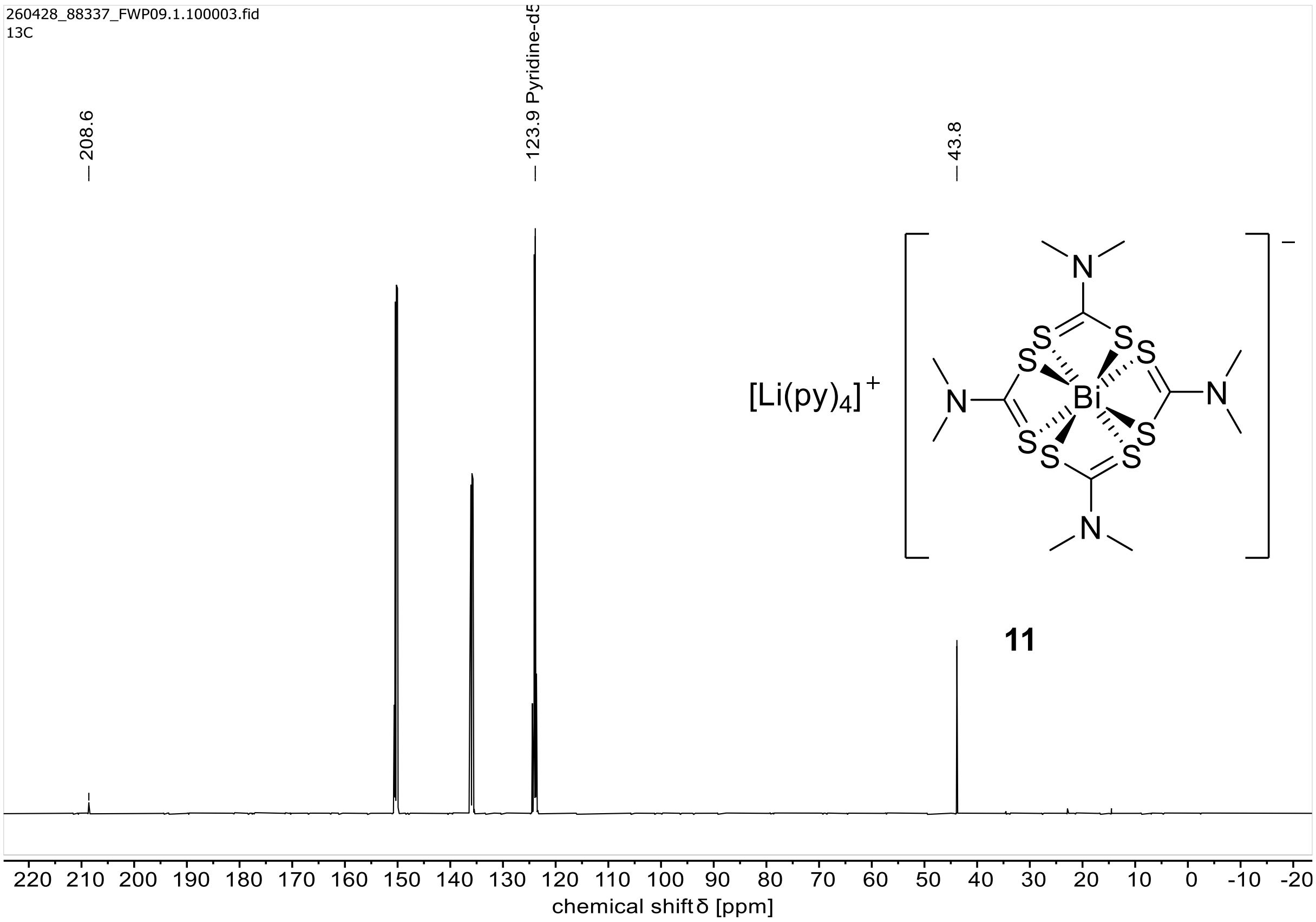

Figure S47: $^{13}C\{^{1}H\}$ NMR spectrum of **11** (126 MHz, 298 K, Py-d_5).

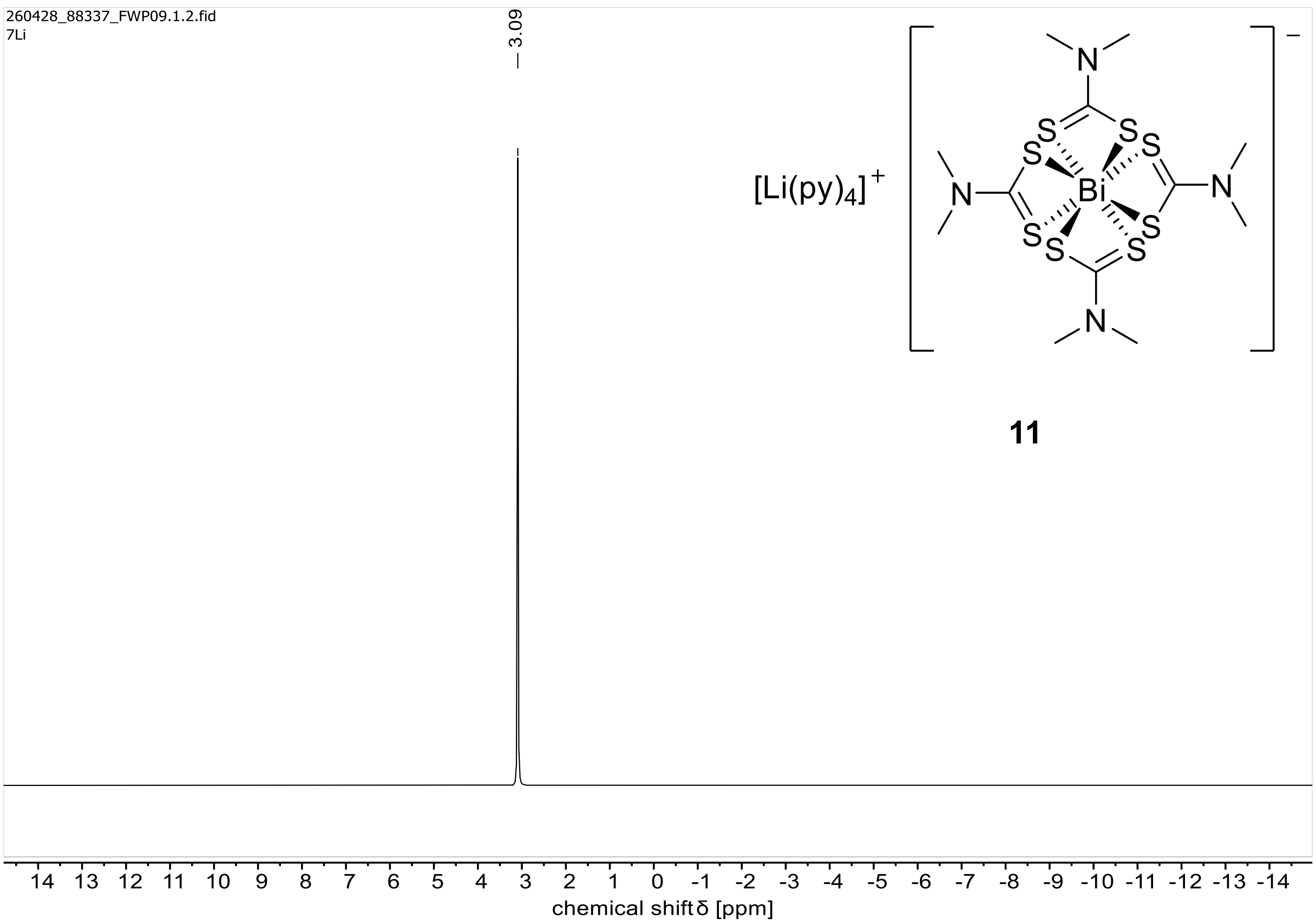

Figure S48: ^{7}Li NMR spectrum of **11** (194 MHz, 298 K, Py-d_5).

II. UV/Vis Spectra

In order to elucidate the absorption properties of the bismuthdithiocarbamates, UV/Vis spectra of two representative sets of compounds containing were recorded, varying either the halide atom (**4-Cl**, **4-Br**, **4-I**) or the number of dithiocarbamate ligands (**4-I** with one, **5-I** with two, **6** with three and **11** with four dithiocarbamate ligands) (Figure S49). UV/Vis spectra were recorded with a Lambda 25 UV/Vis spectrometer (PerkinElmer). The UV/Vis spectra of **4-X** (X = Cl, Br, I), **5-I**, **6** and **11** show a qualitatively similar behavior, despite exhibiting a variation of different colors in the solid state. However, when dissolved, all compounds except **4-Cl** and **4-Br** form a yellow solution in coordinating solvents like pyridine or THF, as well as in weakly-coordinating solvents like DCM. **4-Cl** and **4-Br** are colorless to off-white in the solid state and form colorless solutions at the given concentration. This agrees with the recorded UV/Vis spectra that show the weakest absorption in the visible range for **4-Cl** and **4-Br** out of all six compounds. In general, the absorption of visible light seems to correlate with the atomic number of the halogen atom, while all complexes show at least one strong absorption band in the range of λ = 233–255 nm and two weaker absorption bands in the range of λ = 300–480 nm (and an additional shoulder in the case of **5-I**). This suggests that the main absorption features are dictated by the presence of the bismuth dithiocarbamate structural motif, rather than the nature of the halide atom or the number of the dithiocarbamate ligands in the compound.

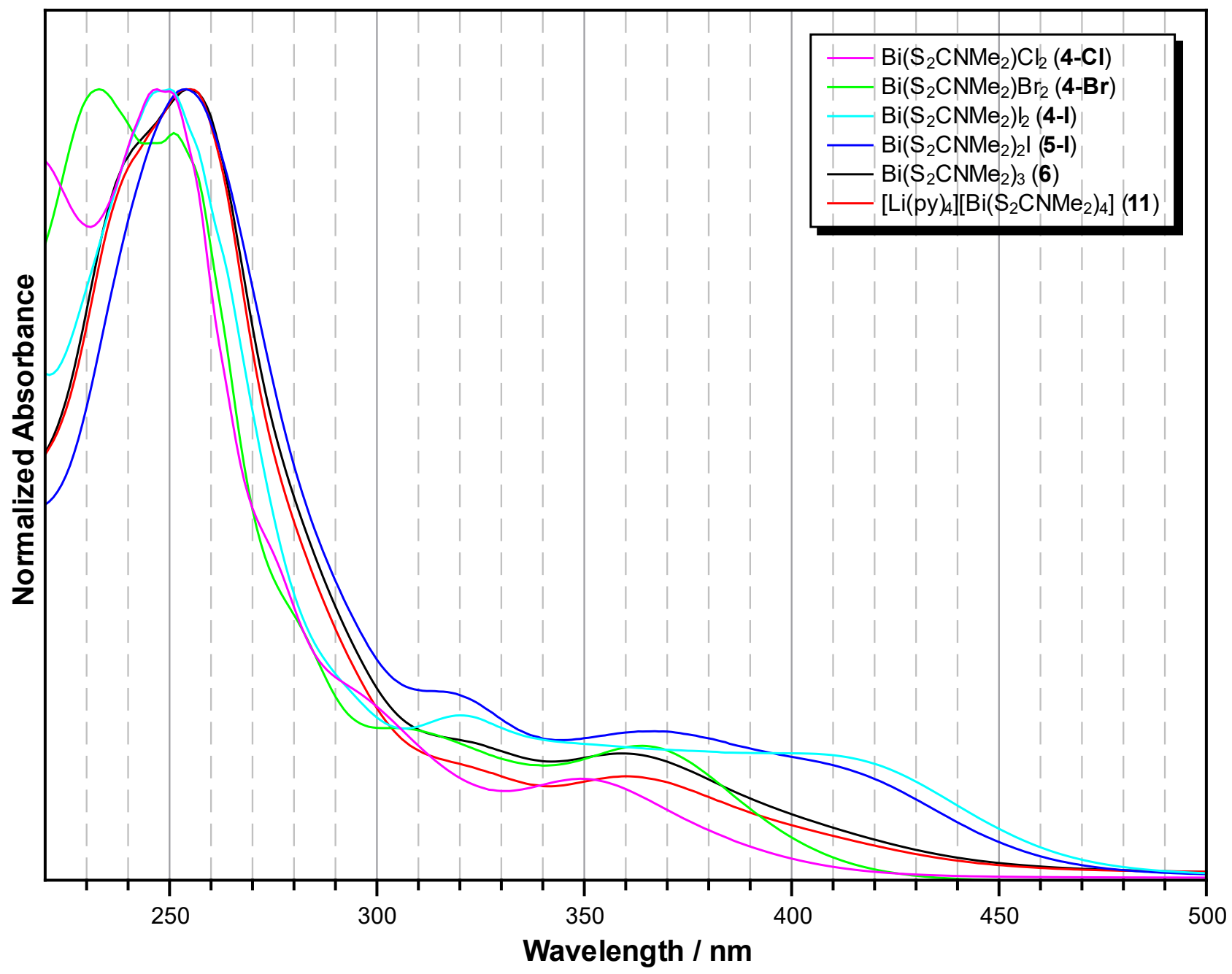

Figure S49: Representative UV/Vis spectra of the compounds **4-X** (X = Cl, Br, I), **5-I**, **6** and **11**. All spectra were recorded from a 0.05 mM solution of the respective compound in THF at ambient temperature (22 °C).

III. P-XRD, EDX and SEM Data

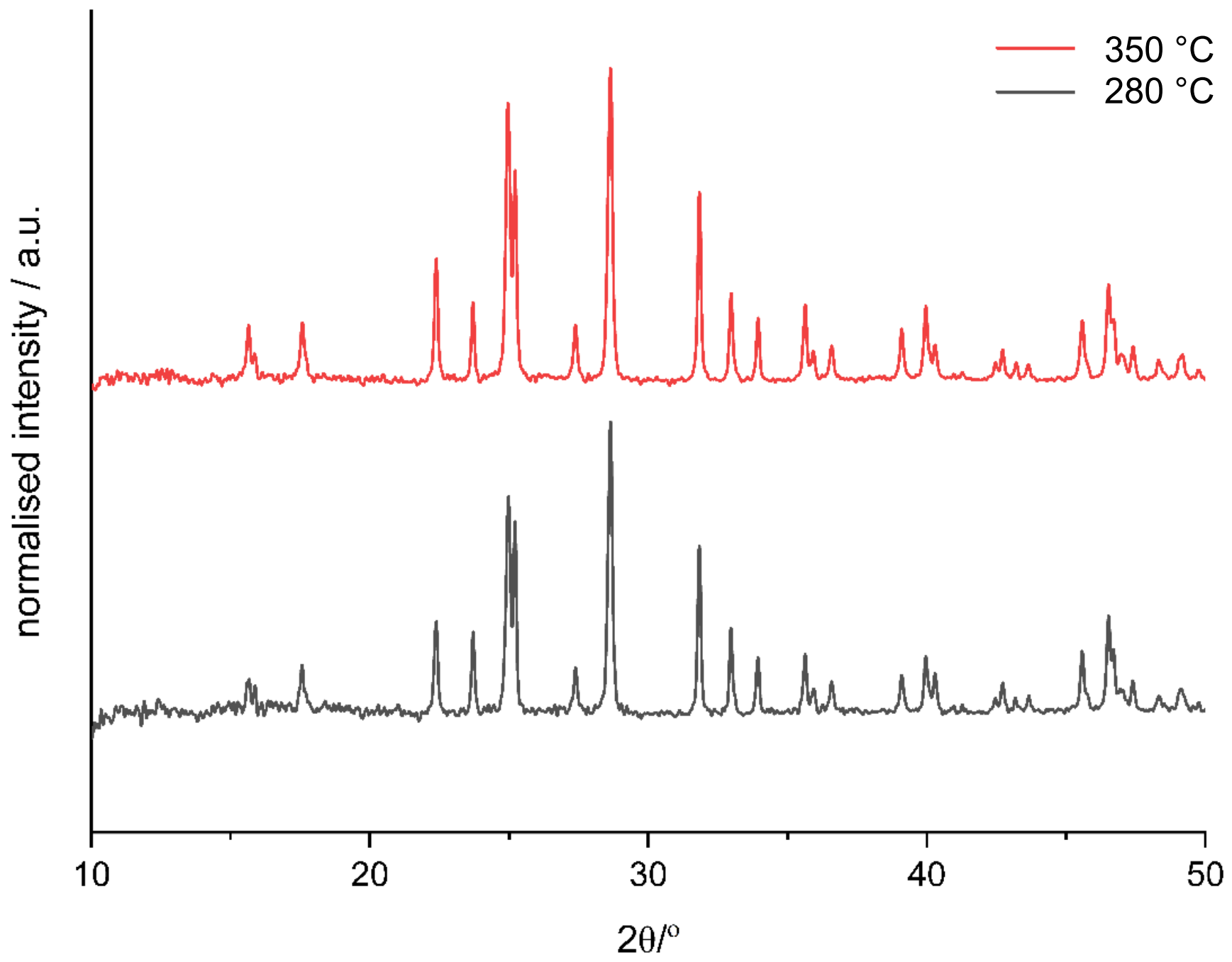

Figure S50: Powder X-Ray diffraction pattern of Bi_2S_3 prepared at 350 °C (red) and 280 °C (black) by thermal decomposition of $Bi(S_2CNMe_2)_3$ (**6**).

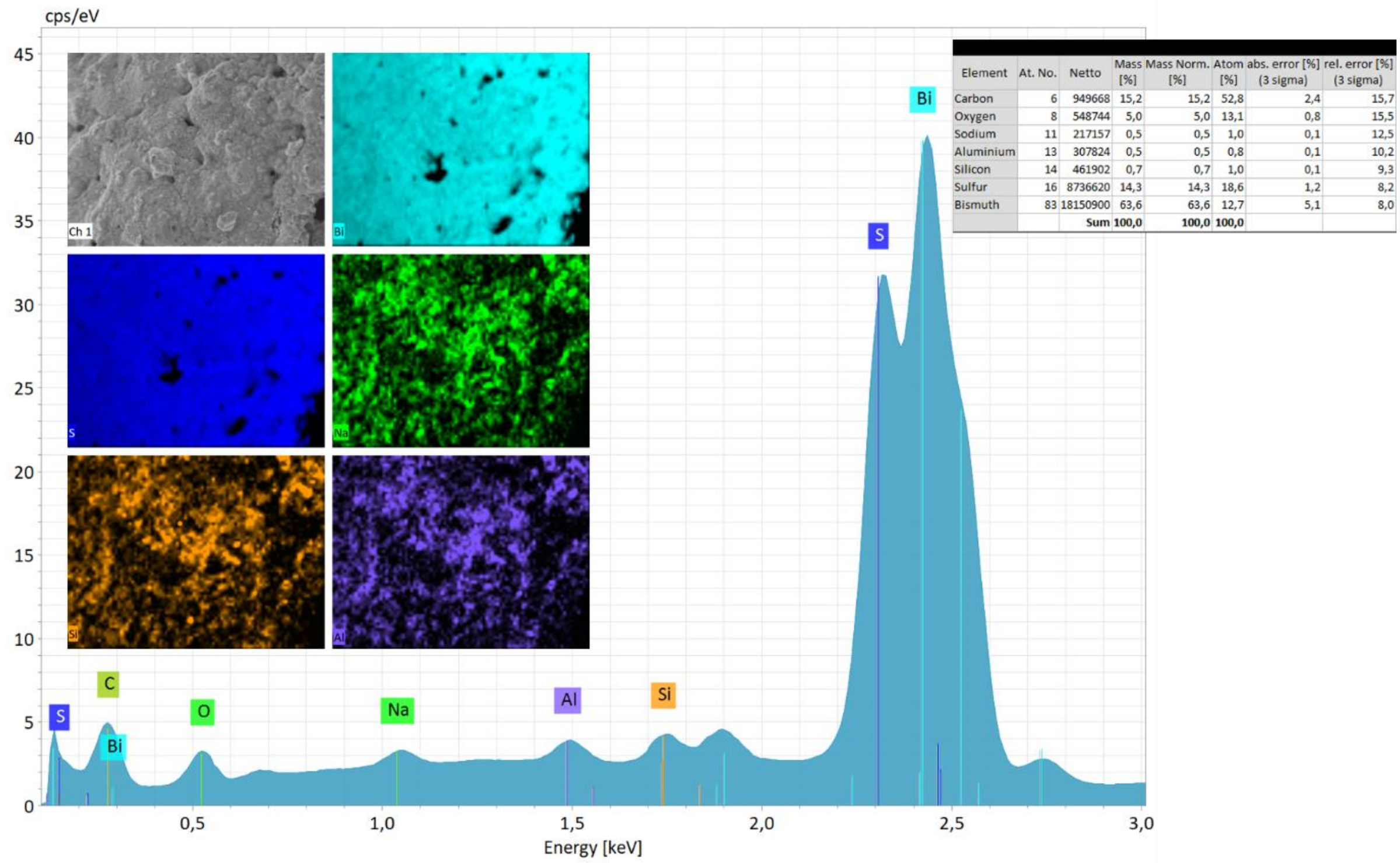

Element	At. No.	Netto	Mass [%]	Mass Norm. [%]	Atom [%]	abs. error [%] (3 sigma)	rel. error [%] (3 sigma)
Carbon	6	949668	15,2	15,2	52,8	2,4	15,7
Oxygen	8	548744	5,0	5,0	13,1	0,8	15,5
Sodium	11	217157	0,5	0,5	1,0	0,1	12,5
Aluminium	13	307824	0,5	0,5	0,8	0,1	10,2
Silicon	14	461902	0,7	0,7	1,0	0,1	9,3
Sulfur	16	8736620	14,3	14,3	18,6	1,2	8,2
Bismuth	83	18150900	63,6	63,6	12,7	5,1	8,0
		Sum	100,0	100,0	100,0		

Figure S51: EDX analyses with location-dependent element mapping of the at 350 °C prepared Bi_2S_3. The high carbon and oxygen content of the sample originates from the carbon containing sample holder as well as the sample preparation under ambient atmosphere.

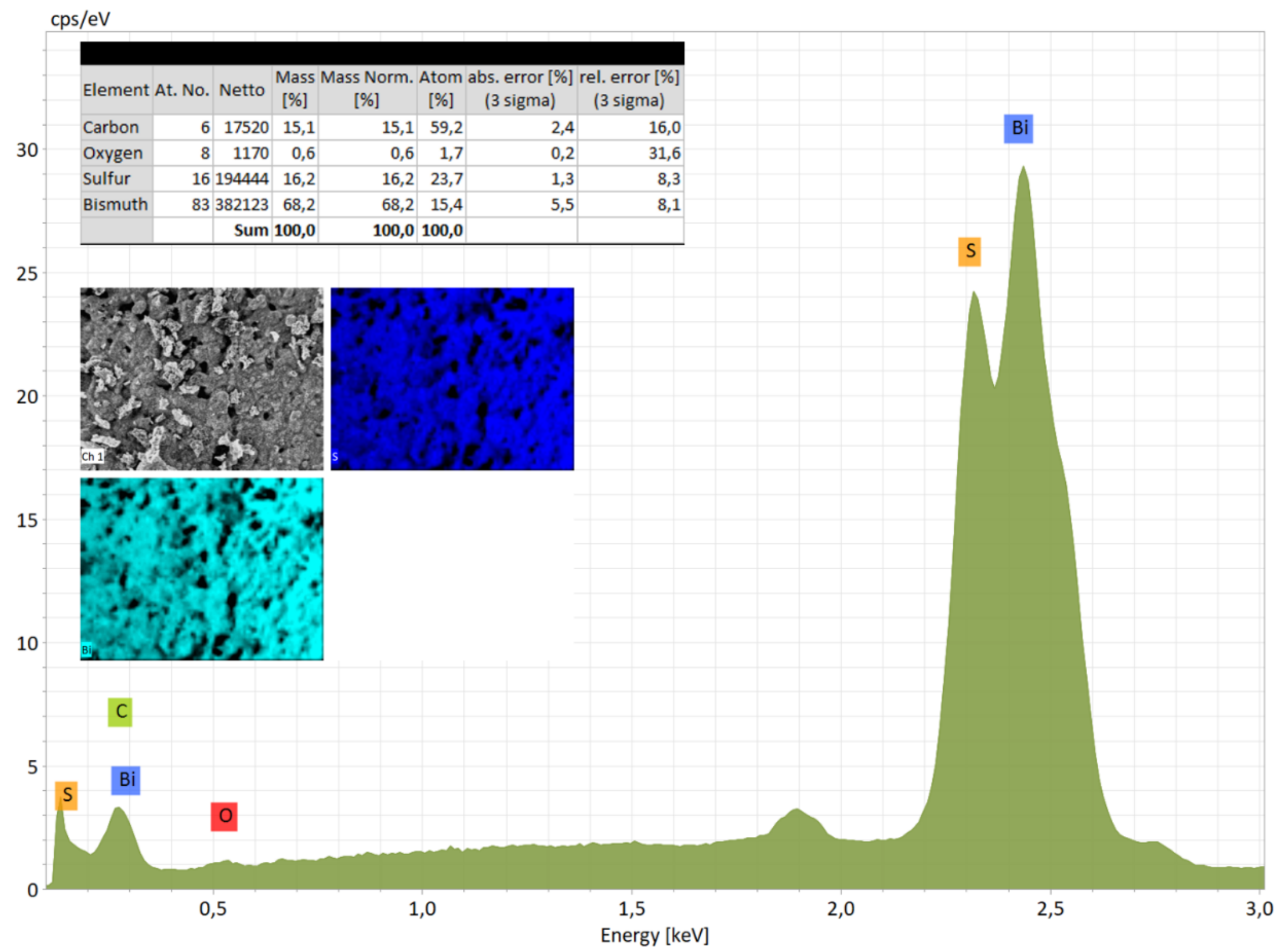

Figure S52: EDX analyses with location-dependent element mapping of the at 280 °C prepared Bi_2S_3. The high carbon content of the sample originates from the carbon containing sample holder.

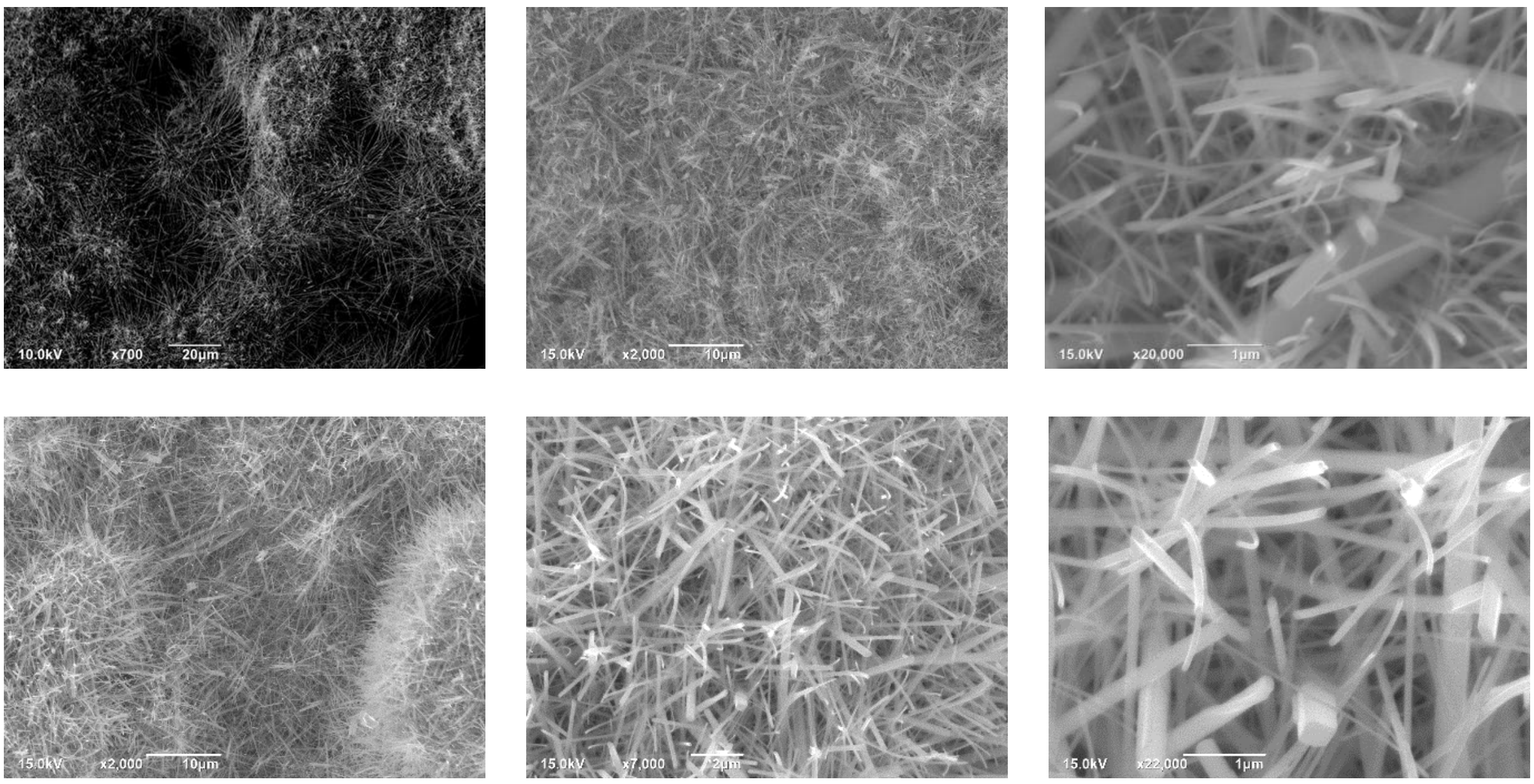

Figure S53: Representative SEM images of Bi_2S_3 prepared from $Bi(S_2CNMe_2)_3$ (**6**) at 280 °C (top) and 350 °C (bottom).

IV. X-Ray Diffraction Analysis

Single-crystals suitable for X-ray diffraction analysis were coated with polyisobutylene or perfluorinated polyether oil in a glovebox, transferred to a nylon loop, and then transferred to the goniometer of a Bruker D8 Quest or D8 Venture diffractometer equipped with Photon II or Photon III C14 detector using graphite-monochromated Mo-Kα radiation (λ = 0.71073 Å) and an Oxford Instrument Cooler Device for temperature control. Using Olex2, the structures were solved with the XT structure solution program using intrinsic phasing and refined with the XL refinement package using least-squares minimization.[1–3] Atomic Scattering Factors were taken from the *International Tables for X-Ray Crystallography.*[4] Empirical absorption corrections were introduced by using the MULTISCAN program.[3] All relevant non-hydrogen atoms were refined anisotropically. Hydrogen atoms were included in the structure factor calculations. All hydrogen atoms were assigned to idealized geometric positions. Molecular strautures in the solid state are visualized using the MERCURY and POV-Ray programs, with 50% probability displacement ellipsoids for non-hydrogen atoms. Depiction of hydrogen atoms is generally omitted for clarity. The CCDC deposition numbers 2566556–2566566 and 2583077 contain the supplementary crystallographic data for this publication. These data are provided free of charge by the joint Cambridge Crystallographic Data Centre and Fachinformationszentrum Karlsruhe (www.ccdc.cam.ac.uk/structures).

Table S1 – S12 summarize the relevant crystallographic data for compounds **2-Cl**, **2-Br**, **3-Cl**, **3-I**, **4-Cl**, **4-I**, **6**, **7**, **8**, **9**, **11** and [$CoCp_2$][S_2CNMe_2]. In addition, an ORTEP representation of each compound is provided with thermal ellipsoids drawn at 50% probability level. Hydrogen atoms and non-interacting solvent molecules have been omitted for clarity.

Table S1: Crystal and refinement data for $[Bi(NMe_2)Cl_2py]_2$ (**2-Cl**).

**Compound**	**$[Bi(NMe_2)Cl_2py]_2$ (2-Cl)**
CCDC Number	2566556
Empirical Formula	$C_{24}H_{32}Bi_2Cl_4N_6$
Formula Weight [g·mol^{-1}]	964.31
Temperature [K]	100
Crystal System	monoclinic
Space Group	*C*2/*c*
a [Å]	23.367(6)
b [Å]	8.738(2)
c [Å]	17.035(4)
α [°]	90
β [°]	117.563(12)
γ [°]	90
V [Å^3]	3083.3(14)
Z	4
ρ_{calc} [g cm^{-3}]	2.077
μ [mm^{-1}]	11.770
F(000)	1808.0
Crystal Size [mm^3]	0.294 × 0.160× 0.104
Used Radiation	Mo-Kα (λ = 0.71073)
$2\Theta_{min}$ / $2\Theta_{max}$	2.644 / 26.403
Index Ranges	$-29 \leq h \leq 29$, $-10 \leq k \leq 10$, $-21 \leq l \leq 21$
Reflections Collected	18909
Independent Reflections	3157
Data/Restraints/Parameters	3157 / 0 / 165
GooF on F^2	1.051
Final *R* Indices ($I \geq 2\sigma(I)$)	$R_1 = 0.0108$, $wR^2 = 0.0233$
Final *R* Indices (all data)	$R_1 = 0.0126$, $wR^2 = 0.0238$
Largest diff. Peak / Hole [e·Å^{-3}]	0.46 / –0.36

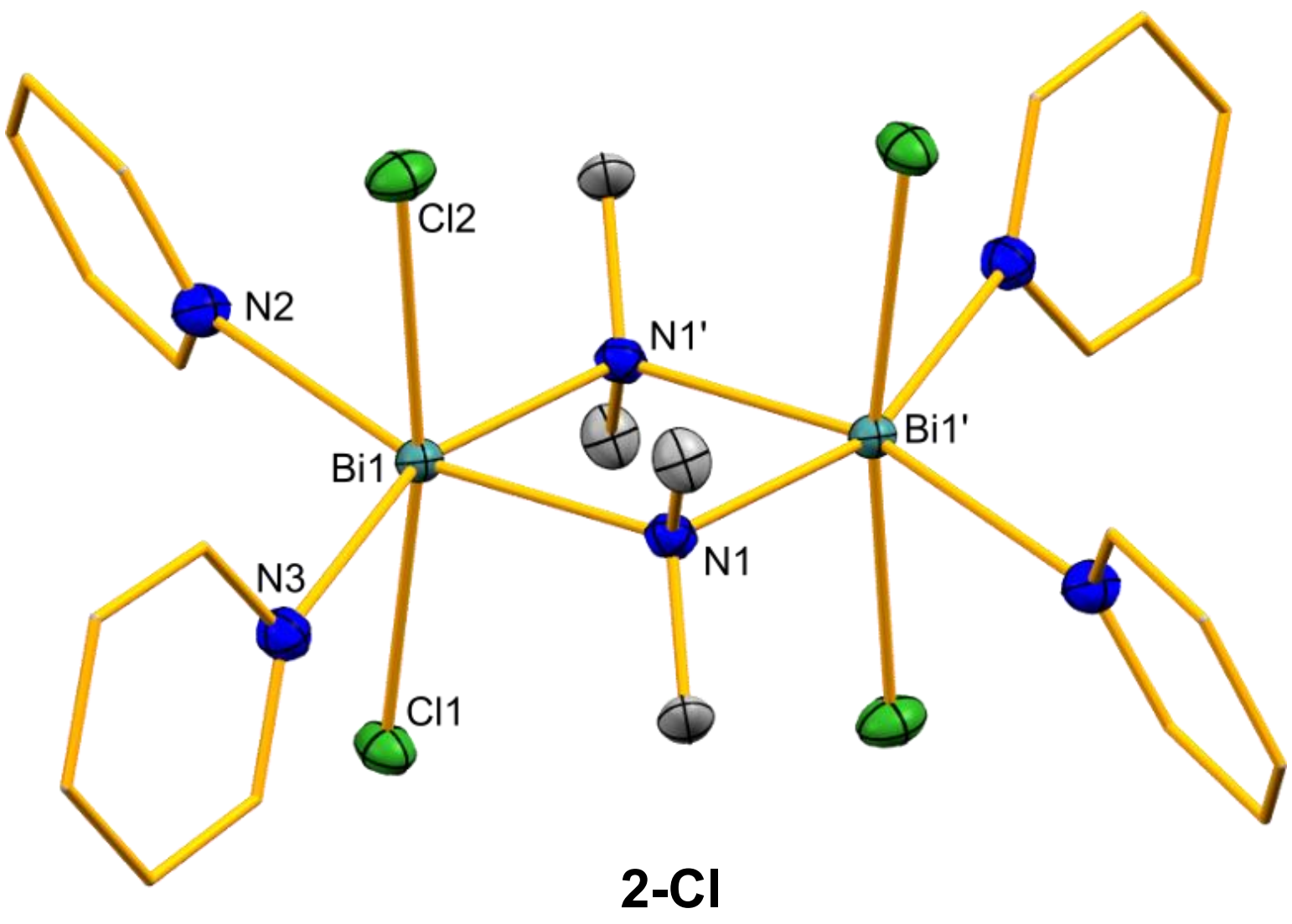

2-Cl

Table S2: Crystal and refinement data for $[Bi(NMe_2)Br_2py]_2$ (**2-Br**).

**Compound**	**$[Bi(NMe_2)Br_2py]_2$ (2-Br)**
CCDC Number	2566563
Empirical Formula	$C_{34}H_{41}Bi_2Br_4N_8$
Formula Weight [$g{\cdot}mol^{-1}$]	1299.35
Temperature [K]	300
Crystal System	monoclinic
Space Group	$P2_1/n$
a [Å]	18.4909(6)
b [Å]	11.9397(4)
c [Å]	18.6561(6)
α [°]	90
β [°]	98.0770(10)
γ [°]	90
V [$Å^3$]	4078.0(2)
Z	4
ρ_{calc} [$g\ cm^{-3}$]	2.116
μ [mm^{-1}]	12.572
F(000)	2428.0
Crystal Size [mm^3]	0.51 × 0.43× 0.23
Used Radiation	Mo-Kα (λ = 0.71073)
$2\Theta_{min}$ / $2\Theta_{max}$	4.062 / 50.054
Index Ranges	$-22 \leq h \leq 22$, $-14 \leq k \leq 14$, $-22 \leq l \leq 21$
Reflections Collected	110500
Independent Reflections	7193 [R_{int} = 0.0402, R_{sigma} = 0.0155]
Data/Restraints/Parameters	7193 / 0 / 437
GooF on F^2	1.066
Final R Indices ($I \geq 2\sigma(I)$)	R_1 = 0.0186, wR_2 = 0.0425
Final R Indices (all data)	R_1 = 0.0216, wR_2 = 0.0435
Largest diff. Peak / Hole [$e{\cdot}Å^{-3}$]	2.00 / −0.79

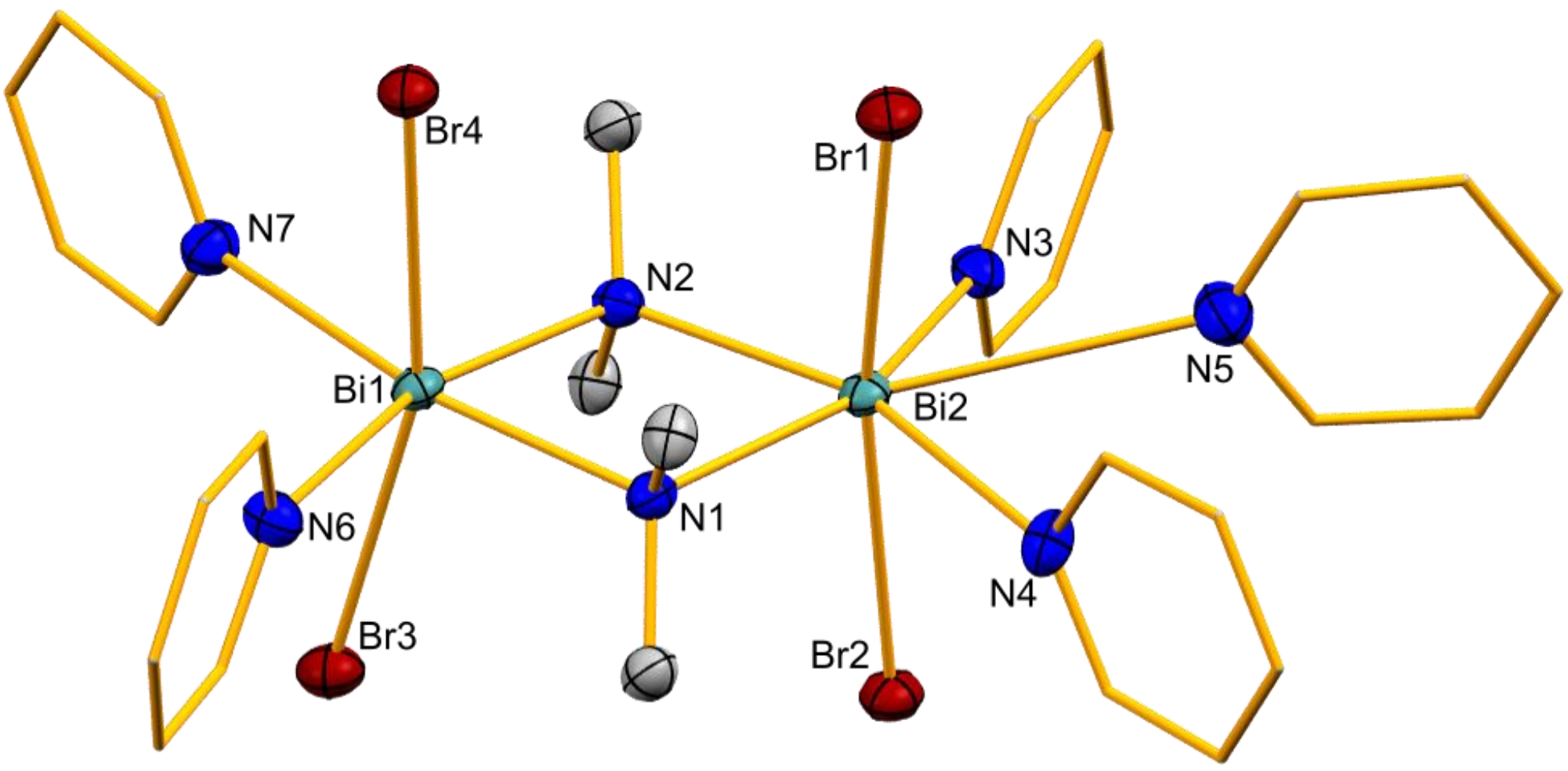

2-Br

Table S3: Crystal and refinement data for $[Bi(NMe_2)_2Cl]_\infty$ (**3-Cl**).

**Compound**	**$[Bi(NMe_2)_2Cl]_\infty$ (3-Cl)**
CCDC Number	2566561
Empirical Formula	$C_8H_{24}Bi_2Cl_2N_4$
Formula Weight [g·mol^{-1}]	665.17
Temperature [K]	100
Crystal System	monoclinic
Space Group	$P2_1/c$
a [Å]	13.5398(5)
b [Å]	8.5391(3)
c [Å]	15.7870(6)
α [°]	90
β [°]	112.5140(10)
γ [°]	90
V [Å^3]	1686.15(11)
Z	4
ρ_{calc} [g cm^{-3}]	2.620
μ [mm^{-1}]	21.147
F(000)	1200.0
Crystal Size [mm^3]	0.2 × 0.14 × 0.12
Used Radiation	Mo-Kα (λ = 0.71073)
$2\Theta_{min}$ / $2\Theta_{max}$	5.28 / 61.326
Index Ranges	$-19 \leq h \leq 19$, $-12 \leq k \leq 12$, $-22 \leq l \leq 21$
Reflections Collected	56825
Independent Reflections	5166 [R_{int} = 0.0463, R_{sigma} = 0.0204]
Data/Restraints/Parameters	5166 / 0 / 153
GooF on F^2	1.078
Final R Indices ($I \geq 2\sigma(I)$)	R_1 = 0.0178, wR_2 = 0.0364
Final R Indices (all data)	R_1 = 0.0219, wR_2 = 0.0370
Largest diff. Peak / Hole [e·Å^{-3}]	1.75 / −1.59

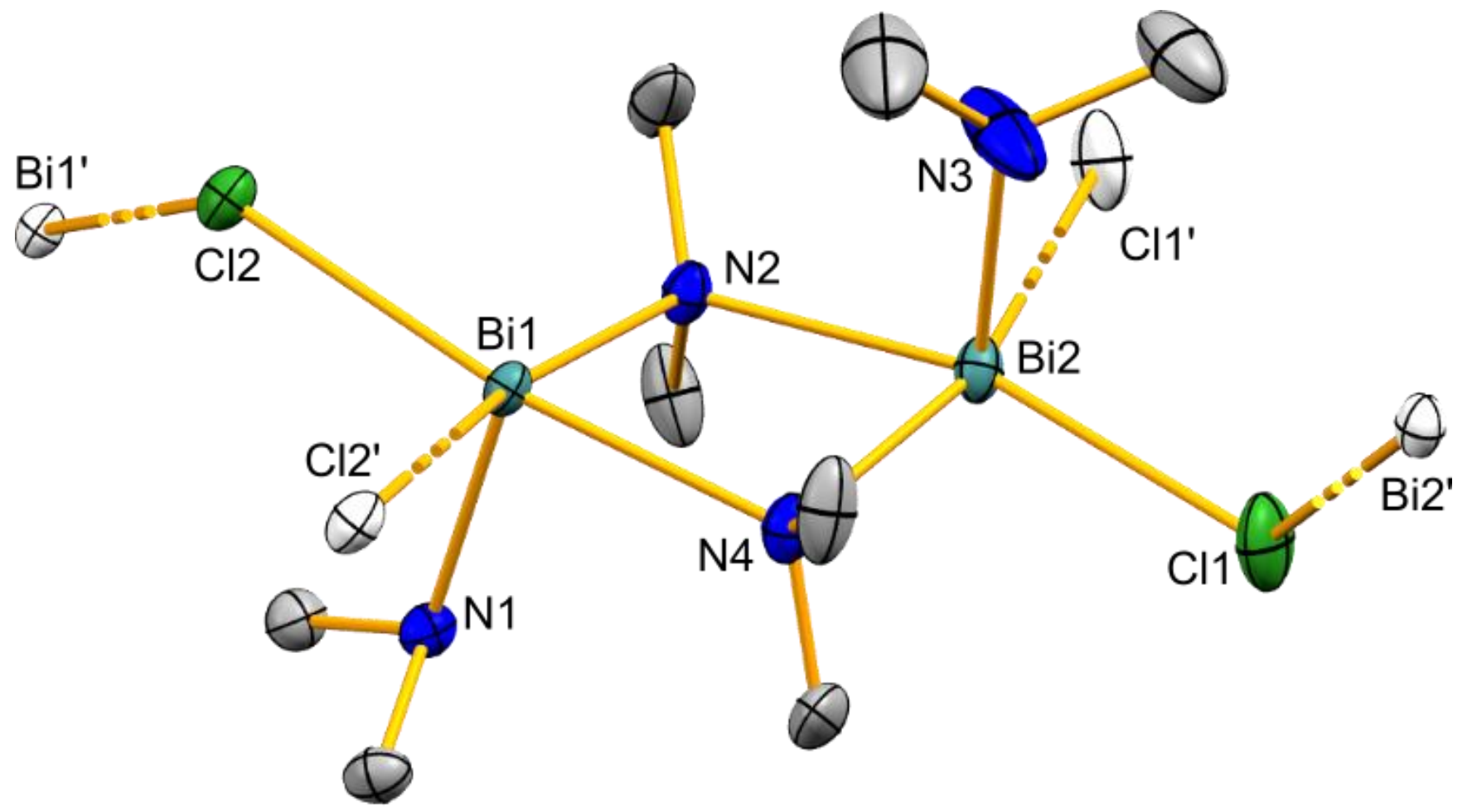

3-Cl

Table S4: Crystal and refinement data for $[Bi(NMe_2)_2I]_\infty$ (**3-I**).

**Compounda**	$\mathbf{[Bi(NMe_2)_2I]_\infty}$ **(3-I)**
CCDC Number	2566562
Empirical Formula	$C_{28}H_{44}Bi_2I_2N_8$
Formula Weight [g·mol^{-1}]	1164.47
Temperature [K]	100
Crystal System	orthorhombic
Space Group	$Pna2_1$
a [Å]	20.9902(8)
b [Å]	7.9465(3)
c [Å]	21.2301(7)
α [°]	90
β [°]	90
γ [°]	90
V [Å^3]	3541.2(2)
Z	4
ρ_{calc} [g cm^{-3}]	2.184
μ [mm^{-1}]	11.699
F(000)	2160.0
Crystal Size [mm^3]	0.076 × 0.043 × 0.035
Used Radiation	Mo-Kα (λ = 0.71073)
$2\Theta_{min}$ / $2\Theta_{max}$	3.88 / 56.594
Index Ranges	$-28 \leq h \leq 27$, $-10 \leq k \leq 10$, $-28 \leq l \leq 27$
Reflections Collected	87033
Independent Reflections	8772 [R_{int} = 0.0712, R_{sigma} = 0.0454]
Data/Restraints/Parameters	8772 / 1 / 363
GooF on F^2	1.022
Final R Indices ($I \geq 2\sigma(I)$)	R_1 = 0.0274, wR_2 = 0.0500
Final R Indices (all data)	R_1 = 0.0348, wR_2 = 0.0516
Largest diff. Peak / Hole [e·Å^{-3}]	1.91 / −0.78

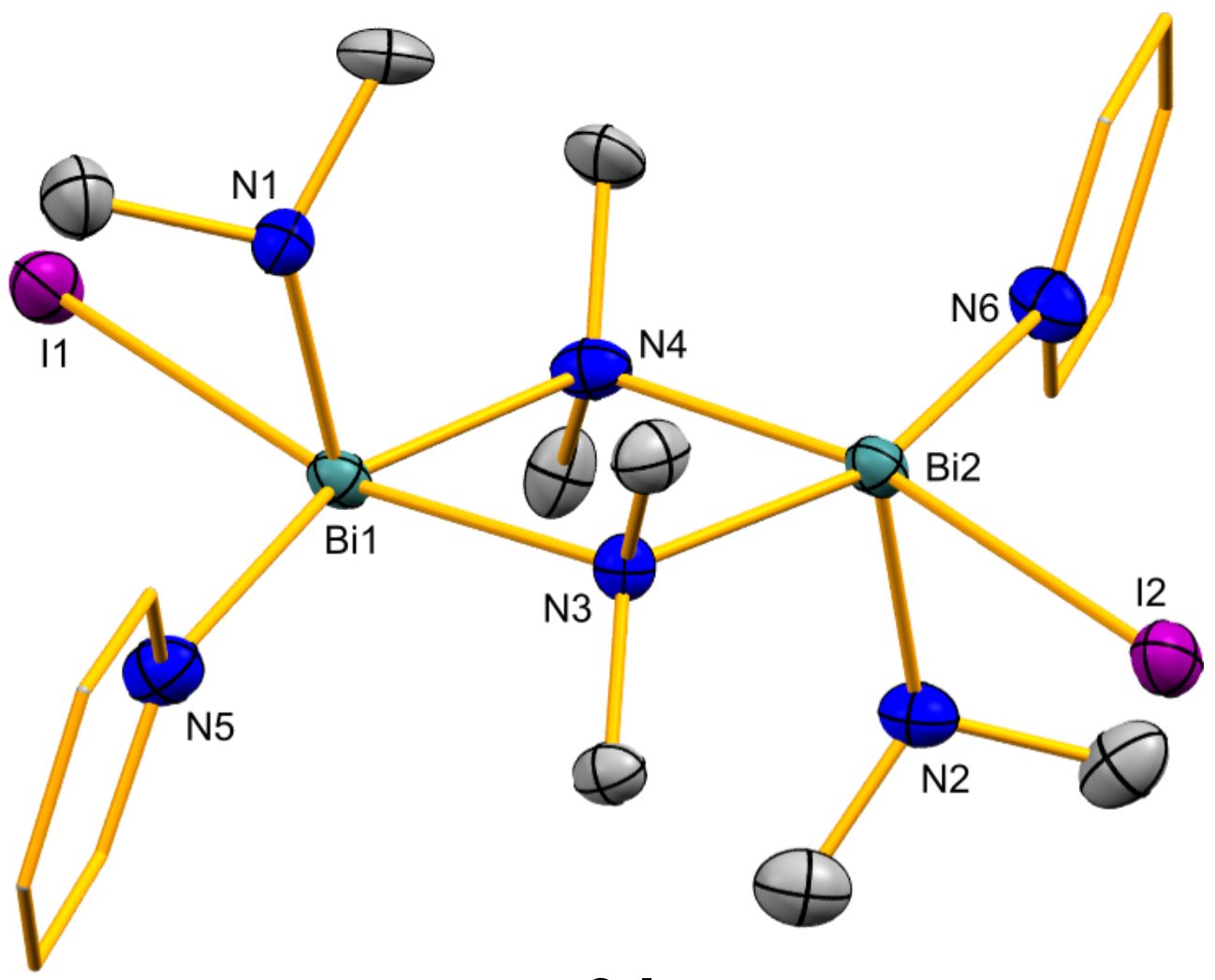

3-I

Table S5: Crystal and refinement data for $Bi(CS_2NMe_2)Cl_2py_3$ (**4-Cl**).

**Compound**	**$Bi(CS_2NMe_2)Cl_2py_3$ (4-Cl)**
CCDC Number	2566558
Empirical Formula	$C_{18}H_{21}BiCl_2N_4S_2$
Formula Weight [g·mol^{-1}]	637.412
Temperature [K]	100
Crystal System	orthorhombic
Space Group	*Pbca*
a [Å]	9.463(4)
b [Å]	15.827(4)
c [Å]	31.903(6)
α [°]	90
β [°]	90
γ [°]	90
V [Å^3]	4778(2)
Z	8
ρ_{calc} [g cm^{-3}]	1.772
μ [mm^{-1}]	7.788
F(000)	2426.9
Crystal Size [mm^3]	0.168 × 0.102 × 0.053
Used Radiation	Mo-Kα (λ = 0.71073)
$2\Theta_{min}$ / $2\Theta_{max}$	5 to 51.3
Index Ranges	$-11 \leq h \leq 11$, $-19 \leq k \leq 19$, $-38 \leq l \leq 38$
Reflections Collected	65987
Independent Reflections	4524 [R_{int} = 0.1190, R_{sigma} = 0.0444]
Data/Restraints/Parameters	4524 / 0 / 246
GooF on F^2	1.050
Final R Indices ($I \geq 2\sigma(I)$)	R_1 = 0.0328, wR_2 = 0.0527
Final R Indices (all data)	R_1 = 0.0577, wR_2 = 0.0623
Largest diff. Peak / Hole [e·Å^{-3}]	1.25 / −1.20

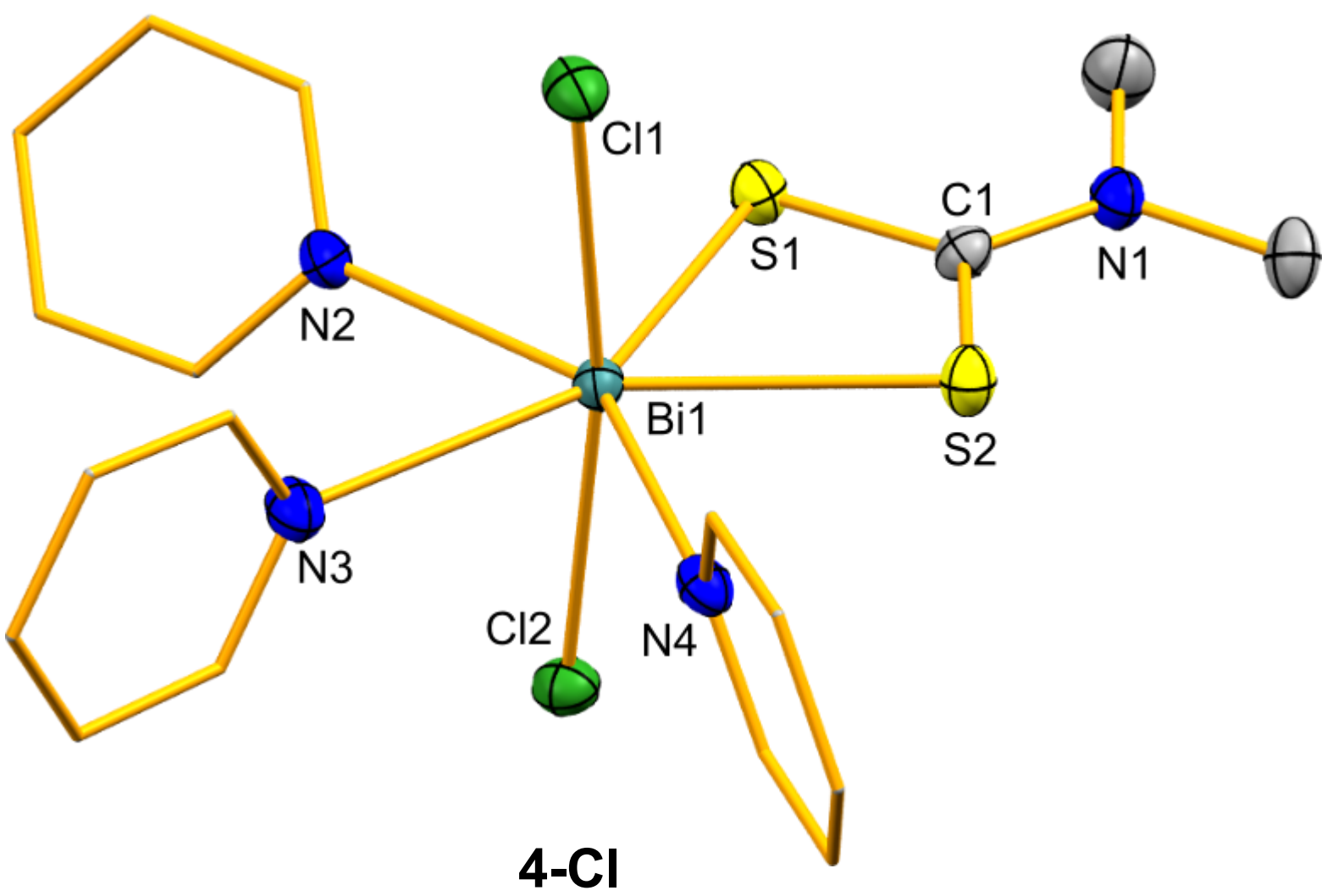

4-Cl

Table S6: Crystal and refinement data for $Bi(CS_2NMe_2)I_2py_3$ (**4-I**).

**Compound**	**$Bi(CS_2NMe_2)I_2py_3$ (4-I)**
CCDC Number	2566560
Empirical Formula	$C_{18}H_{21}BiI_2N_4S_2$
Formula Weight [g·mol^{-1}]	820.29
Temperature [K]	100
Crystal System	monoclinic
Space Group	$P2_1$
a [Å]	10.1173(4)
b [Å]	12.8762(5)
c [Å]	10.4472(4)
α [°]	90
β [°]	116.9690(10)
γ [°]	90
V [Å^3]	1212.98(8)
Z	2
ρ_{calc} [g cm^{-3}]	2.246
μ [mm^{-1}]	9.996
F(000)	756.0
Crystal Size [mm^3]	0.099 × 0.073 × 0.072
Used Radiation	Mo-Kα (λ = 0.71073)
$2\Theta_{min}$ / $2\Theta_{max}$	4.374 / 56.602
Index Ranges	$-13 \le h \le 13$, $-17 \le k \le 17$, $-13 \le l \le 13$
Reflections Collected	59400
Independent Reflections	5803 [R_{int} = 0.0342, R_{sigma} = 0.0276]
Data/Restraints/Parameters	5803 / 1 / 246
GooF on F^2	0.951
Final R Indices ($I \ge 2\sigma(I)$)	R_1 = 0.0133, wR_2 = 0.0273
Final R Indices (all data)	R_1 = 0.0139, wR_2 = 0.0276
Largest diff. Peak / Hole [e·Å^{-3}]	0.48 / −0.36

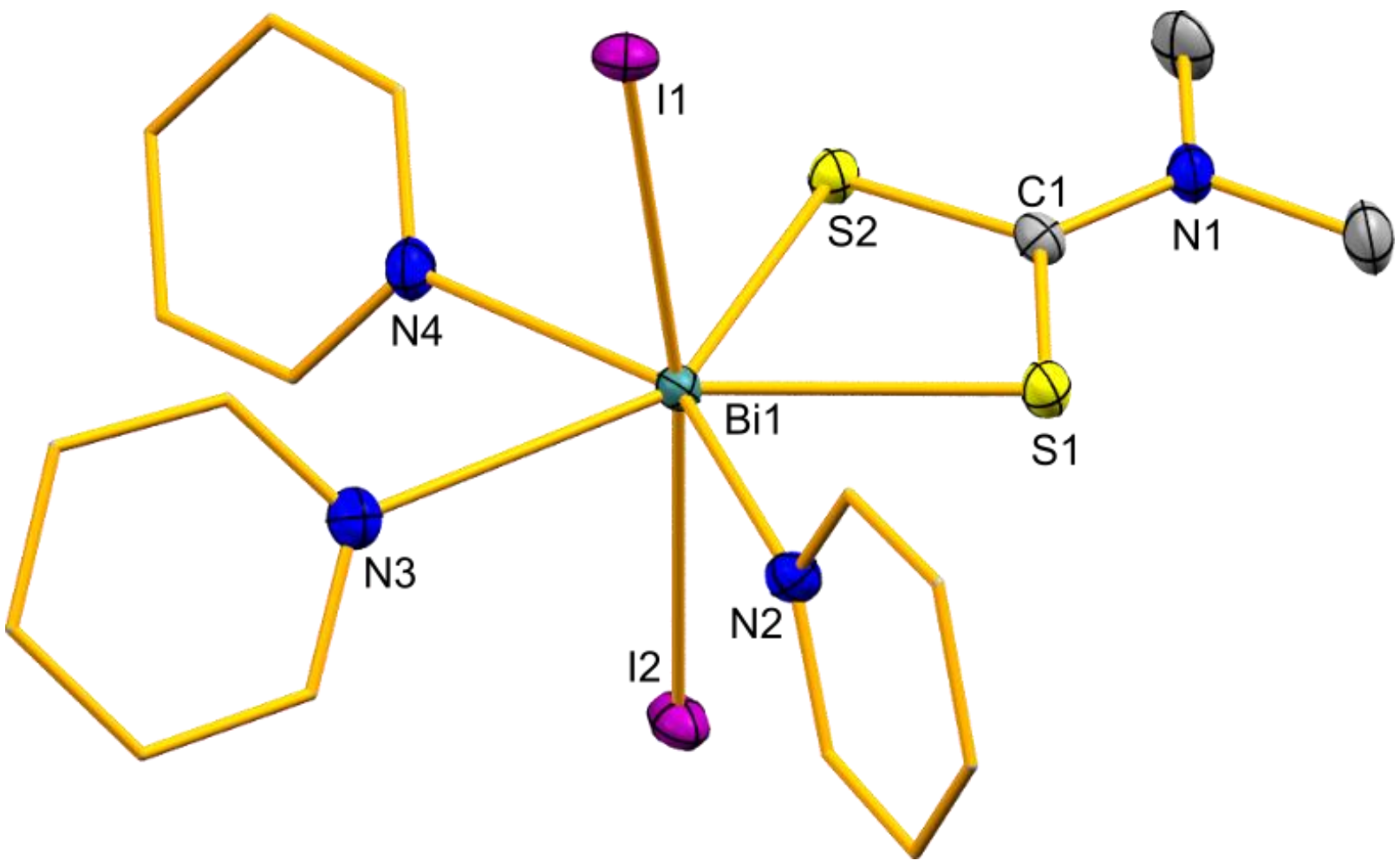

4-I

Table S7: Crystal and refinement data for $[Bi(CS_2NMe_2)_3]_2$ (**6**).

**Compound**	**$[Bi(CS_2NMe_2)_3]_2$ (6)**
CCDC Number	2566557
Empirical Formula	$C_9H_{18}BiN_3S_6$
Formula Weight [g·mol^{-1}]	569.60
Temperature [K]	100
Crystal System	triclinic
Space Group	$P\bar{1}$
a [Å]	9.651(7)
b [Å]	9.827(6)
c [Å]	11.369(12)
α [°]	87.87(6)
β [°]	67.31(6)
γ [°]	65.27(4)
V [Å^3]	893.6(13)
Z	2
ρ_{calc} [g cm^{-3}]	2.117
μ [mm^{-1}]	10.557
F(000)	544.0
Crystal Size [mm^3]	0.116 × 0.103 × 0.065
Used Radiation	Mo-Kα (λ = 0.71073)
$2\Theta_{min}$ / $2\Theta_{max}$	3.926 / 56.766
Index Ranges	$-12 \leq h \leq 12$, $-13 \leq k \leq 13$, $-15 \leq l \leq 15$
Reflections Collected	18071
Independent Reflections	4413 [R_{int} = 0.0429, R_{sigma} = 0.0393]
Data/Restraints/Parameters	4413 / 0 / 178
GooF on F^2	1.093
Final *R* Indices ($I \geq 2\sigma(I)$)	R_1 = 0.0287, wR_2 = 0.0572
Final *R* Indices (all data)	R_1 = 0.0335, wR_2 = 0.0589
Largest diff. Peak / Hole [e·Å^{-3}]	2.33 / −1.98

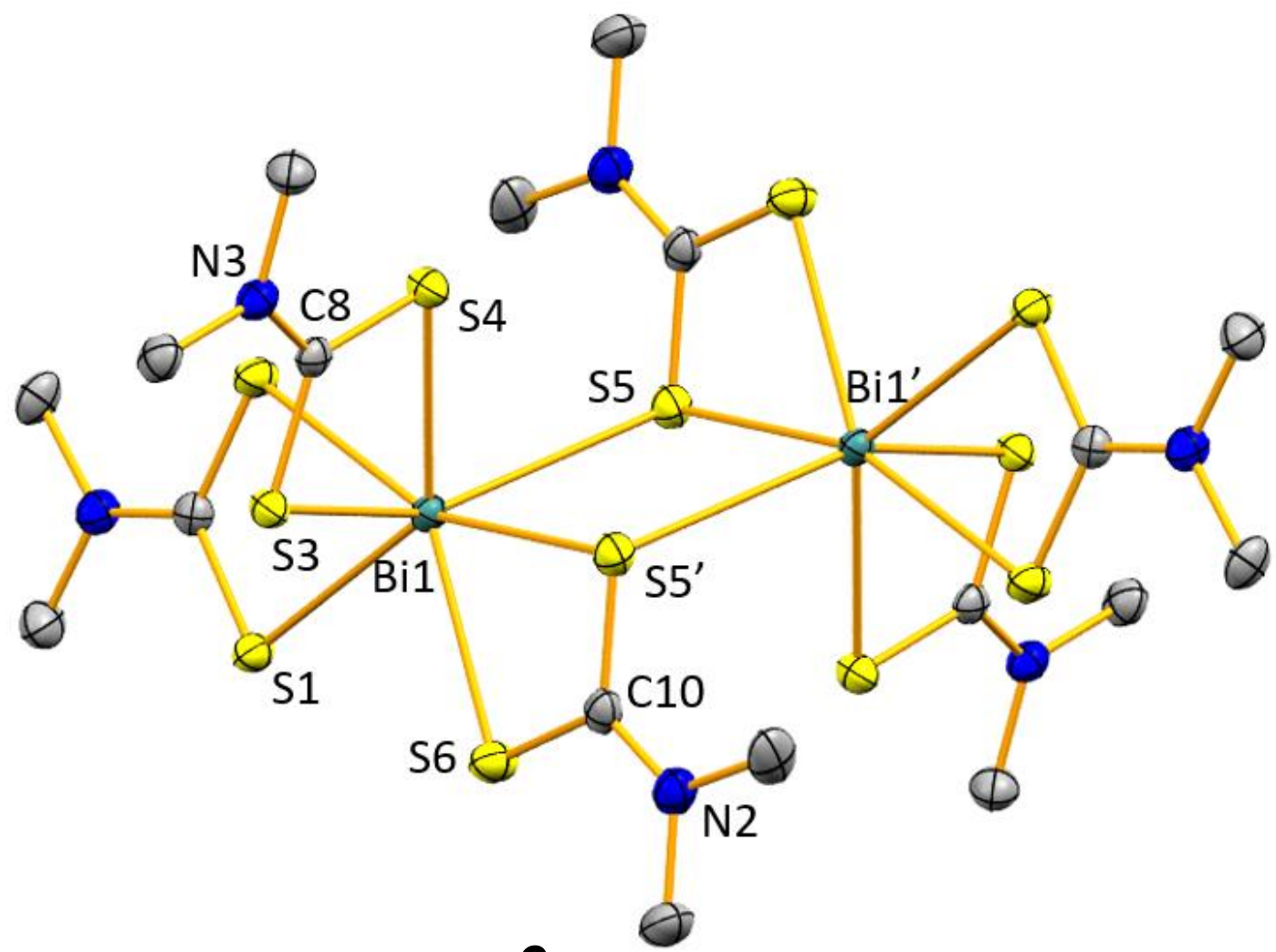

6

Table S8: Crystal and refinement data for [^{Me2}CAAC–Bi(CS_2NMe_2)Cl_2] (**7**).

**Compound**	**[^{Me2}CAAC–Bi(CS_2NMe_2)Cl_2] (7)**
CCDC Number	2566564
Empirical Formula	$C_{27}H_{45}BiCl_2N_2OS_2$
Formula Weight [g·mol^{-1}]	757.65
Temperature [K]	100
Crystal System	monoclinic
Space Group	$P2_1/c$
a [Å]	11.2504(3)
b [Å]	15.7619(4)
c [Å]	18.4123(5)
α [°]	90
β [°]	102.7860(10)
γ [°]	90
V [Å^3]	3184.05(15)
Z	4
ρ_{calc} [g cm^{-3}]	1.581
μ [mm^{-1}]	5.858
F(000)	1512.0
Crystal Size [mm^3]	0.22 × 0.12 × 0.07
Used Radiation	Mo-Kα (λ = 0.71073)
$2\Theta_{min}$ / $2\Theta_{max}$	4.524 / 61.136
Index Ranges	$-16 \le h \le 16$, $-22 \le k \le 22$, $-26 \le l \le 26$
Reflections Collected	117735
Independent Reflections	9729 [R_{int} = 0.0397, R_{sigma} = 0.0211]
Data/Restraints/Parameters	9729 / 607 / 372
GooF on F^2	1.070
Final R Indices ($I \ge 2\sigma(I)$)	R_1 = 0.0381, wR_2 = 0.0947
Final R Indices (all data)	R_1 = 0.0506, wR_2 = 0.1009
Largest diff. Peak / Hole [e·Å^{-3}]	5.98 / −1.04

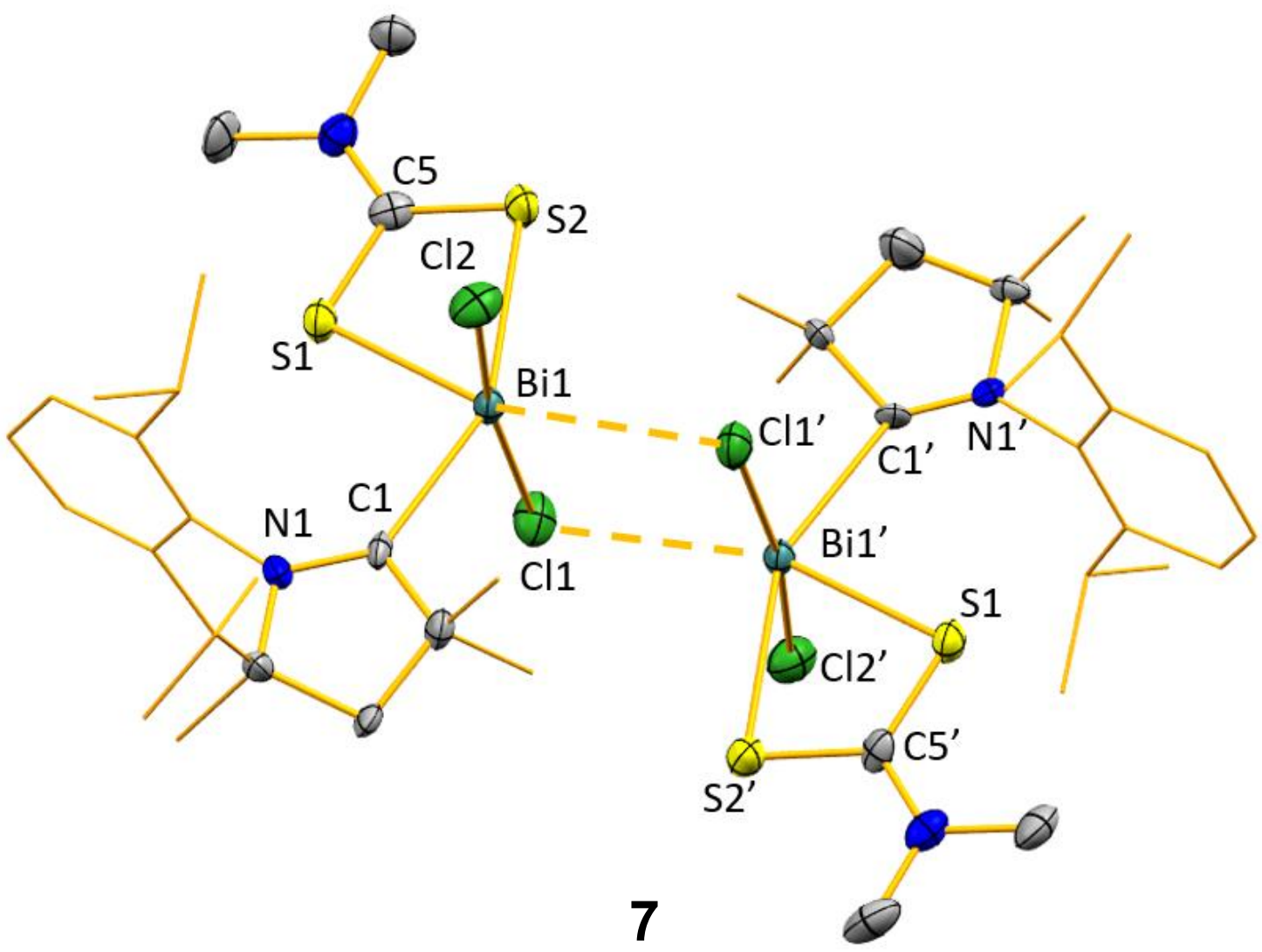

7

Table S9: Crystal and refinement data for [^{Me2}CAAC–Bi(CS_2NMe_2)$_2$Br] (**8**).

**Compound**	**[^{Me2}CAAC–Bi(CS_2NMe_2)$_2$Br] (8)**
CCDC Number	2566559
Empirical Formula	$C_{72}H_{126}Bi_2Br_2N_6O_5S_8$
Formula Weight [g·mol^{-1}]	1990.04
Temperature [K]	100
Crystal System	monoclinic
Space Group	*C*2/*c*
a [Å]	25.1526(12)
b [Å]	18.8712(8)
c [Å]	18.3270(9)
α [°]	90
β [°]	103.160(2)
γ [°]	90
V [Å^3]	8470.6(7)
Z	4
ρ_{calc} [g cm^{-3}]	1.560
μ [mm^{-1}]	5.338
F(000)	4016.0
Crystal Size [mm^3]	0.182 × 0.120 × 0.093
Used Radiation	Mo-Kα (λ = 0.71073)
$2\Theta_{min}$ / $2\Theta_{max}$	3.790 / 59.610
Index Ranges	$-32 \leq h \leq 35$, $-26 \leq k \leq 26$, $-25 \leq l \leq 25$
Reflections Collected	121212
Independent Reflections	12102 [R_{int} = 0.0827, R_{sigma} = 0.0469]
Data/Restraints/Parameters	12102 / 0 / 441
GooF on F^2	1.056
Final *R* Indices ($I \geq 2\sigma(I)$)	R_1 = 0.0367, wR_2 = 0.0735
Final *R* Indices (all data)	R_1 = 0.0564, wR_2 = 0.0794
Largest diff. Peak / Hole [e·Å^{-3}]	2.06 / −1.84

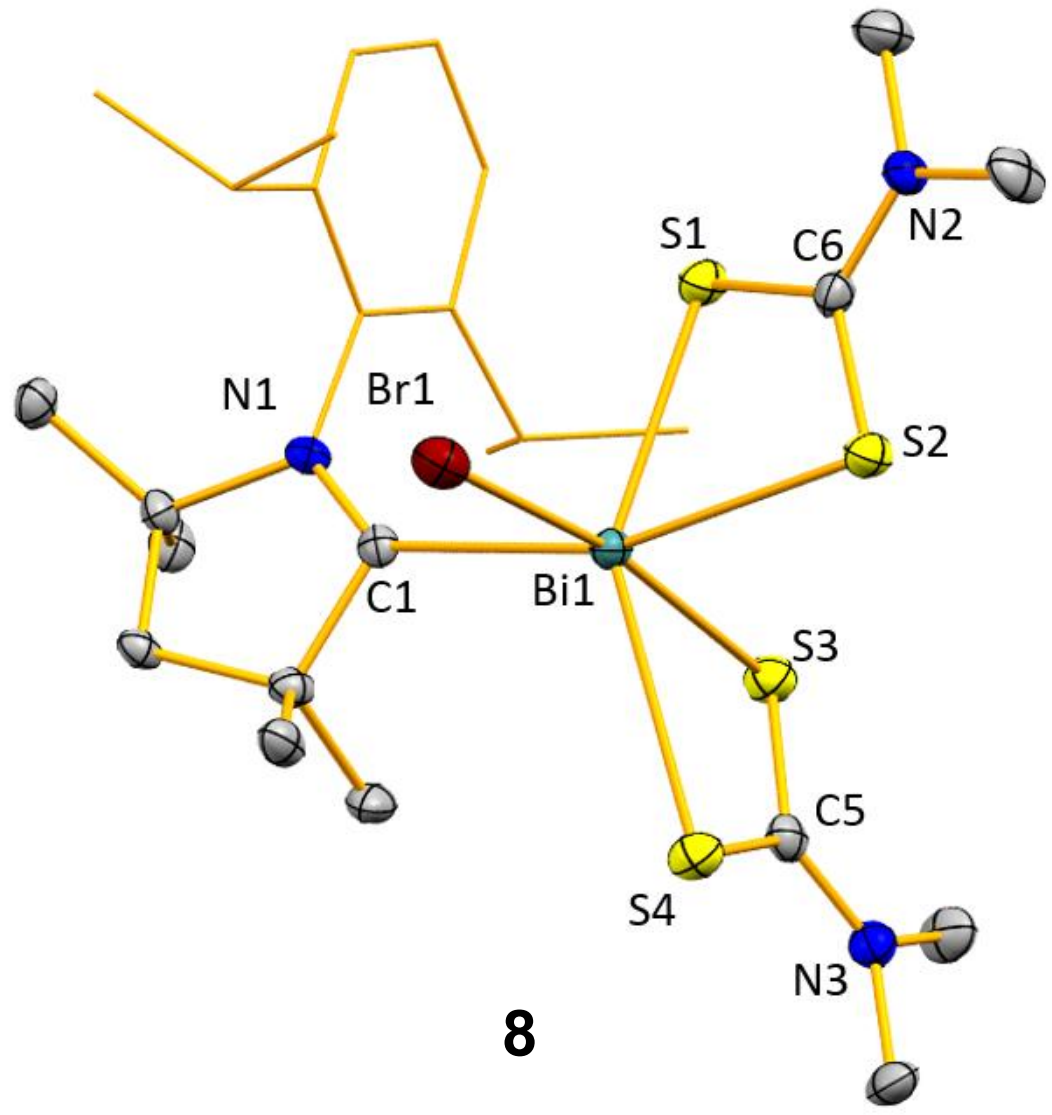

8

Table S10: Crystal and refinement data for [^{Me2}CAAC–SiMe$_3$][Bi(CS$_2$NMe$_2$)$_4$] (**9**).

**Compound**	**[^{Me2}CAAC–SiMe$_3$][Bi(CS$_2$NMe$_2$)$_4$] (9)**
CCDC Number	2566565
Empirical Formula	$C_{39}H_{72}BiN_5OS_8Si$
Formula Weight [g·mol^{-1}]	1120.56
Temperature [K]	293
Crystal System	monoclinic
Space Group	$P2_1$
a [Å]	9.6838(4)
b [Å]	18.3525(6)
c [Å]	14.8798(6)
α [°]	90
β [°]	105.0110(10)
γ [°]	90
V [Å^3]	2554.23(17)
Z	4
ρ_{calc} [g cm^{-3}]	1.457
μ [mm^{-1}]	3.836
F(000)	1148.0
Crystal Size [mm^3]	0.260 × 0.162 × 0.092
Used Radiation	Mo-Kα (λ = 0.71073)
$2\Theta_{min}$ / $2\Theta_{max}$	4.354 / 61.008
Index Ranges	$-13 \leq h \leq 13$, $-26 \leq k \leq 21$, $-21 \leq l \leq 21$
Reflections Collected	45790
Independent Reflections	12681 [R_{int} = 0.0304, R_{sigma} = 0.0445]
Data/Restraints/Parameters	12681 / 3 / 526
GooF on F^2	0.965
Final R Indices ($I \geq 2\sigma(I)$)	R_1 = 0.0208, wR_2 = 0.0415
Final R Indices (all data)	R_1 = 0.0230, wR_2 = 0.0420
Largest diff. Peak / Hole [e·Å^{-3}]	0.80 / −0.68

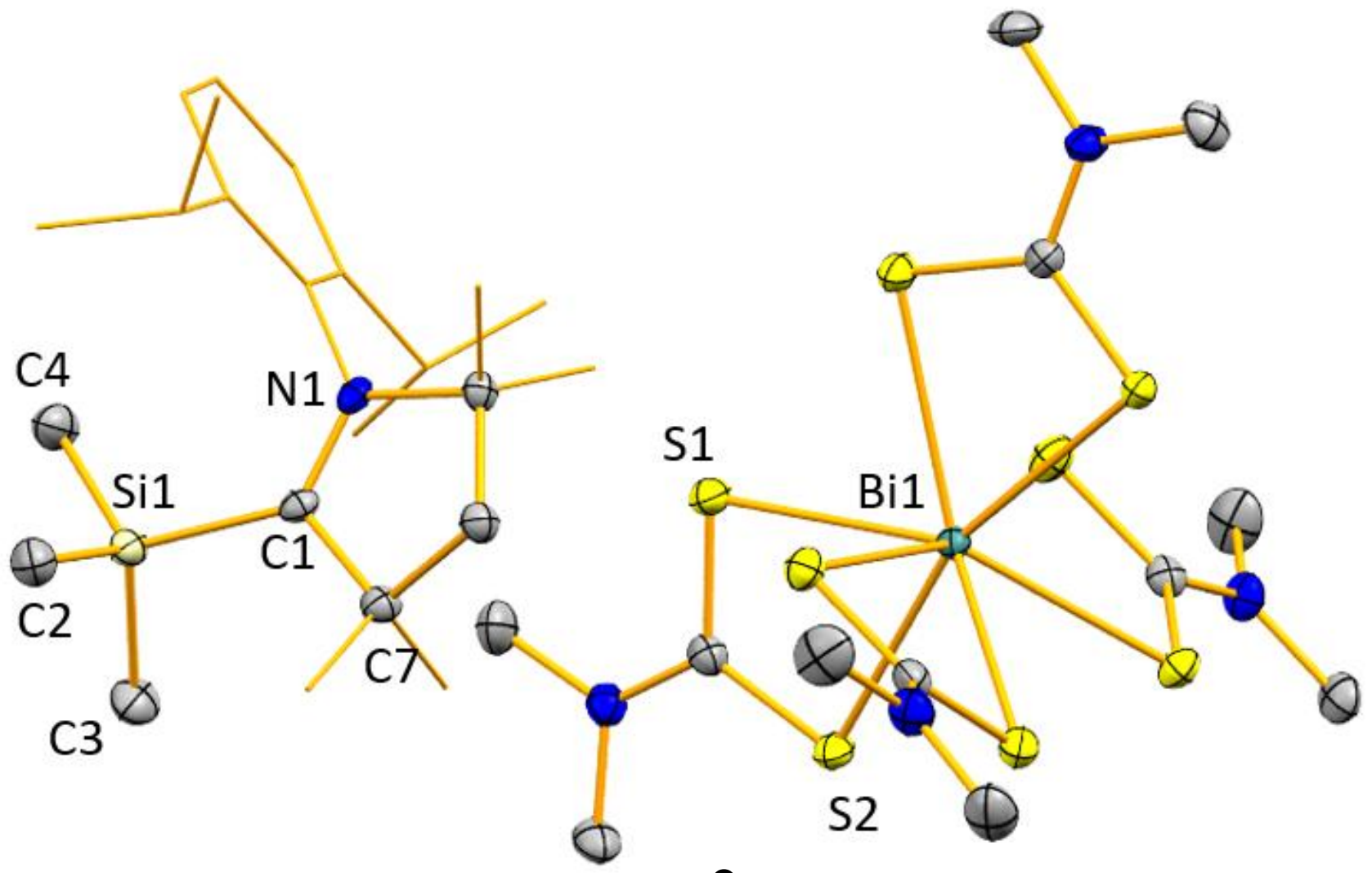

9

Table S11: Crystal and refinement data for $[Li(py)_4][Bi(S_2CNMe_2)_4]$ (**11**).

**Compound**	**$[Li(py)_4][Bi(S_2CNMe_2)_4]$ (11)**
CCDC Number	2566566
Empirical Formula	$C_{32}H_{44}BiLiN_8S_8$
Formula Weight [$g{\cdot}mol^{-1}$]	1013.15
Temperature [K]	100
Crystal System	monoclinic
Space Group	*C*2/*c*
a [Å]	18.3455(6)
b [Å]	12.9177(4)
c [Å]	17.7880(5)
α [°]	90
β [°]	91.2300(10)
γ [°]	90
V [$Å^3$]	4214.5(2)
Z	4
ρ_{calc} [$g\ cm^{-3}$]	1.597
μ [mm^{-1}]	4.614
F(000)	2024.0
Crystal Size [mm^3]	0.191 × 0.107 × 0.082
Used Radiation	Mo-Kα (λ = 0.71073)
$2\Theta_{min}$ / $2\Theta_{max}$	3.856 / 49.998
Index Ranges	$-21 \leq h \leq 21$, $-15 \leq k \leq 15$, $-21 \leq l \leq 21$
Reflections Collected	42739
Independent Reflections	3715 [R_{int} = 0.0263, R_{sigma} = 0.0118]
Data/Restraints/Parameters	3715 / 0 / 231
GooF on F^2	1.158
Final *R* Indices ($I \geq 2\sigma(I)$)	R_1 = 0.0189, wR_2 = 0.0460
Final *R* Indices (all data)	R_1 = 0.0192, wR_2 = 0.0461
Largest diff. Peak / Hole [$e{\cdot}Å^{-3}$]	2.89 / −0.71

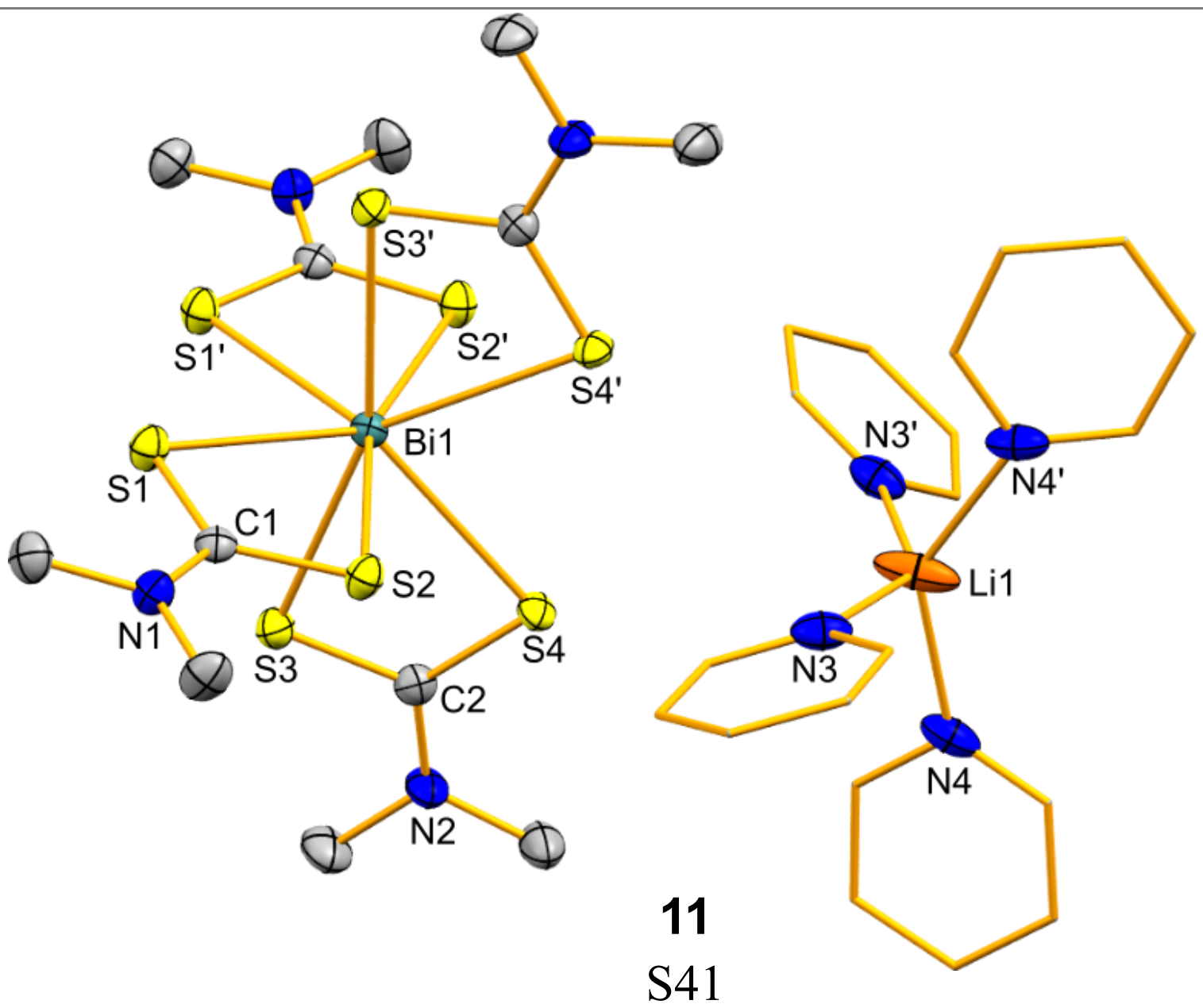

11

Table S12: Crystal and refinement data for $[CoCp_2][S_2CNMe_2]$.

**Compound**	**$[CoCp_2][S_2CNMe_2]$**
CCDC Number	2583077
Empirical Formula	$C_{13}H_{16}CoNS_2$
Formula Weight [g·mol^{-1}]	309.32
Temperature [K]	100
Crystal System	orthorhombic
Space Group	*Pnna*
a [Å]	13.0348(8)
b [Å]	14.5752(8)
c [Å]	6.7745(4)
α [°]	90
β [°]	90
γ [°]	90
V [Å^3]	1287.05(13)
Z	4
ρ_{calc} [g cm^{-3}]	1.596
μ [mm^{-1}]	1.633
F(000)	640.0
Crystal Size [mm^3]	0.09 × 0.031 × 0.019
Used Radiation	Mo-Kα (λ = 0.71073)
$2\Theta_{min}$ / $2\Theta_{max}$	5.590 / 49.988
Index Ranges	$-15 \le h \le 13$, $-17 \le k \le 17$, $-8 \le l \le 8$
Reflections Collected	14148
Independent Reflections	1133 [R_{int} = 0.0677, R_{sigma} = 0.0310]
Data/Restraints/Parameters	1133 / 0 / 80
GooF on F^2	1.079
Final R Indices ($I \ge 2\sigma(I)$)	R_1 = 0.0450, wR_2 = 0.1054
Final R Indices (all data)	R_1 = 0.0571, wR_2 = 0.1093
Largest diff. Peak / Hole [e·Å^{-3}]	2.06 / −0.59

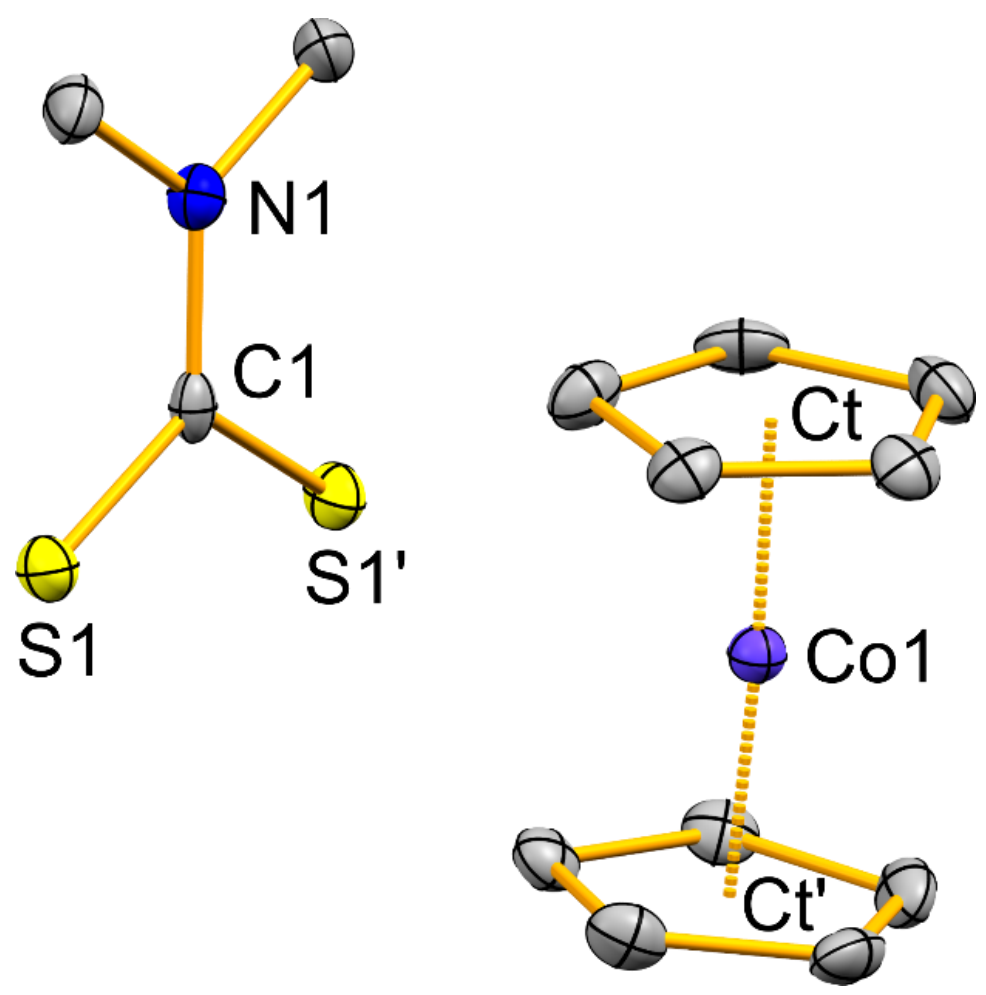

V. Solid-State Structure of $[Bi(NMe_2)_2Cl]_\infty$ (3-Cl)

In the solid state the halo(bismuth)amide **3-Cl** forms a 2D coordinationpolymer along the crystallographic a/b-plane, in which the chlorido ligands connect the $\{[Bi_2(NMe_2)_2](NMe_2)_2\}$ subunits in a μ_2-briding fashion. This is shown in Figure S54.

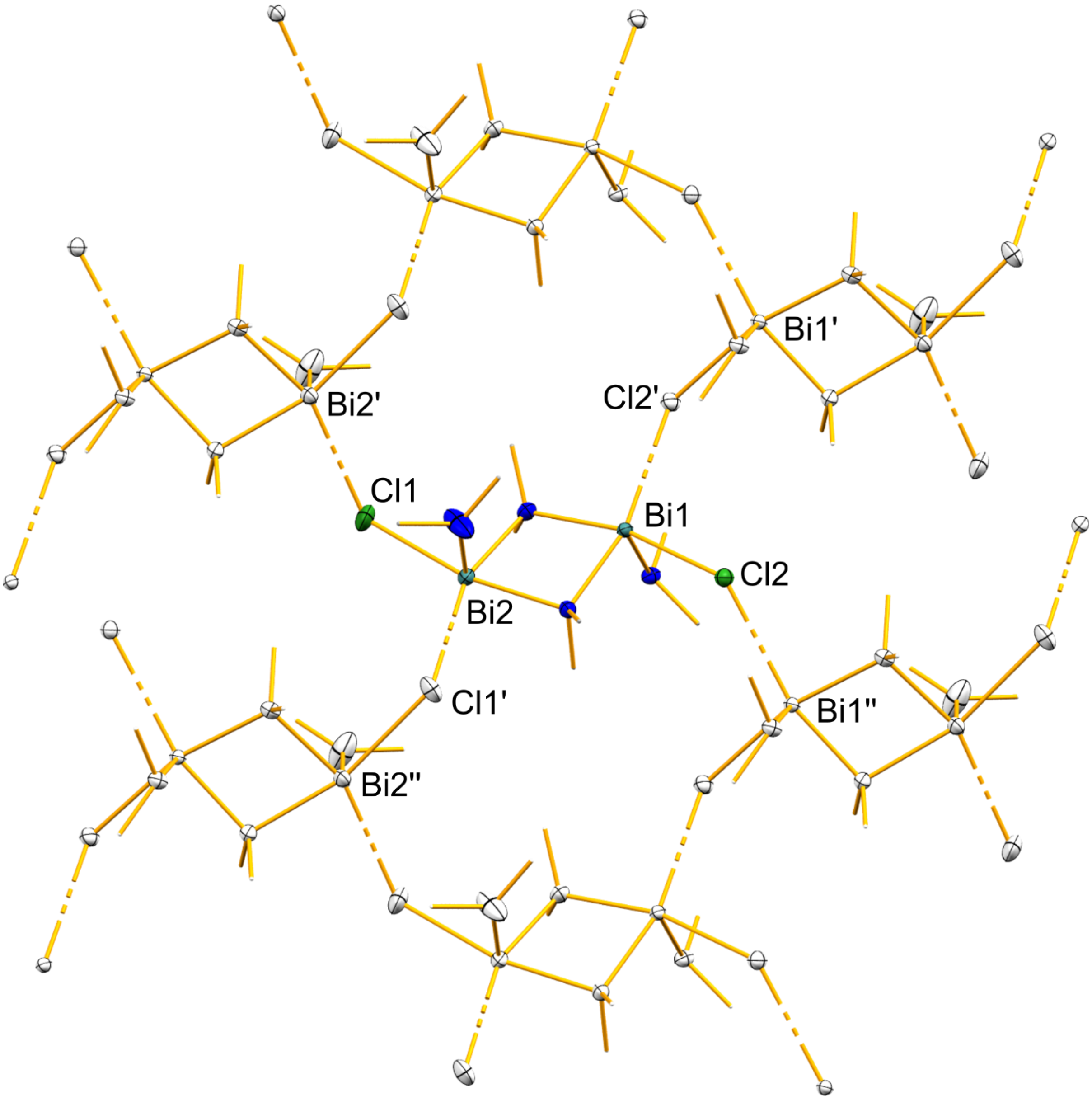

Figure S54: The molecular structure of **3-Cl** in the solid state. Displacement ellipsoids are shown at 50% probability, hydrogen atoms are omitted for clarity, carbon atoms are shown as wireframe. All atoms that do not belong to the asymmetric unit are shown in white. Relevant bonding parameters are given in the main text.

VI. Solid-State Structure of $[Bi(S_2CNMe_2)_3]_2$ (6)

The synthesis and structural characterization of compound **6** has already been object to investigations, but diffraction data have been collected at ambient temperature.[5–8] In order to provide a more precise basis for comparison, the diffraction data has been collected at a temperature of 100 K. The molecular structure of **6** in the solid state and relevant bonding parameters are shown in Figure S55.

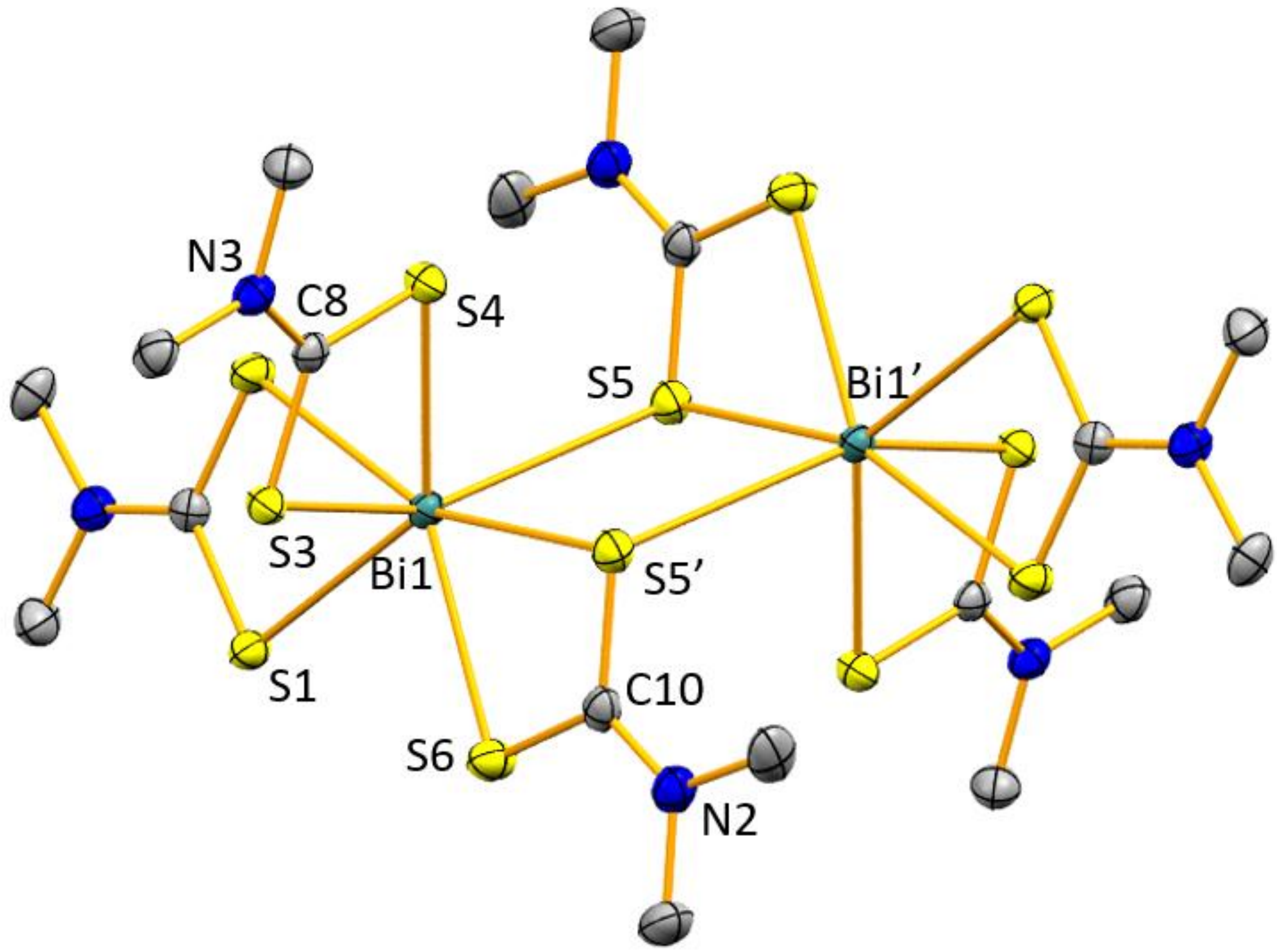

Figure S55: The molecular structure of **6** in the solid state. Displacement ellipsoids are shown at 50% probability, hydrogen atoms are omitted for clarity. Selected bond distances [Å] and bond angles [°]: Bi1-S1 2.848(2), Bi1-S2 2.806(2), Bi1-S3 2.601(2), Bi1-S4 2.816(3), Bi1-S5 2.918(2), Bi1-S6 2.858(3); S1-Bi1-S5 138.50(6), S1-Bi1-S6 77.16(8), S2-Bi1-S1 63.67(8), S2-Bi1-S4 78.63(7), S2-Bi1 S5 156.10(5), S2-Bi1-S6 140.82(6), S3 Bi1 S1 87.54(8), S3-Bi1-S2 86.76(7), S3-Bi1 S4 67.13(8), S3-Bi1-S5 85.87(7), S4-Bi1 S5 77.56(7).

VII. Solid-State Structure of $[CoCp_2][S_2CNMe_2]$

During the reduction of *in situ* generated $[Bi(CAAC)(S_2CNMe_2)_2I]$ with $CoCp_2$ in THF at −40 °C, $[CoCp_2][S_2CNMe_2]$ was crystallized out of the reaction mixture by slow evaporation of THF at −40 °C. The amount of crystalline material proofed not to be sufficient for the determination of a yield, further spectroscopic studies or elemental analyses. Nontheless, the crystalline material was analyzed by sc-XRD. The molecular structure of $[CoCp_2][S_2CNMe_2]$ in the solid state was unambiguously determined and is shown in Figure S56.

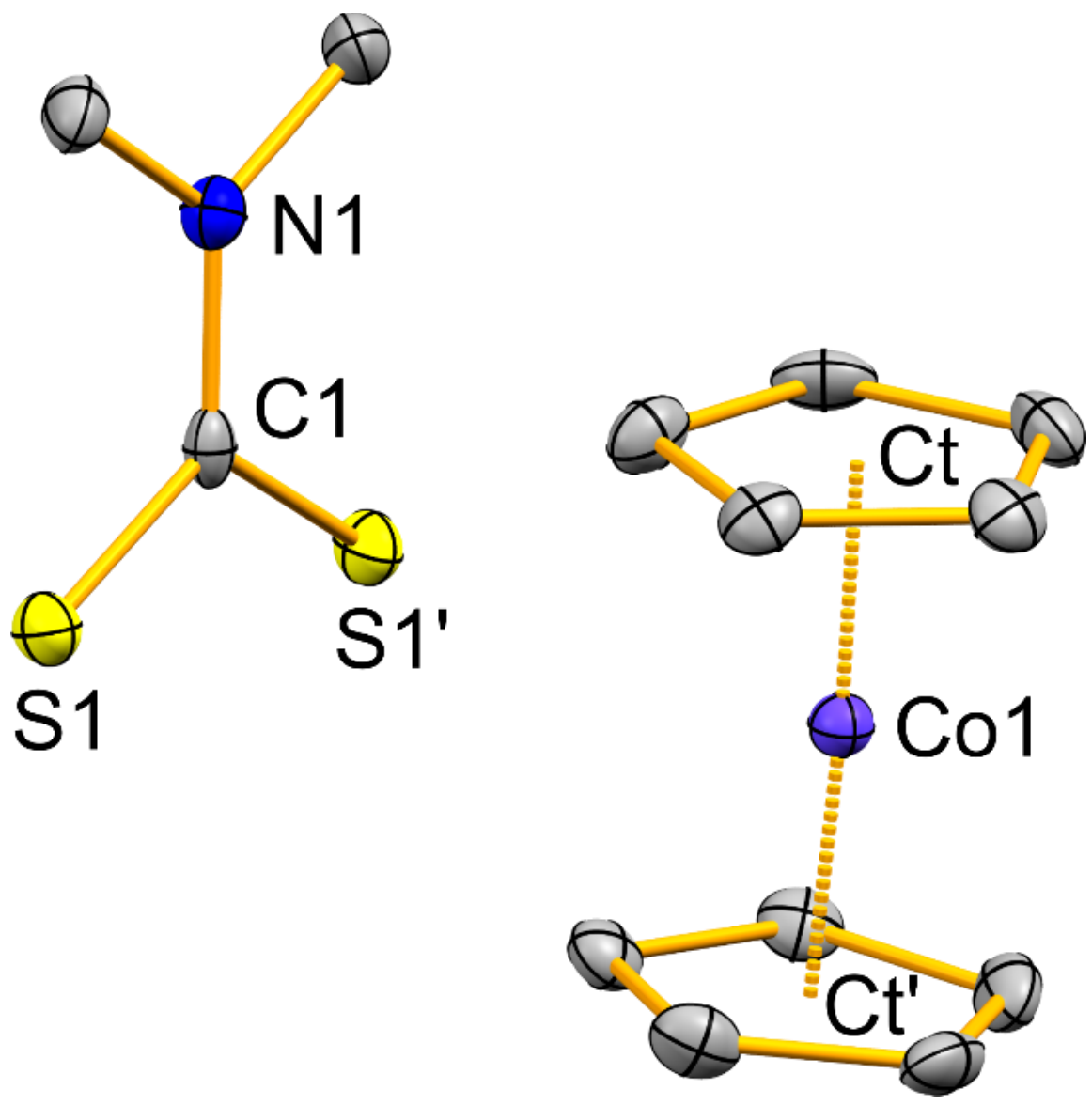

Figure S56: The molecular structure of $[CoCp_2][S_2CNMe_2]$ in the solid state. Displacement ellipsoids are shown at 50% probability, hydrogen atoms are omitted for clarity. Ct = centroid of Cp^- ligand. Selected bond distances [Å] and bond angles [°]: Co1-Ct 1.638, C1-S1 1.713(3), C1-N1 1.361(7), Ct-Co1-Ct' 176.8, S1-C1-S1' 123.0(4).

VIII. Solid-State Structure of $[Bi(CAAC)Cl_2(S_2CNMe_2)_3]_2$ (7)

The molecular structure of **7** in the solid state was investigated by single-crystal X-ray diffraction. A dinuclear aggregate was observed in the solid state due to weak intermolecular Bi···Cl contacts (Figure S57). When taking these intermolecular contacts into account, a distorted octahedral coordination geometry can be assigned to the central atom. A significant thermodynamic *trans*-influence is observed. This results in one shorter Bi–S bond (2.60 Å; *trans* to a Cl ligand of the neighboring formula unit) and one longer Bi–S bond (2.86 Å; *trans* to the ^{Me2}CAAC ligand) as well as one shorter Bi–Cl bond (2.59 Å, terminal) and one longer Bi–Cl bond (2.85 Å, bridging). The structural parameters are not discussed in further detail, since the data suffers from large rest electron density (5.93 eÅ^{-3}) close to the bismuth atom, which could not be accounted for by absorption correction.

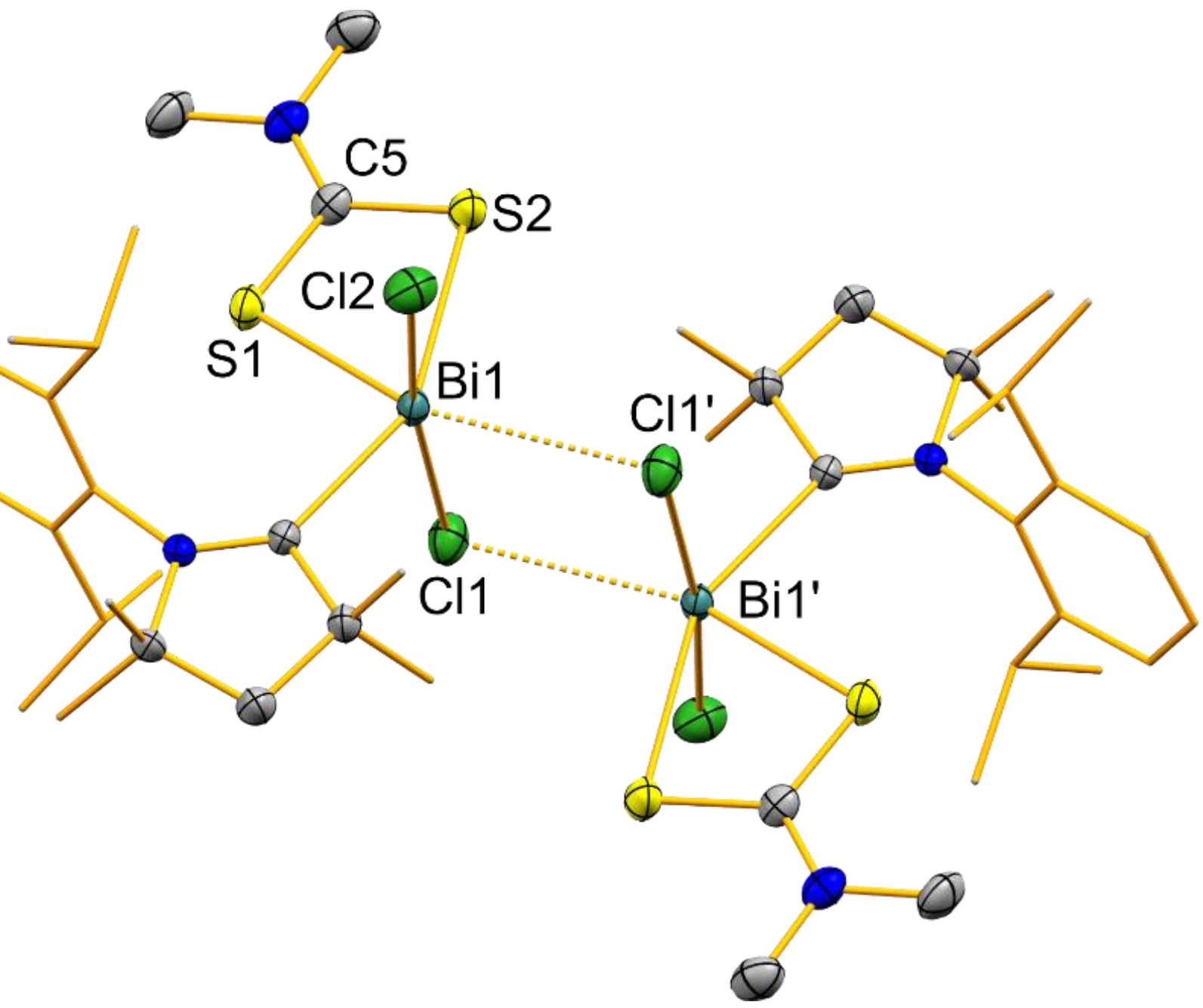

Figure S57: The molecular structure of **7** in the solid state. Ellipsoids are shown at 50% probability, hydrogen atoms are omitted for clarity, carbene bound methyl and 2,6-di*iso*propylphenyl substituents are shown as wireframe. Structural parameters are not discussed in detail due to large rest electron density (>5%) close to the bismuth atom.

IX. References

(1) Sheldrick, G. M. Crystal structure refinement with SHELXL. *Acta Cryst. C* **2015**, *71* (1), 3–8. DOI: 10.1107/S2053229614024218.

(2) Sheldrick, G. M. SHELXT - integrated space-group and crystal-structure determination. *Acta Cryst. A* **2015**, *71* (1), 3–8. DOI: 10.1107/S2053273314026370.

(3) Dolomanov, O. V.; Bourhis, L. J.; Gildea, R. J.; Howard, J. A. K.; Puschmann, H. OLEX2: a complete structure solution, refinement and analysis program. *J. Appl. Crystallogr.* **2009**, *42* (2), 339–341. DOI: 10.1107/S0021889808042726.

(4) Schmitz, W. International Tables for X-ray Crystallography, Vol. IV. *Krist. Techn.* **1975**, *10* (11), 23–4753. DOI: 10.1002/crat.19750101116.

(5) Ando, F.; Hayashi, T.; Ohashi, K.; Koketsu, J. Preparations and reactions of tris(dialkylamino)bismuthine. *J. Inorg. Nucl. Chem.* **1975**, *37* (9), 2011–2013. DOI: 10.1016/0022-1902(75)80943-2.

(6) Mustafa, I. A.; Taqa, A. A. Coordination compounds of tin and bismuth. Complexes of tin(IV) and bismuth(III) with N,N-dialkyldithiocarbamates. *Synth. React. Inorg. Met. Org. Chem.* **2001**, *31* (4), 517–526. DOI: 10.1081/SIM-100104783.

(7) Yin, H.; Li, F.; Wang, D. Synthesis and crystal structure of two new Bi(III) complexes $\{(Me_2NCS_2)_3Bi\}_2$ and $\{[(CH_2)_5NCS_2]_2BiI\}_2$. *J. Coord. Chem.* **2007**, *60* (11), 1133–1141. DOI: 10.1080/00958970601008846.

(8) Guo, Y.; Sun, R.; Chen, S.; Feng, Y. Syntheses, Crystal Structures and Antibacterial Activities of Arsenic and Bismuth Dialkyldithiocarbamate Complexes. *Chin. Chem. Bull. (Huaxue Tongbao)* **2014**, *77* (4), 365–369.
